# Learning quantum states of continuous-variable systems

**DOI:** 10.1038/s41567-025-03086-2

**Published:** 2025-11-26

**Authors:** Francesco A. Mele, Antonio A. Mele, Lennart Bittel, Jens Eisert, Vittorio Giovannetti, Ludovico Lami, Lorenzo Leone, Salvatore F. E. Oliviero

**Affiliations:** 1 NEST, Scuola Normale Superiore and Istituto Nanoscienze, Pisa, Italy; 2 Dahlem Center for Complex Quantum Systems, Freie Universität Berlin, Berlin, Germany; 3 Helmholtz-Zentrum Berlin für Materialien und Energie, Berlin, Germany; 4 NEST, Scuola Normale Superiore and Istituto Nanoscienze, Consiglio Nazionale delle Ricerche, Pisa, Italy; 5 QuSoft, Amsterdam, the Netherlands; 6 Korteweg-de Vries Institute for Mathematics, University of Amsterdam, Amsterdam, the Netherlands; 7 Institute for Theoretical Physics, University of Amsterdam, Amsterdam, the Netherlands; 8 Scuola Normale Superiore, Pisa, Italy

**Keywords:** Quantum information, Theoretical physics, Imaging and sensing

## Abstract

Quantum measurements are probabilistic and, in general, provide only partial information about the underlying quantum state. Obtaining a full classical description of an unknown quantum state requires the analysis of several different measurements, a task known as quantum-state tomography. Here we analyse the ultimate achievable performance in the tomography of continuous-variable systems, such as bosonic and quantum optical systems. We prove that tomography of these systems is extremely inefficient in terms of time resources, much more so than tomography of finite-dimensional systems such as qubits. Not only does the minimum number of state copies needed for tomography scale exponentially with the number of modes, but, even for low-energy states, it also scales unfavourably with the trace-distance error between the original state and its estimated classical description. On a more positive note, we prove that the tomography of Gaussian states is efficient by establishing a bound on the trace-distance error made when approximating a Gaussian state from knowledge of the first and second moments within a specified error bound. Last, we demonstrate that the tomography of non-Gaussian states prepared through Gaussian unitaries and a few local non-Gaussian evolutions is efficient and experimentally feasible.

## Abstract (web summary)

Finding a classical description of a quantum state can require resource-intensive tomography protocols. It has now been shown that, for bosonic systems, tomography is extremely inefficient in general, but can be done efficiently for some useful states.

## Main

A fundamental task in quantum physics, known as quantum-state tomography^1^, is to derive a classical description of a quantum system from experimental data. This is not only a powerful method for investigating Nature but also a diagnostic tool for benchmarking and verifying quantum devices^1–4^. The concept of tomography dates back to the 1990s^5,6^, when it was first introduced for continuous-variable (CV) systems^7^ and then for finite-dimensional systems. Over the years, tomography algorithms for CV systems have been extensively developed, so that it has become a bread-and-butter tool in quantum optics^5,6^.

In the last decade, rapid advances in accurately preparing quantum states^8^ have spurred the development of a new research field known as quantum learning theory^1^, which investigates how we can learn the properties of quantum systems as efficiently as possible. When the focus is on obtaining a full classical description of a quantum system, such an investigation reduces to quantum-state tomography, which is considered the cornerstone of quantum learning theory^1^. Despite tomography being a long-standing concept, many of its fundamental properties have only recently been unveiled due to advances in this new field, albeit only for finite-dimensional systems and not for CV systems.

Let us introduce quantum-state tomography from a quantum learning theory perspective. Formally, the task of quantum-state tomography involves two parameters: the number of state copies *N* and the error parameter *ε*. Given *N* copies of an unknown state, the goal is to output a classical description of a state that is guaranteed to be *ε*-close to the true unknown state with high probability (this is stated rigorously in Methods).

Among various notions of distance for measuring *ε*-closeness, the trace distance is the most popular due to its operational meaning^9,10^: given two states *ε*-close in trace distance, no quantum measurement can distinguish them by more than *ε*. Thus, the two states are, in fact, indistinguishable to any physical observer within a resolution *ε*. The optimal performance of tomography is quantified by the sample complexity, which is the minimum number of copies *N* needed to achieve tomography with error *ε*. When the sample complexity and running time of the tomography procedure scale polynomially with the number of constituents (for example, qubits and modes), the tomography is deemed efficient.

With vast many-body systems naturally emerging in Nature on one side and the rapid advances in constructing large-scale quantum devices on the other, minimizing the resources required has become imperative. Consequently, determining the sample complexity in the tomography of physically relevant classes of states is presently a pressing problem^1^. Notably, the sample complexity has been determined for *n*-qubit systems^1,11–14^: the tomography of mixed states requires *Θ*(4^*n*^/*ε*^2^) copies, whereas for pure states, the required number of copies reduces to *Θ*(2^*n*^/*ε*^2^), where *Θ* denotes the asymptotic scaling—that is, the number of copies grows proportionally to this expression up to constant factors.

Although quantum learning theory has been extensively developed for finite-dimensional systems, it remains almost unexplored for CV systems^15–18^. This is partly because the usual approaches to CV tomography are based on mere approximations of phase-space functions^5–7^, which do not account for the trace-distance error and, thus, lack operational meaning. This is a pressing gap in the literature, especially considering that in recent years, photonic quantum devices have been at the forefront of attempts to demonstrate quantum advantage, particularly through boson sampling^19–21^ and quantum simulation experiments^22^. Moreover, photonic platforms play a pivotal role in various quantum technologies, including quantum computation^23–27^, communication^28–33^ and sensing^34–37^. Therefore, determining the ultimate achievable performance of CV tomography has become an interesting problem, which we solve in this work.

## Energy-constrained states

**Fig. 1:**
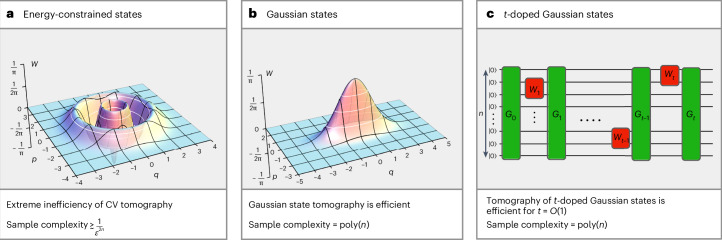
Quantum state tomography of relevant classes of CV quantum states. **a**, Quantum-state tomography of CV systems subject to energy constraints inherent in experimental platforms. Here, *n* is the number of modes, and *ε* is the trace-distance error. Our investigation reveals a phenomenon dubbed ‘extreme inefficiency’ of CV quantum-state tomography. Specifically, the number of copies required for the tomography of *n*-mode energy-constrained states must scale at least as *ε*^−2*n*^. This substantial scaling is a unique feature of CV systems, standing in stark contrast to finite-dimensional systems, where the required number of copies scales with the trace-distance error as *ε*^−2^. Therefore, we ask whether there exist physically interesting classes of states for which tomography is efficient. **b**, We answer this in the affirmative by presenting an efficient algorithm for the tomography of Gaussian states with provable guarantees in trace distance. Our analysis is based on technical tools of independent interest. Specifically, we introduce simple bounds on the trace distance between two Gaussian states in terms of the norm distance between their first moments and covariance matrices. **c**, Finally, we demonstrate that the tomography of non-Gaussian states prepared by Gaussian unitaries and a few local non-quadratic Hamiltonian evolutions is still efficient. Notably, both of these efficient tomography algorithms are experimentally feasible to implement in quantum optics laboratories.

A CV system corresponds to *n* modes, each associated with an infinite-dimensional Hilbert space. From the outset, the infinitely many degrees of freedom of a single bosonic mode make tomography a challenging concept. What does it mean for a tomography algorithm to output a classical description of an infinite-dimensional state? Does this imply that the output would be an infinite-dimensional matrix, which no classical computer could store? After all, Nature hardly deals with true infinities. Physical quantum states are always subject to certain constraints, the most fundamental of which is energy. In practice, Nature can produce only states with finite energy. Moreover, all states produced in quantum optical platforms—such as those used in optical quantum computing^19–21^ or fibre-optic communication^30,38,39^—have inherently bounded energy. Crucially, energy is also a property that experimentalists can often estimate with good accuracy and typically have prior information about. In light of all this, we can think of CV quantum states as physically meaningful only under an energy constraint. Including such a constraint turns the task of tomography from unavoidably inconceivable to a precious tool for investigating bosonic quantum systems. As explained below, we can, indeed, devise tomography algorithms capable of achieving an arbitrarily low trace-distance error for infinite-dimensional states subjected to some energy constraint (Fig. 1).

By definition, an *n*-mode state *ρ* satisfies the energy constraint *E* if the expectation value of the energy observable satisfies

1 $${\rm{Tr}}[{\hat{E}}_{n}\rho ]\le nE.$$

Here the energy observable is defined as

2 $${\hat{E}}_{n}:=\frac{1}{2}\sum_{i=1}^{n}\left({\hat{p}}_{i}^{2}+{\hat{x}}_{i}^{2}\right),$$

where ${\{{\hat{x}}_{i}\}}_{i = 1}^{n}$ and ${\{{\hat{p}}_{i}\}}_{i = 1}^{n}$ denote the position and momentum operators of the *n* modes^7^. We normalize the right-hand-side of equation (1) with *n* because the energy is an extensive observable. Note that the energy constraint in equation (1) is equivalent to a constraint on the mean total photon number of the state (equation (18)).

The following theorem, proven in Supplementary Theorem 26, determines the sample complexity in the tomography of energy-constrained pure states.

### Theorem 1

(Tomography of energy-constrained pure states) *The sample* *complexity in the tomography of n-mode pure states is* *Θ*(*E*^*n*^/*ε*^2*n*^). *Here ε is the trace-distance error and E is the energy constraint*.

**Fig. 2:**
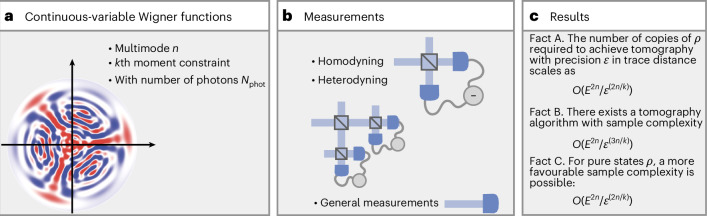
Efficiency of quantum state tomography of CV systems. **a**, We establish fundamental bounds on the resources required for quantum-state tomography of CV *k*th-moment-constrained quantum states, highlighting the pronounced inefficiency of any strategy aiming to solve this task. **b**, Our results encompass any possible strategy, including those using only homodyne and heterodyne measurements, as well as other experimentally feasible operations in photonic platforms, and even general measurements. This means, independently of the techniques used, the tomography of CV states is impractical. **c**, We identify three key results, labelled facts A–C. The implication is that the resources needed for tomography exhibit strong dependence on the desired accuracy, scaling as ~*ε*^−2*n*/*k*^.

This result establishes that *every* tomography algorithm—even standard algorithms based on homodyne and heterodyne detections (Fig. 2)—must use at least *Ω*(*E*^*n*^/*ε*^2*n*^) copies to achieve error *ε*, where *Ω* denotes an asymptotic lower bound, meaning that the number of required copies cannot grow slower than this quantity up to constant factors. Conversely, it also establishes that there exists a tomography algorithm that achieves an error *ε* given access to the above number of copies.

This finding reveals a striking phenomenon that we dub the ‘extreme inefficiency’ of CV tomography: not only does the number of copies required for CV tomography scale exponentially with the number of modes *n*, as for finite-dimensional systems, but it also has a substantial scaling with respect to the error *ε*. Specifically, the scaling of ~*ε*^−2*n*^ is a unique feature of CV tomography, being in stark contrast with the finite-dimensional setting characterized by the scaling of ~*ε*^−2^. Although in the finite-dimensional setting the error can be halved by increasing the number of copies by a factor of 4, which is cheap, in the CV setting, one needs an exponential factor 4^*n*^, which is costly.

To emphasize this notable behaviour, let us illustrate with an example the substantial difference between the tomography of finite-dimensional systems and the tomography of CV systems. We will estimate the time required to achieve tomography with error *ε* = 10% in both settings. Assume that each copy of the state is produced and processed every 1 ns (typical for qubits and light pulses). Then, the tomography of ten-qubit pure states requires just ~0.1 ms. However, as a consequence of Theorem 1, the tomography of ten-mode pure states with a mean number of photons per mode ≤1 requires at least ~3,000 years, which shows that CV tomography becomes impractical even for a few modes. This highlights that the tomography of CV systems is extremely inefficient, much more so than the tomography of finite-dimensional systems.

In Theorem 2, proven in Supplementary Information, we find bounds on the sample complexity in the tomography of energy-constrained mixed states.

### Theorem 2

(Tomography of energy-constrained mixed states) The number of copies *O*(*E*^2*n*^/*ε*^3*n*^) *is sufficient to achieve the tomography of n-mode mixed states. Conversely, the tomography of such states requires at least* *Ω*(*E*^2*n*^/*ε*^2*n*^) *copies. Here, ε is the trace-distance error and E is the energy constraint*.

For a rigorous presentation of the asymptotic notation used, we refer the reader to Supplementary Information Section 1. The core idea underpinning the attainable (yet costly) performance guarantees for tomography presented in Theorem 2 is that energy-constrained CV states are effectively described by finite-dimensional systems. In particular, we prove that any (possibly mixed) energy-constrained state can be approximated, up to trace-distance error *ε*, by a *D*-dimensional state with rank *r* such that

3 $$\begin{aligned}D&=O({E}^{n}/{\varepsilon }^{2n}),\\ r&=O({E}^{n}/{\varepsilon }^{n}).\end{aligned}$$

The first result of Theorem 2 simply follows from the observation that the sample complexity in the tomography of *D*-dimensional states with rank *r* is *O*(*Dr*) (refs. ^1,11–14^). Notably, equation (3) establishes that physical CV states—those with bounded energy—are characterized by a finite number of physically relevant parameters.

These conclusions allow us to shed light on the core question in CV tomography posed at the beginning of the section: the tomography of arbitrary CV states is, indeed, meaningless considering that infinitely many parameters are physically inconceivable, but that with an energy constraint, tomography gains real-world relevance. Indeed, in this latter case, it is sufficient for the output to be a matrix of finite dimension, that is, *O*(*E*^*n*^/*ε*^2*n*^).

The above findings can be generalized by considering constraints on any higher moments of the energy. By definition, like equation (1), an *n*-mode state *ρ* satisfies the *k*-moment constraint *E* if

4 $${\left({\rm{Tr}}\!\left[\hat{E}_{n}^{\,k}\rho \right]\right)}^{1/k}\le nE.$$

As proven in Supplementary Information, the sample complexity in the tomography of pure states becomes *Θ*(*E*^*n*^/*ε*^2*n*/*k*^), whereas for mixed states, the sample complexity is upper bounded by *O*(*E*^2*n*^/*ε*^3*n*/*k*^) and lower bounded by *Ω*(*E*^2*n*^/*ε*^2*n*/*k*^), thus recovering Theorems 1 and 2 for *k* = 1. Notably, the sample complexity decreases as *k* increases, and the substantial scaling ~*ε*^−2*n*/*k*^ disappears for *k* ≳ *n*.

## Gaussian states

So far, we have identified strong limitations on the tomography of arbitrary unknown CV states, even under stringent energy constraints. However, in many practical scenarios, another prior assumption about the unknown CV state is often available, namely, that it is Gaussian. Gaussian states are the most commonly used states in quantum optics laboratories and have key applications in quantum sensing, communication and computing^7^. This prompts a natural question: can the tomography of Gaussian states be efficiently performed? Here, we answer this question affirmatively.

We start by investigating the following problem, rather fundamental for the field of CV quantum information. It is well known that Gaussian states are in a one-to-one correspondence with their first moments and covariance matrices^7^. However, as in practice one has access only to a finite number of copies of an unknown Gaussian state, it is impossible to determine its first moment and covariance matrix exactly. Instead, one can obtain only arbitrarily good approximations of them. Given the operational meaning of the trace distance^9,10^, the following question is, thus, a fundamental problem: If we know with an error *ε* the first moment and the covariance matrix of an unknown Gaussian state, what is the resulting trace-distance error that we make in approximating the underlying state? Theorem 3, proven in Methods, answers this question.

### Theorem 3

(Error propagation from moments to trace distance) *If we know with an error ε the first moment and the covariance matrix of an unknown Gaussian state, the resulting trace-distance error is at most* $O(\sqrt{\varepsilon })$ *and at least* *Ω*(*ε*).

To prove this result, Theorems 10 and 11 place stringent bounds on the trace distance between two Gaussian states in terms of the norm distance between their first moments and covariance matrices, which constitute technical tools of independent interest for the field of CV quantum information.

Notably, Theorem 3 is the key tool in proving the main result of this section: the tomography of Gaussian states is efficient. Specifically, Theorem 4, proven in Supplementary Information, shows that the sample complexity in the tomography of Gaussian states scales polynomially with the number of modes.

### Theorem 4

(Tomography of Gaussian states) *There exists an algorithm that uses*

5 $$O\left(\frac{{n}^{7}{E}^{4}}{{\varepsilon }^{4}}\right)={\rm{poly}}(n)$$

*copies to achieve the tomography of an unknown n-mode Gaussian state. Here, ε is the trace-distance error and E is the energy constraint*.

This algorithm simply consists of estimating the first moment and the covariance matrix of the unknown Gaussian state, both procedures routinely performed in quantum optics laboratories using homodyne detection^7^. Additionally, the algorithm runs in poly(*n*) time, and its output is efficient to store, as it consists only of the *O*(*n*^2^) parameters of the first moment and covariance matrix. A similar tomography algorithm has been proposed in the fermionic Gaussian setting in the particular case in which the state is pure^40,41^.

In summary, we have established the efficiency of the tomography of Gaussian states. However, what if the state deviates slightly from being exactly Gaussian? This is a crucial question, especially considering imperfections during state preparation in experimental set-ups. In Supplementary Information, we demonstrate that our tomography algorithm is robust against little perturbations caused by non-Gaussian noise (for example, dephasing noise). Even if the unknown state is a slightly perturbed Gaussian state—technically, a state with a sufficiently small relative entropy of non-Gaussianity^42^—our algorithm remains effective and tomography remains efficient.

## *t*-doped Gaussian states

Having demonstrated that the tomography of arbitrary non-Gaussian states is extremely inefficient (Theorem 1) and that the tomography of Gaussian states is efficient (Theorem 4), we now seek to interpolate between these two regimes. This leads us to analyse *t*-doped Gaussian states, states prepared by applying Gaussian unitaries and at most *t* non-Gaussian local unitaries on the vacuum state. The set of all *t*-doped Gaussian states coincides with that of all pure Gaussian states for *t* = 0, it grows as *t* increases, and it becomes the set of all pure states for *t* → ∞ (ref. ^43^). In this sense, analysing the performance of tomography as a function of *t* allows us to understand the trade-off between the efficiency of tomography and the degree of ‘non-Gaussianity’ of the unknown bosonic quantum state. The tomography of *t*-doped states has been thoroughly investigated in both the stabilizer^44–50^ and fermionic^41^ settings. In this section, we present a bosonic generalization of the results established in these earlier works.

**Fig. 3:**
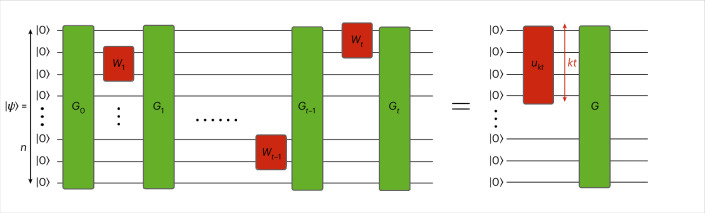
Pictorial representation of a *t*-doped Gaussian state. By definition, a *t*-doped Gaussian state vector |*ψ*〉 is a state prepared by applying Gaussian unitaries *G*_0_, …, *G*_*t*_ (green boxes) and at most *t* non-Gaussian *κ*-local unitaries *W*_1_, …, *W*_*t*_ (red boxes) to the *n*-mode vacuum. A unitary is said to be *κ*-local if it is generated by a Hamiltonian that is a polynomial in at most *κ* operators from the set of position operators ${\{{\hat{x}}_{i}\}}_{i = 1}^{n}$ and momentum operators ${\{{\hat{p}}_{i}\}}_{i = 1}^{n}$ of the *n* modes. Also shown is the decomposition given by Theorem 5, which establishes that all the non-Gaussianity in |*ψ*〉 can be compressed into a localized region consisting of *κt* modes by applying a Gaussian unitary *G*^†^ to |*ψ*〉.

Let us proceed with the mathematical definitions. A unitary *U* is a *t*-doped Gaussian unitary if it is a composition

6 $$U={G}_{t}{W}_{t}\cdots {G}_{1}{W}_{1}{G}_{0},$$

of Gaussian unitaries *G*_0_, *G*_1_, …, *G*_*t*_ and at most *t* (non-Gaussian) *κ*-local unitaries *W*_1_, …, *W*_*t*_, as depicted in Fig. 3. A unitary is *κ*-local if it is generated by a Hamiltonian that is a polynomial in at most *κ* quadratures. An *n*-mode state vector |*ψ*〉 is a *t*-doped Gaussian state vector if it can be prepared by applying a *t*-doped Gaussian unitary *U* to the vacuum as

7 $$\left\vert \psi \right\rangle =U\left\vert 0\right\rangle^{\otimes n}.$$

Theorem 5, proven in Supplementary Information, provides a notable decomposition of *t*-doped unitaries and states.

### Theorem 5

(Compression of non-Gaussianity) *If n* *≥* *κt, any n-mode t-doped Gaussian unitary U can be decomposed as*

8 $$U=G({u}_{\kappa t}\otimes {{\mathbb{1}}}_{n-\kappa t}){G}_{{\rm{passive}}},$$

*for some suitable Gaussian unitary G, energy-preserving Gaussian unitary G*_*passive*_ *(ref.* ^7^*) and κt-mode (non-Gaussian) unitary u*_*κt*_*. In particular, any n-mode t-doped Gaussian state vector can be decomposed as*

9 $$\left\vert \psi \right\rangle =G\left(\left\vert {\phi }_{\kappa t}\right\rangle \otimes {\left\vert 0\right\rangle }^{\otimes (n-\kappa t)}\right),$$

*for some suitable Gaussian unitary G and κt-mode (non-Gaussian) state vector* |*φ*_*κt*_〉.

Equation (9) establishes that all the non-Gaussianity of a *t*-doped Gaussian state can be compressed into *O*(*t*) modes through a suitable Gaussian unitary. This result can be seen as a bosonic counterpart of recent results within the stabilizer^44–50^ and fermionic setting^41^, which reveals a fascinating parallelism among the theories of stabilizers, fermions and bosons.

Leveraging the decomposition in equation (9), we design a simple and experimentally feasible tomography algorithm for *t*-doped Gaussian states. Our algorithm (1) estimates the first moment and the covariance matrix to construct an estimate of the Gaussian unitary *G*, (2) applies its inverse to the state to compress the non-Gaussianity into the first *κt* modes and (3) performs full-state tomography on the first *κt* modes (see Supplementary Information for details). This algorithm is practical because it requires only tools commonly available in quantum optics laboratories, such as Gaussian evolutions and easily implementable Gaussian measurements, like homodyne and heterodyne detection^7^. Specifically, step (3) can be achieved using the CV classical shadow algorithm^17^, which is experimentally feasible. Alternatively, to obtain a tighter upper bound on the sample complexity in the tomography of *t*-doped Gaussian state, step (3) can be performed using the optimal full-state tomography algorithm identified in Theorem 1.

Theorem 6, proven in Supplementary Information, analyses the performance of our tomography algorithm. On a technical note, deriving guarantees on the estimate of the covariance matrix in step (1) requires more than just an energy constraint; a second-moment constraint is also necessary. Refer to equation (4) for the definition of a second-moment constraint.

### Theorem 6

(Tomography of a *t*-doped Gaussian state) *There exists an algorithm that exploits*

10 $$\operatorname{poly}(n)+O\left({\left(nE/\varepsilon \right)}^{2\kappa t}\right)$$

*copies to achieve the tomography of n-mode t-doped Gaussian states. Here, E is the second-moment constraint, ε is the trace-distance error and κ is the locality of the non-Gaussian unitaries*.

This theorem implies that the sample complexity in the tomography of *t*-doped Gaussian states scales at most exponentially with *κt*, indicating that tomography becomes increasingly difficult as the degree of non-Gaussianity *t* increases. Additionally, both the time and memory complexities exhibit the same behaviour. Notably, this establishes the core finding of this section: the tomography of *t*-doped Gaussian states is efficient in the regime *κt* = *O*(1).

The proof of Theorem 6 hinges on the fact that a *t*-doped state is compressible, meaning that it satisfies the decomposition in equation (9). Specifically, we can prove that the tomography of compressible states is efficient if and only if *κt* = *O*(1). This contrasts with the stabilizer^44,49,50^ and fermionic setting^41^, where the tomography of compressible stabilizer and fermionic states is efficient if and only if *t* = *O*(log(*n*)). The difference in our setting ultimately arises from the infinite-dimensional nature of CV states and the presence of energy constraints.

## Discussion

Our work serves as bridge between quantum learning theory and CV quantum information. We have conducted an exhaustive investigation of the tomography of CV systems with guarantees on the trace-distance error.

First, we have determined the sample complexity in the tomography of energy-constrained pure states, which pinpoints the ultimate achievable performance of CV tomography. However, the ‘extreme inefficiency’ of CV tomography emerged: any tomography algorithm for energy-constrained states must use a number of copies that substantially scales at least as *ε*^−2*n*^, where *n* is the number of modes and *ε* is the trace-distance error. This phenomenon, which provides costly fundamental limitations even for small *n*, is a unique feature in the tomography of CV systems.

On a more positive note, we have proven that the tomography of Gaussian states is efficient. To establish this, we investigated a fundamental problem of CV quantum information: how the error in approximating the first moment and the covariance matrix of a Gaussian state propagates in the trace-distance error. Our solution introduces tools of independent interest: simple, stringent bounds on the trace distance between two Gaussian states in terms of the norm distance between their first moments and covariance matrices.

Finally, we have devised an experimentally feasible algorithm that efficiently achieves the tomography of *t*-doped Gaussian states for small *t*. This establishes that even if a few non-Gaussian local gates are applied to a Gaussian state, the tomography of the resulting non-Gaussian state remains efficient. The main tool employed here is a decomposition of *t*-doped Gaussian states, which shows that all the non-Gaussianity in the state can be compressed into only *O*(*t*) modes through a Gaussian unitary.

**Fig. 4:**
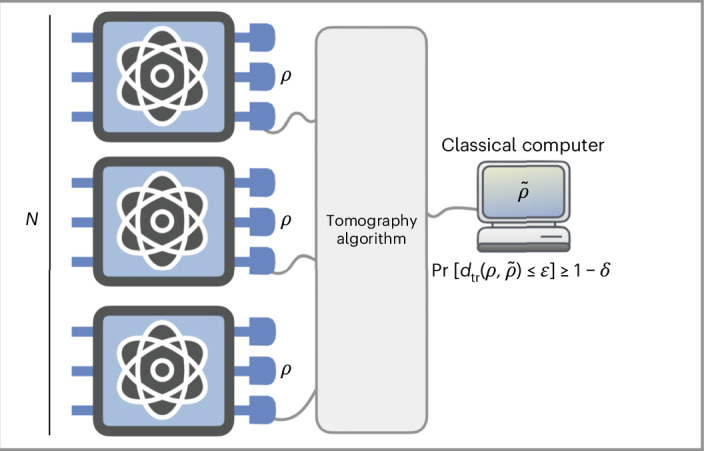
Pictorial representation of a quantum-state tomography algorithm. Given access to *N* copies of an unknown state *ρ*, the goal of a tomography algorithm is to output a classical description of a state $\tilde{\rho }$ that serves as a good approximation of the true unknown state *ρ*. Let us clarify what we mean by a ‘good approximation’. First, the error in this approximation is measured by the trace distance ${d}_{{\rm{tr}}}(\rho ,\tilde{\rho })$, which is most meaningful notion of distance between states^9,10^. Second, as quantum measurements yield probabilistic outcomes, the output $\tilde{\rho }$ is probabilistic rather than deterministic. Therefore, a ‘good approximation’ means that the probability of getting a small trace-distance error is high. This is expressed as $\Pr [{d}_{{\rm{tr}}}(\rho ,\tilde{\rho })\le \varepsilon ]\ge 1-\delta$, where *ε* is the trace-distance error and *δ* is the failure probability. To measure the performance of tomography, one can define the sample complexity. For fixed *ε* and *δ*, the sample complexity is the minimum number of copies *N* required to achieve tomography with trace-distance error *ε* and failure probability *δ*. For example, the sample complexity in the tomography of arbitrary *n*-qubit states is $O\left((4^{n}/\varepsilon^{2})\log (1/\delta)\right)$ (refs. ^1,12–14^). In general, the sample complexity in any tomography task depends at most logarithmically on the failure probability *δ* (ref. ^51^), implying that this parameter has a minimal impact on performance. In other words, the failure probability *δ* can be made very small with only a slight increase in the sample complexity. Therefore, throughout this work, we omit the dependence on *δ*.

## Methods

### Trace distance

The trace distance between two quantum states *ρ*_1_ and *ρ*_2_ is defined as

11 $${d}_{{\rm{tr}}}(\;{\rho }_{1},{\rho }_{2}):=\frac{1}{2}\|{\rho }_{1}-{\rho }_{2}\|_{1},$$

where $\|A\|_{1}:={\rm{Tr}}\sqrt{{A}^{\dagger }A}$ denotes the trace norm. The trace distance is considered the most meaningful notion of distance between two states because of its operational meaning in terms of the optimal probability of discriminating between two states having access to a single copy of the state (Holevo–Helstrom theorem^9,10^). Consequently, in quantum information theory, the error in approximating a state is typically quantified using the trace distance.

### Quantum-state tomography

In this section, we formulate precisely the problem of quantum-state tomography^1^, which forms the basis of our investigation. The basic set-up is depicted in Fig. 4.

#### Problem 7

(Quantum-state tomography) *Let* ${\mathcal{S}}$ *be a set of quantum states. Consider* *ε*, *δ* ∈ (0, 1) and $N\in {\mathbb{N}}$. Let $\rho \in {\mathcal{S}}$ *be an unknown quantum state. Given access to N copies of ρ, the goal is to provide a classical description of a quantum state* $\tilde{\rho }$ *such that*

12 $$\Pr \left[{d}_{{\rm{tr}}}(\;\tilde{\rho },\rho )\le \varepsilon \right]\ge 1-\delta .$$

*That is, with a probability* ≥1 − *δ*, *the trace distance between* $\tilde{\rho }$ *and ρ is at most ε. Here ε is called the trace-distance error, and δ is called the failure probability*.

The sample complexity, the time complexity and the memory complexity in the tomography of states in ${\mathcal{S}}$ are defined as the minimum number of copies *N*, the minimum amount of classical and quantum computation time, and the minimum amount of classical memory, respectively, required to solve Problem 7 with trace-distance error *ε* and failure probability *δ*. Note that the time complexity is always an upper bound on the memory complexity and the sample complexity.

One can think of ${\mathcal{S}}$ as a specific subset of the entire set of *n*-qubit states (for example, pure states, *r*-rank states and stabilizer states) or *n*-mode states (for example, energy-constrained states, moment-constrained states, Gaussian states and *t*-doped Gaussian states). By definition, tomography is deemed efficient if the sample, time and memory complexities scale polynomially in *n*; otherwise, it is deemed inefficient. For example, in our work, we prove that the tomography of energy-constrained states is (extremely) inefficient. By contrast, the tomography of Gaussian states is efficient, whereas the tomography of *t*-doped Gaussian states is efficient for small *t* and inefficient for large *t*.

### CV systems

In this section, we provide a concise overview of quantum information with CV systems^7^. A CV system is a quantum system associated with the Hilbert space ${L}^{2}({{\mathbb{R}}}^{n})$ of all square-integrable complex-valued functions over ${{\mathbb{R}}}^{n}$, which models *n* modes of electromagnetic radiation with definite frequency and polarization. A quantum state in ${L}^{2}({{\mathbb{R}}}^{n})$ is called an *n*-mode state, and a unitary operator in ${L}^{2}({{\mathbb{R}}}^{n})$ is called an *n*-mode unitary. The quadrature vector is defined as

13 $$\hat{{\bf{R}}}:={({\hat{x}}_{1},{\hat{p}}_{1},\ldots ,{\hat{x}}_{n},{\hat{p}}_{n})}^{\top },$$

where ${\hat{x}}_{j}$ and ${\hat{p}}_{j}$ are the well-known position and momentum operators of the *j*th mode, collectively called quadratures. Let us proceed with the definitions of a Gaussian unitary and a Gaussian state.

#### Definition 8

(Gaussian unitary) *An n-mode unitary is Gaussian if it is the composition of unitaries generated by quadratic Hamiltonians* $\hat{H}$ *in the quadrature vector*

14 $$\hat{H}:=\frac{1}{2}{(\hat{{\bf{R}}}-{\bf{m}})}^{\top }h(\hat{{\bf{R}}}-{\bf{m}}),$$

*for some symmetric matrix* $h\in {{\mathbb{R}}}^{2n,2n}$ *and some vector* ${\bf{m}}\in {{\mathbb{R}}}^{2n}$.

#### Definition 9

(Gaussian state) *An n-mode state ρ is Gaussian if it can be written as a Gibbs state of a quadratic Hamiltonian* $\hat{H}$ *of the form in equation* (14) *with h being positive definite*. *The Gibbs states associated with the Hamiltonian* $\hat{H}$ *are given by*

15 $$\rho ={\left(\frac{{e}^{-\beta \hat{H}}}{{\rm{Tr}}[\operatorname{e}^{-\beta \hat{H}}]}\right)}_{\beta \in [0,\infty ]},$$

*where the parameter* *β* *is the inverse temperature*.

This definition includes also the pathological cases where both *β* and certain terms of *H* diverge (for example, this is the case for tensor products between pure Gaussian states and mixed Gaussian states). An example of a Gaussian state vector is the vacuum, denoted as ${\left\vert 0\right\rangle }^{\otimes n}$. Any pure *n*-mode Gaussian state vector can be written as a Gaussian unitary *G* applied to the vacuum:

16 $$\left\vert \psi \right\rangle =G{\left\vert 0\right\rangle }^{\otimes n}.$$

A Gaussian state *ρ* is uniquely identified by its first moment **m**(*ρ*) and covariance matrix *V*(*ρ*). By definition, the first moment and the covariance matrix of an *n*-mode state *ρ* are given by

17 $$\begin{aligned}{\bf{m}}(\;\rho )&:={\rm{Tr}}\left[\hat{{\bf{R}}}\,\rho \right],\\ V(\;\rho )&:={\rm{Tr}}\left[\left\{(\hat{{\bf{R}}}-{\bf{m}}(\;\rho )),{(\hat{{\bf{R}}}-{\bf{m}}(\;\rho ))}^{\top }\right\}\rho \right],\end{aligned}$$

where $\{\hat{A},\hat{B}\}:=\hat{A}\hat{B}+\hat{B}\hat{A}$ is the anti-commutator.

By definition, the energy of an *n*-mode state *ρ* is given by the expectation value ${\rm{Tr}}[{\hat{E}}_{n}\rho ]$ of the energy observable ${\hat{E}}_{n}:=\sum_{j = 1}^{n}({\hat{x}}_{j}^{2}+{\hat{p}}_{j}^{2})/2$, where it is assumed that each mode has a frequency of one^7^. It is important to note that energy is an extensive quantity, because for any single-mode state *σ* the energy of *σ*^⊗*n*^ equals the energy of *σ* multiplied by *n*. Furthermore, the energy of an *n*-mode state is always greater than or equal to *n*/2, with the equality achieved only by the vacuum. The total number of photons can be defined in terms of the energy observable as

18 $${\hat{N}}_{n}:={\hat{E}}_{n}-\frac{n}{2}\hat{{\mathbb{1}}}.$$

Given ${\bf{k}}=({k}_{1},\ldots ,{k}_{n})\in {{\mathbb{N}}}^{n}$, let us denote as

19 $$\left\vert {\bf{k}}\right\rangle =\left\vert {k}_{1}\right\rangle \otimes \cdots \otimes \left\vert {k}_{n}\right\rangle$$

the *n*-mode Fock state vector^7^. The total number of photons is diagonal in the Fock basis as

20 $${\hat{N}}_{n}=\sum _{{\bf{k}}\in {{\mathbb{N}}}^{n}}\|{\bf{k}}\|_{1}\left\vert {\bf{k}}\right\rangle \left\langle {\bf{k}}\right\vert ,$$

where $$\|{\bf{k}}\|_{1}:=\sum_{i = 1}^{n}{k}_{i}$$.

### Effective dimension and rank of energy-constrained states

In this section, we show that energy-constrained states can be approximated well by finite-dimensional states with low rank, as anticipated above in equation (3). Further technical details regarding the findings presented in this section can be found in Supplementary Information.

Let *ρ* be an *n*-mode state with total number of photons satisfying the energy constraint

21 $${\rm{Tr}}[\;\rho {\hat{N}}_{n}]\le nE,$$

where *E* ≥ 0. Given $M\in {\mathbb{N}}$, let ${{\mathcal{H}}}_{M}$ be the subspace spanned by all the *n*-mode Fock states with total number of photons not exceeding *M*, and let *Π*_*M*_ be the projector onto this space.

Let us begin by analysing the effective dimension of the set of energy-constrained states. The trace distance between the energy-constrained state *ρ* and its projection *ρ*_*M*_ onto ${{\mathcal{H}}}_{M}$, that is,

22 $${\rho }_{M}:=\frac{{\varPi }_{M}\rho {\varPi }_{M}}{\operatorname{Tr}[{\varPi }_{M}\rho ]},$$

can be upper bounded as follows:

23 $${d}_{{\rm{tr}}}(\;\rho ,{\rho }_{M})\mathop{\le }\limits^{({\rm{i}})}\sqrt{{\rm{Tr}}[({\mathbb{1}}-{\Pi }_{M})\rho ]}\mathop{\le }\limits^{({\rm{ii}})}\sqrt{\frac{{\rm{Tr}}[{\hat{N}}_{n}\rho ]}{M}}\le \sqrt{\frac{nE}{M}},$$

where in (i) we have employed the gentle measurement lemma^52^ and in (ii) we used the simple operator inequality ${\mathbb{1}}-{\varPi }_{M}\le {\hat{N}}_{n}/M$. Consequently, by setting *M*_1_ := ⌈*nE*/*ε*^2^⌉, it follows that the projection ${\rho }_{{M}_{1}}$ is *ε*-close to *ρ* in trace distance. Moreover, the dimension of ${{\mathcal{H}}}_{{M}_{1}}$ can be upper bounded as

24 $$\dim {{\mathcal{H}}}_{{M}_{1}}=\left(\begin{array}{c}n+{M}_{1}\\ n\end{array}\right)\le {\left(\frac{\mathrm{e}(n+{M}_{1})}{n}\right)}^{n}=O\left(\frac{{(\mathrm{e}E\;)}^{n}}{{\varepsilon }^{2n}}\right),$$

where e denotes Euler’s number. Hence, we conclude that any energy-constrained state *ρ* can be approximated, up to trace-distance error *ϵ*, by its projection ${\rho }_{{M}_{1}}$ onto the subspace ${{\mathcal{H}}}_{{M}_{1}}$, which has a finite dimension of $O\left({(\mathrm{e}E)}^{n}/{\varepsilon }^{2n}\right)$.

Now, let us analyse the effective rank of the energy-constrained state *ρ*. We say that *ρ* has effective rank *r* if it is *ε*-close to a state with rank *r*. Let us consider the spectral decomposition

25 $$\rho =\sum_{i=1}^{\infty }{p}_{i}^{\downarrow }{\psi }_{i},$$

where the eigenvalues ${({p}_{i}^{\downarrow })}_{i}$ are not increasing in *i*. To estimate the effective rank, let us choose an integer *r* such that

26 $$\sum_{i=r+1}^{\infty }{p}_{i}^{\downarrow }\le \varepsilon ,$$

which guarantees that the *r*-rank state ${\rho }^{(r)}\propto \sum_{i = 1}^{r}{p}_{i}^{\downarrow }{\psi }_{i}$ is *O*(*ε*)-close to *ρ*. The infinite-dimensional Schur–Horn theorem (Proposition 6.4 in ref. ^53^) implies that for any *r*-rank projector *Π*,

27 $$\sum_{i=r+1}^{\infty }{p}_{i}^{\downarrow }\le {\rm{Tr}}[({\mathbb{1}}-\varPi )\rho ].$$

Moreover, by setting *M*_2_ := ⌈*nE*/*ε*⌉, the projector ${\varPi }_{{M}_{2}}$ is an *O*((e*E*)^*n*^/*ϵ*^*n*^)-rank projector satisfying

28 $${\rm{Tr}}[({\mathbb{1}}-{\varPi }_{{M}_{2}})\rho ]\le \frac{{\rm{Tr}}[{\hat{N}}_{n}\rho ]}{{M}_{2}}\le \frac{nE}{M}\le \varepsilon ,$$

where we have employed the same inequalities used in equations (23) and (24). Hence, by setting $\varPi ={\varPi }_{{M}_{2}}$, we deduce that *ρ* is *ε*-close to a state *ρ*^(*r*)^ having rank

29 $$r=O\left({(\mathrm{e}E\;)}^{n}/{\varepsilon }^{n}\right).$$

Finally, by exploiting the gentle measurement lemma^52^ and triangle inequality, one can easily show that the projection of *ρ*^(*r*)^ onto ${{\mathcal{H}}}_{{M}_{1}}$ is still *O*(*ε*)-close to *ρ*. Consequently, we conclude that any energy-constrained state can be approximated, up to trace-distance error *ε*, by a *D*-dimensional state with rank *r* such that

30 $$\begin{aligned}D&=O\left({(\mathrm{e}E\;)}^{n}/{\varepsilon }^{2n}\right),\\ r&=O\left({(\mathrm{e}E\;)}^{n}/{\varepsilon }^{n}\right).\end{aligned}$$

Based on these observations, we devised a simple tomography algorithm for energy-constrained states. The first step involves performing the two-outcome measurement $({\varPi }_{{M}_{1}},{\mathbb{1}}-{\varPi }_{{M}_{1}})$ and discarding the post-outcome state associated with ${\mathbb{1}}-{\varPi }_{{M}_{1}}$. This step transforms the unknown state *ρ* into the state ${\rho }_{{M}_{1}}$ with high probability. The state ${\rho }_{{M}_{1}}$ has two key properties: (1) It resides in the finite-dimensional subspace ${{\mathcal{H}}}_{{M}_{1}}$ of dimension $D=O\left({(\mathrm{e}E)}^{n}/{\varepsilon }^{2n}\right)$. (2) It is *O*(*ε*)-close to a state residing in ${{\mathcal{H}}}_{{M}_{1}}$ with rank *r* = *O*((e*E*)^*n*^/*ϵ*^*n*^). The second step involves performing the tomography algorithm of ref. ^54^ designed for *D*-dimensional state with rank *r*, which has a sample complexity of *O*(*Dr*). Importantly, this algorithm remains effective even if the unknown state, which is promised to reside in a given *D*-dimensional Hilbert space, has rank strictly larger than *r*, as long as it is *O*(*ε*)-close to a *r*-rank state within the same Hilbert space^54^. We, thus, conclude that the sample complexity in the tomography of energy-constrained states is upper bounded by *O*(*Dr*) = *O*((e*E*)^2*n*^/*ϵ*^3*n*^). Analogously, by exploiting that the sample complexity in the tomography of *D*-dimensional pure states is *O*(*D*) (refs. ^1,12–14^), we can show that the sample complexity in the tomography of energy-constrained pure states is upper bounded by *O*(*D*) = *O*((e*E*)^*n*^/*ϵ*^2*n*^).

For completeness, let us mention that the proof of the lower bounds on the sample complexity in the tomography of energy-constrained states, as presented in Theorems 1 and 2, primarily relies on epsilon-net tools^55^, like the qudit systems tackled in refs. ^12,54^. Detailed proofs of these results can be found in Supplementary Information.

### Bounds on the trace distance between Gaussian states

In this section, we address the question: If we know with a certain precision the first moment and the covariance matrix of an unknown Gaussian state, what is the resulting trace-distance error that we make on the state?

Let us formalize the problem. Let us consider a Gaussian state *ρ*_1_ and assume that we have an approximation of its first moment **m**(*ρ*_1_) and an approximation of its covariance matrix *V*(*ρ*_1_). For example, these approximations may be retrieved through homodyne detection on many copies of *ρ*_1_. We can then consider the Gaussian state *ρ*_2_ with first moment and covariance matrix equal to such approximations: **m**(*ρ*_2_) and *V*(*ρ*_2_) are, thus, the approximations of **m**(*ρ*_1_) and *V*(*ρ*_1_), respectively. The errors incurred in these approximations are naturally measured by the norm distances ∥**m**(*ρ*_1_) − **m**(*ρ*_2_)∥ and ∥*V*(*ρ*_1_) − *V*(*ρ*_2_)∥, respectively, where ∥ ⋅ ∥ denotes some norm. Now, a natural question arises: given an error *ε* in the approximations of the first moment and covariance matrix, what is the error incurred in the approximation of *ρ*_1_? The most meaningful way to measure such an error is given by the trace distance *d*_tr_(*ρ*_1_, *ρ*_2_) (refs. ^9,10^). Hence, the question becomes the following. If it holds that

31 $$\begin{aligned}\| {\bf{m}}(\;{\rho }_{1})-{\bf{m}}(\;{\rho }_{2})\| &=O(\varepsilon ),\\ \| V(\;{\rho }_{1})-V(\;{\rho }_{2})\| &=O(\varepsilon ),\end{aligned}$$

what can we say about the trace distance *d*_tr_(*ρ*_1_, *ρ*_2_)? Thanks to Theorems 10 and 11, we can answer this question. The trace distance *d*_tr_(*ρ*_1_, *ρ*_2_) is at most $O(\sqrt{\varepsilon })$ and at least *Ω*(*ε*).

This motivates the problem of finding upper and lower bounds on the trace distance between Gaussian states in terms of the norm distance of their first moments and covariance matrices. Now, we present our bounds, which are technical tools of independent interest.

Theorem 10, proven in Supplementary Information, gives our upper bound on the trace distance between Gaussian states.

#### Theorem 10

(Upper bound on the distance between Gaussian states) *Let ρ*_*1*_ *and ρ*_*2*_ *be n-mode Gaussian states satisfying the energy constraint* ${\rm{Tr}}[{\hat{N}}_{n}{\rho }_{1}],{\rm{Tr}}[{\hat{N}}_{n}{\rho }_{2}]\le N$. *Then*,

32 $$\begin{aligned}{d}_{{\rm{tr}}}({\rho }_{1},{\rho }_{2})&\le f(N)\bigg(\|{\bf{m}}(\;{\rho }_{1})-{\bf{m}}(\;{\rho }_{2})\| +\sqrt{2}\sqrt{\|V(\;{\rho }_{1})-V(\;{\rho }_{2}){\|}_{1}}\bigg),\end{aligned}$$

*where* $f(N):=\frac{1}{\sqrt{2}}\big(\sqrt{N}+\sqrt{N+1}\big)$. *Here* $\|{\bf{m}}\|:=\sqrt{{{\bf{m}}}^{\top }{\bf{m}}}$ *and* ∥ ⋅ ∥_1_ *denote the Euclidean norm and the trace norm, respectively*.

The above theorem turns out to be crucial for proving the upper bound on the sample complexity in the tomography of Gaussian states provided in Theorem 4.

One might believe that proving Theorem 10 would be straightforward by bounding the trace distance using the closed formula for the fidelity between Gaussian states^56^. However, this approach turns out to be highly non-trivial due to the complexity of such a fidelity formula^56^, which makes it challenging to derive a bound based on the norm distance between the first moments and covariance matrices. Instead, our proof directly addresses the trace distance without relying on fidelity and involves a meticulous analysis based on the energy-constrained diamond norm^57^.

The following theorem, proven in Supplementary Information, establishes our lower bound on the trace distance between Gaussian states.

#### Theorem 11

(Lower bound on the distance between Gaussian states) *Let ρ*_*1*_ *and ρ*_*2*_ *be n-mode Gaussian states satisfying the energy constraint* ${\rm{Tr}}[{\hat{E}}_{n}{\rho }_{1}],{\rm{Tr}}[{\hat{E}}_{n}{\rho }_{2}]\le E$. *Then*,

33 $$\begin{aligned}{d}_{{\rm{tr}}}(\;{\rho }_{1},{\rho }_{2})&\ge\frac{1}{200}\min \left\{1,\frac{\parallel {\bf{m}}(\;{\rho }_{1})-{\bf{m}}(\;{\rho }_{2})\parallel }{\sqrt{4E+1}}\right\},\\ {d}_{{\rm{tr}}}(\;{\rho }_{1},{\rho }_{2})&\ge\frac{1}{200}\min \left\{1,\frac{\parallel V(\;{\rho }_{2})-V(\;{\rho }_{1}){\parallel }_{2}}{4E+1}\right\},\end{aligned}$$

*where* $\|{\bf{m}}\|:=\sqrt{{{\bf{m}}}^{\top }{\bf{m}}}$ *and* $\parallel V{\parallel }_{2}:=\sqrt{{\rm{Tr}}[{V}^{\top }V]}$ *denote the Euclidean norm and the Hilbert–Schmidt norm, respectively*.

The proof of this theorem relies heavily on state-of-the-art bounds recently established for Gaussian probability distributions^58^.

Theorems 10 and 11 allow us to answer the question posed at the beginning of this section. Indeed, these theorems imply that, if we know with error *ε* the first moment and the covariance matrix of an unknown Gaussian state, the resulting trace-distance error that we make on the state is at most $O(\sqrt{\varepsilon })$ and at least *Ω*(*ε*). In particular, this proves Theorem 3.

The trace-distance bound of Theorem 10 can be improved by assuming one of the Gaussian states to be pure, as we detail in the following theorem, which is proven in the Supplementary Information.

#### Theorem 12

(Improved bound for pure states) *Let ψ be a pure n-mode Gaussian state and let ρ be an n-mode (possibly non-Gaussian) state satisfying the energy constraints* ${\rm{Tr}}[\psi {\hat{E}}_{n}],{\rm{Tr}}[\rho {\hat{E}}_{n}]\le E$. *Then*

34 $${d}_{{\rm{tr}}}(\;\rho ,\psi )\le \sqrt{E}\sqrt{2\|{\bf{m}}(\;\rho )-{\bf{m}}(\psi ){\|}^{2}+\|V(\;\rho )-V(\psi ){\|}_{\infty }},$$

*where* $\|{\bf{m}}\|:=\sqrt{{{\bf{m}}}^{\top }{\bf{m}}}$ *and* ∥ ⋅ ∥_*∞*_ *denote the Euclidean norm and the operator norm, respectively*.

By exploiting this improved bound, we show in Supplementary Information that the tomography of pure Gaussian states can be achieved using *O*(*n*^5^*E*^3^/*ϵ*^4^) copies of the state. This represents an improvement over the mixed-state scenario considered in Theorem 4.

Moreover, the bound in Theorem 12 can be useful for quantum-state certification^15^, as we briefly detail now. Suppose one aims to prepare a pure Gaussian state *ψ* with known first moment and covariance matrix. In a noisy experimental set-up, however, an unknown state *ρ* is effectively prepared. By accurately estimating the first two moments of *ρ* (which can be done efficiently, as shown in Supplementary Information), one can estimate the right-hand side of equation (34), which provides an upper bound on the trace distance between the target state *ψ* and the noisy state *ρ*, thereby providing a measure of the precision of the quantum device. Consequently, the device can be adjusted to minimize the error in state preparation.

## Online content

Any methods, additional references, Nature Portfolio reporting summaries, source data, extended data, supplementary information, acknowledgements, peer review information; details of author contributions and competing interests; and statements of data and code availability are available at 10.1038/s41567-025-03086-2.

## Supplementary information

Supplementary Information Supplementary discussion, Figs. 1–4 and Tables 1–4.

## Data Availability

No data were generated or analysed for this article.
