# Supplementary material for: Learning quantum states of continuous-variable systems
Source: Nat Phys. 2025 Nov 26;21(12):2002–8. doi: 10.1038/s41567-025-03086-2 (PMC12695625; doi:10.1038/s41567-025-03086-2)
Supplement: Supplementary file 1 — Supplementary discussion, Figs. 1–4 and Tables 1–4. [file 41567_2025_3086_MOESM1_ESM.pdf]

nature physics

Supplementary information https://doi.org/10.1038/s41567-025-03086-2

Learning quantum states of continuous-variable systems

In the format provided by the authors and unedited

Supplemental material

CONTENTS

S1. Preliminaries 1
A. Notation and basics 1
B. Basics of statistical learning theory 3
C. Preliminaries on continuous variable systems 5
D. Preliminaries on quantum learning theory 10
E. Preliminaries on ε-nets 13
F. Our results on ε-nets 13

S2. Tomography of moment-constrained states 19
A. Sample complexity lower bounds 20
1. Lower bound for pure state tomography 21
2. Lower bound for mixed state tomography 24
B. Sample complexity upper bounds 25
1. Properties of moment-constrained states: approximation with finite-dimensional and finite-rank states 26
2. Related works on approximating moment-constrained states 29
3. Known optimal tomography algorithm for qudits 29
4. Tomography algorithm for moment-constrained mixed and pure states 31

S3. Tomography of bosonic Gaussian states 34
A. Bounds on the trace distance between Gaussian states 35
1. Upper bound in the general (possibly mixed-state) setting 36
2. Upper bound in the pure-state setting 39
3. Lower bound 41
B. Learning first moments and covariance matrices 43
C. Learning Gaussian states 47
1. Second moment of the energy of a Gaussian state 47
2. Tomography algorithm for mixed Gaussian states 48
3. Noise robustness of the algorithm 50
4. Improved tomography algorithm for pure Gaussian states 51

S4. Tomography of t-doped bosonic Gaussian states 53
A. t-doped Gaussian states and unitaries 54
B. Compression of non-Gaussianity 54
C. t-compressible Gaussian states 57
D. Tomography algorithm for t-compressible Gaussian states 58

References 67

S1. PRELIMINARIES

A. Notation and basics

Let $\mathbb{N}$ denote the set of natural numbers, and for each $n \in \mathbb{N}$, define $[n] := \{1, 2, \dots, n\}$. We also define $\mathbb{N}_+ := \mathbb{N} \setminus \{0\}$ and $\mathbb{R}_+$ as the set of positive real numbers. We introduce $\mathbb{R}^{n\times n}$ and $\mathbb{C}^{n\times n}$ as the sets of $n \times n$ real and complex matrices, respectively. The notation $\lceil x \rceil$ rounds $x \in \mathbb{R}$ up to the nearest integer, while $\lfloor x \rfloor$ rounds x down to the nearest integer. Given a vector $v \in \mathbb{C}^n$ and a scalar $p \in [1, \infty]$, the p-norm of v is denoted by $\|v\|_p$, defined as

$$\|v\|_p := \left(\sum_{i=1}^{n} |v_i|^p \right)^{\frac{1}{p}} . \tag{S1}$$

For any matrix $A \in \mathbb{C}^{n\times n}$, its Schatten p-norm is given by $\|A\|_p := \mathrm{Tr}\left((\sqrt{A^\dagger A})^p\right)^{\frac{1}{p}}$, which corresponds to the p-norm of the singular values of A. The trace norm and the Hilbert-Schmidt norm, instances of Schatten p-norms, are denoted $\|\cdot\|_1$ and $\|\cdot\|_2$, respectively. The infinity norm, $\|\cdot\|_\infty$, represents the maximum singular value and equals the limit of the Schatten p-norms as $p \to \infty$. The Hölder inequality,

$$|\mathrm{Tr}(A^\dagger B)| \leq \|A\|_p \|B\|_q, \tag{S2}$$

applies for $1 \leq p, q \leq \infty$ with $\frac{1}{p} + \frac{1}{q} = 1$. Moreover, for all matrices $A \in \mathbb{C}^{n\times n}$ and $1 \leq p \leq q$, it holds that $\|A\|_q \leq \|A\|_p$ and $\|A\|_p \leq \mathrm{rank}(A)^{\left(\frac{1}{p}-\frac{1}{q}\right)}\|A\|_q$. We use the bra-ket notation, where we denote a vector $v \in \mathbb{C}^d$ using the ket notation $|v\rangle$ and its adjoint using the bra notation $\langle v|$. We refer to a vector $|\psi\rangle \in \mathbb{C}^d$ as normalized state vector if $\|\,|\psi\rangle\,\|_2 = 1$.

Given a Hilbert space $\mathcal{H}$, we denote with $\mathcal{D}(\mathcal{H})$ the set of quantum states on $\mathcal{H}$, i.e., positive semi-definite operators with unit trace. The trace distance between two quantum states ρ and σ is defined by $\frac{1}{2}\|\rho - \sigma\|_1$. The von Neumann entropy of a quantum state ρ is given by $S(\rho) := -\mathrm{Tr}[\rho \log_2 \rho]$.

We denote with $O(n)$ the group of $n \times n$ orthogonal matrices, and with $\mathrm{U}(n)$ the group of $n \times n$ unitary matrices. $\mathrm{Sp}(2n)$ denotes the group of symplectic matrices over the real field, defined as

$$\mathrm{Sp}(2n) := \{S \in \mathbb{R}^{2n,2n} \,:\, S\Omega S^T = \Omega\}, \tag{S3}$$

where

$$\Omega_n := \bigoplus_{i=1}^{n} \begin{pmatrix} 0 & 1 \\ -1 & 0 \end{pmatrix}. \tag{S4}$$

We denote the Fourier transform $\mathscr{F}$ of a function $g : \mathbb{R}^{2n} \to \mathbb{R}$ as

$$\mathscr{F} g(\mathbf{r}) = \int_{\mathbf{r}' \in \mathbb{R}^{2n}} \mathrm{d}^{2n}\mathbf{r}'\, g(\mathbf{r}') e^{-i\mathbf{r}^\intercal \mathbf{r}'}. \tag{S5}$$

Here, $\mathbf{r}^\intercal$ denotes the transpose of $\mathbf{r}$. Consequently, the inverse Fourier transform $\mathscr{F}^{-1}$ of a function $g : \mathbb{R}^{2n} \to \mathbb{R}$ is given by

$$\mathscr{F}^{-1} g(\mathbf{r}) = \frac{1}{(2\pi)^{2n}} \int_{\mathbf{r}' \in \mathbb{R}^{2n}} \mathrm{d}^{2n}\mathbf{r}'\, g(\mathbf{r}') e^{i\mathbf{r}'^\intercal \mathbf{r}}. \tag{S6}$$

For any random variable Z and any convex function f, Jensen Inequality states that $f(\mathbb{E}[Z]) \leq \mathbb{E}[f(Z)]$. Furthermore, if f is concave, then the opposite inequality holds. For any concave real function f, any Hermitian matrix X with eigenvalues pertaining to the domain of f, and any positive semi-definite matrix ρ, it holds that

$$\mathrm{Tr}(\rho f(X)) \leq f(\mathrm{Tr}(\rho X)), \tag{S7}$$

which can be shown by expanding X in its eigendecomposition and using Jensen inequality.

We review some basic notions regarding the asymptotic notation:

- Big-O notation: For a function $f(n)$, if there exists a constant c and a specific input size n_0 such that $f(n) \leq c \cdot g(n)$ for all $n \geq n_0$, where $g(n)$ is a well-defined function, then we express it as $f(n) = O(g(n))$. This notation signifies the upper limit of how fast a function grows in relation to $g(n)$.
- Big-Omega notation: For a function $f(n)$, if there exists a constant c and a specific input size n_0 such that $f(n) \geq c \cdot g(n)$ for all $n \geq n_0$, where $g(n)$ is a well-defined function, then we express it as $f(n) = \Omega(g(n))$. This notation signifies the lower limit of how fast a function grows in relation to $g(n)$.
- Big-Theta notation: For a function $f(n)$, if $f(n) = O(g(n))$ and if $f(n) = \Omega(g(n))$, where $g(n)$ is a well-defined function, then we express it as $f(n) = \Theta(g(n))$.

A tilde over $\mathcal{O}(\cdot)$, i.e., $\tilde{\mathcal{O}}(\cdot)$, implies that we are neglecting n factors at the numerator or denominator (for us, n will always represent the number of modes or qubits). For example, $f(n) = \frac{2^n}{n}$ is $\tilde{\Theta}(2^n)$. Analogously for the other asymptotic functions.

Note: Throughout the supplementary material, we will use the asymptotic notation described above. However, in the main text, we opted to simplify the notation by just using $O(\cdot)$ to informally denote the "relevant asymptotic scaling." Specifically, when in the main text we wrote that $y = O(f(\varepsilon, n))$ for some function $f(\cdot)$ of ε and n, we informally meant that "y is of the order of $f(\varepsilon, n)$ when n is very large and ε is very small." Importantly, to ensure clarity and avoid ambiguity, we express every sample complexity bound using both asymptotic notation and explicit exact expressions.

B. Basics of statistical learning theory

We present here basic results of probability and statistical learning theory; for further details we refer to Ref. [**?**].

Lemma S1 (Union bound)**.** *Let $B_1, B_2, \dots, B_M$ be events in a probability space. The probability of their union is bounded by the sum of their individual probabilities,*

$$\Pr\left[\bigcup_{i=1}^{M} B_i\right] \leq \sum_{i=1}^{M} \Pr[B_i]\,. \tag{S8}$$

Lemma S2 (Markov inequality)**.** *Let X be a non-negative random variable and $a > 0$. Then, the probability that X is at least a is bounded by the expected value of X divided by a, i.e.*

$$\Pr[X \geq a] \leq \frac{\mathbb{E}[X]}{a}. \tag{S9}$$

Lemma S3 (Chernoff bound)**.** *Consider a set of independent and identically distributed binary random variables $\{Y_i\}_{i=1}^{N}$, taking values in $\{0,1\}$. Define $Y := \sum_{i=1}^{N} Y_i$ and $\mu_Y := \mathbb{E}[Y]$. For any $\alpha \in (0,1)$, the probability of Y being less than $(1-\alpha)$ times its expected value is exponentially bounded as*

$$\Pr\left[Y \leq (1-\alpha)\mu_Y\right] \leq \exp\!\left(-\frac{\alpha^2 \mu_Y}{2}\right). \tag{S10}$$

We are now going to define the median-of-means estimator [**? ? ?**]. Let $N', K \in \mathbb{N}$. Given $N = N'K$ samples $\{X_m\}_{m=1}^{N}$ of the random variable X, divide the samples into K disjoint bins $\{\mathcal{B}_l\}_{l=1}^{K}$. Specifically, for each $l \in [K]$, let $\mathcal{B}_l$ be a set containing the elements $\{X_{(l-1)N'+1}, \dots, X_{lN'}\}$, where X_i denotes the i-th ordered sample. For each bin $\mathcal{B}_l$, define $\tilde{x}_l$ as the arithmetic average, i.e.,

$$\tilde{x}_l := \frac{1}{N'} \sum_{X_m \in \mathcal{B}_l} X_m. \tag{S11}$$

The median-of-means estimator is then given by

$$\hat{\mu}(N', K) := \mathrm{median}(\tilde{x}_1, \tilde{x}_2, \dots, \tilde{x}_K). \tag{S12}$$

We now present a lemma which will be crucial in our subsequent analysis.

Lemma S4 (Medians of means [**? ?**])**.** *Let X be a random variable with variance σ^2. Suppose K independent sample means of size $N' \geq \frac{34\sigma^2}{\varepsilon^2}$ suffice to construct a median-of-means estimator $\hat{\mu}(N,K)$ that satisfies*

$$\Pr[|\hat{\mu}(N,K) - \mathbb{E}[X]| \geq \varepsilon] \leq 2\mathrm{e}^{-K/2}, \quad \forall \varepsilon > 0\,. \tag{S13}$$

As a consequence, $N \geq 68 \log\!\left(\frac{2}{\delta}\right) \frac{\sigma^2}{\varepsilon^2}$ samples of X suffice to construct a median-of-means estimator $\hat{\mu}$ which satisfies

$$\Pr[|\hat{\mu} - \mathbb{E}[X]| \geq \varepsilon] \leq \delta \quad \forall \varepsilon > 0\,. \tag{S14}$$

We also mention the following (standard) fact that is useful in amplifying the probability of success of an algorithm.

Lemma S5 (Enhancing the probability of success)**.** *Let $\mathcal{A}$ be an algorithm with a success probability of at least $p_{\mathrm{succ}} \in (0,1]$. Let $\delta > 0$ and $N' \in \mathbb{N}$. If we execute $\mathcal{A}$ a total of m times, with*

$$m \geq \left\lceil \frac{3}{2p_{\mathrm{succ}}} N' + \frac{18}{p_{\mathrm{succ}}} \log\!\left(\frac{1}{\delta}\right) \right\rceil, \tag{S15}$$

then $\mathcal{A}$ will achieve success at least N' times with a probability of at least $1-\delta$.

Proof. Consider the binary random variables $\{X_i\}_{i=1}^{m}$ defined as

$$X_i := \begin{cases} 1 & \text{if } \mathcal{A} \text{ succeeds,} \\ 0 & \text{if } \mathcal{A} \text{ fails.} \end{cases} \tag{S16}$$

Let $\hat{X} := \sum_{i=1}^{m} X_i$, and note that $\mathbb{E}[\hat{X}] = mp_{\text{succ}}$. Our goal is to upper bound the probability that $\mathcal{A}$ succeeds fewer than N' times. Define $\alpha := 1 - \frac{N'}{mp_{\text{succ}}}$. Using that $m \geq \frac{3}{2p_{\text{succ}}} N'$, we ensure that $\alpha \in \left(\frac{1}{3}, 1\right)$. Applying the Chernoff bound in Lemma S3, we get:

$$\begin{aligned}
\Pr\left(\hat{X} \leq N'\right) &= \Pr\left(\hat{X} \leq (1-\alpha)\mathbb{E}[\hat{X}]\right) \\
&\leq \exp\left(-\frac{\alpha^2 \mathbb{E}[\hat{X}]}{2}\right) \\
&= \exp\left(-\frac{\alpha^2}{2} p_{\text{succ}} m\right) \\
&\leq \exp\left(-\frac{p_{\text{succ}}}{18} m\right) \\
&\leq \delta,
\end{aligned} \tag{S17}$$

where in the second inequality we have used that $\alpha \geq \frac{1}{3}$, while in the last inequality we have used

$$m \geq \frac{18}{p_{\text{succ}}} \log\left(\frac{1}{\delta}\right). \tag{S18}$$

□

We present a lemma that enhances the probability of success of a learning algorithm. Although the proof follows standard steps similar to those in Ref. [**?**] (Proposition 2.4), we provide it here with precise constants, which were not explicitly stated in Ref. [**?**].

Lemma S6 (Enhancing the probability of success of an algorithm for learning objects in a metric space)**.** *Let* $p_{\text{succ}} \in (\frac{1}{2}, 1]$ *and* $\varepsilon > 0$. *Let* $\mathcal{A}$ *be an algorithm for learning unknown objects in a metric space with distance* $d(\cdot,\cdot)$. *Assume that for any (unknown) input object* ρ *the algorithm* $\mathcal{A}$ *outputs an object* $\tilde{\rho}$ *such that*

$$\Pr\left[d(\tilde{\rho}, \rho) \leq \varepsilon\right] \geq p_{\text{succ}}\,. \tag{S19}$$

Then for any $\delta \in (0, 1]$ *there exists an algorithm* $\mathcal{A}'$, *which executes* $\mathcal{A}$ *a total of* m *times with*

$$m := \left\lceil \frac{2}{\left(1 - \frac{1}{2p_{\text{succ}}}\right)^2 p_{\text{succ}}} \log\left(\frac{1}{\delta}\right) \right\rceil, \tag{S20}$$

such that for any (unknown) input object ρ *it outputs an object* $\tilde{\rho}$ *such that*

$$\Pr\left[d(\tilde{\rho}, \rho) \leq 3\varepsilon\right] \geq 1 - \delta\,. \tag{S21}$$

Proof. The algorithm $\mathcal{A}'$, by executing $\mathcal{A}$ a total of m times, produces m random objects $\tilde{\rho}_1, \ldots, \tilde{\rho}_m$ such that each $\tilde{\rho}_i$ satisfies $\Pr\left[d(\tilde{\rho}_i, \rho) \leq \varepsilon\right] \geq p_{\text{succ}}$. Let us show that the probability $\bar{P}$ that there are more than $\frac{m}{2}$ objects in the set $\mathcal{S} := \{\tilde{\rho}_1, \ldots, \tilde{\rho}_m\}$ which are close at most ε to ρ is not smaller than $1 - \delta$. Consider the binary random variables $\{X_i\}_{i=1}^m$ defined as

$$X_i := \begin{cases} 1 & \text{if } d(\tilde{\rho}_i, \rho) \leq \varepsilon, \\ 0 & \text{otherwise.} \end{cases} \tag{S22}$$

Let $\hat{X} := \sum_{i=1}^{m} X_i$, and note that $\mathbb{E}[\hat{X}] = mp_{\text{succ}}$. Using that $p_{\text{succ}} \in (\frac{1}{2}, 1)$, we ensure that the quantity $\alpha := 1 - \frac{1}{2p_{\text{succ}}}$ satisfies

$\alpha \in (0,1)$. Then, by applying the Chernoff bound in Lemma S3, we have that

$$\begin{aligned}\bar{P} &= \Pr\left(\hat{X} > \frac{m}{2}\right) \\ &= 1 - \Pr\left(\hat{X} \le \frac{m}{2}\right) \\ &= 1 - \Pr\left(\hat{X} \le (1-\alpha)\mathbb{E}[\hat{X}]\right) \\ &\ge 1 - \exp\left(-\frac{\alpha^2 \mathbb{E}[\hat{X}]}{2}\right) \\ &= 1 - \exp\left(-\frac{\left(1-\frac{1}{2p_{\text{succ}}}\right)^2 p_{\text{succ}}}{2} m\right) \\ &\ge 1-\delta\,,\end{aligned} \tag{S23}$$

where in the last inequality we have used that

$$m \ge \frac{2}{\left(1-\frac{1}{2p_{\text{succ}}}\right)^2 p_{\text{succ}}} \log\left(\frac{1}{\delta}\right). \tag{S24}$$

From now on, let us assume that there are more than $\frac{m}{2}$ objects in $\mathscr{S}$ which are close at most ε to ρ (which is an event that happens with probability at least $1-\delta$, as proved above). Then, by the triangle inequality, there are more than $\frac{m}{2}$ objects in $\mathscr{S}$ which are close at most 2ε to each other.

The algorithm $\mathscr{A}'$ is as follows: First, it computes the distance between any two objects in $\mathscr{S}$ and, second, it outputs an object $\tilde{\rho} \in \mathscr{S}$ that satisfies the property of being close at most 2ε to more than $\frac{m}{2}$ objects in $\mathscr{S}$.

Let us show that the output $\tilde{\rho}$ satisfies $d(\tilde{\rho},\rho) \le 3\varepsilon$. Let $\mathscr{S}_{\tilde{\rho}}$ be the set of objects in $\mathscr{S}$ that are close at most 2ε to $\tilde{\rho}$. Since $|\mathscr{S}_{\tilde{\rho}}| > \frac{m}{2}$, $|\mathscr{S}| = m$, and $\mathscr{S}$ contains more than $\frac{m}{2}$ objects which are close at most ε to ρ, then there exists an object $\sigma \in \mathscr{S}_{\tilde{\rho}}$ with $d(\sigma,\rho) \le \varepsilon$. Then, by the triangle inequality, we conclude that $d(\tilde{\rho},\rho) \le 3\varepsilon$. □

The right-hand side of (S20) diverges as $p_{\text{succ}} \to \frac{1}{2}$. This is consistent with the simple fact that, in general, the probability of success can not be arbitrarily enhanced when $p_{\text{succ}} \le \frac{1}{2}$. Indeed, in the latter case, we could consider an algorithm $\mathscr{A}$ such that for any unknown input ρ it outputs: the exact true object ρ with probability p_{succ}; an object σ_ρ, which is very distant from ρ, with the same probability p_{succ}; and a fixed object τ (independent of ρ) with probability $1-2p_{\text{succ}}$. Hence, since the objects ρ and σ_ρ are statistically indistinguishable, there is no way to arbitrarily enhance the probability of success of $\mathscr{A}$, even with infinite executions of it.

C. Preliminaries on continuous variable systems

In this section, we provide a concise overview of quantum information with *continuous variable* (CV) systems; for further details, we refer to Refs. [**? ? ?**]. Let us start by offering an intuitive explanation of what a continuous-variable system is from a quantum information perspective. In quantum information, a familiar concept is that of a *qudit* — a quantum system with a d-dimensional Hilbert space. Specifically, the Hilbert space of a qu$d$it is defined as the span of the computational basis state vectors $\{|0\rangle, |1\rangle, \ldots, |d-1\rangle\}$

$$\mathcal{H}_d \coloneqq \text{Span}\{|0\rangle, |1\rangle, \ldots, |d-1\rangle\}\,. \tag{S25}$$

In continuous-variable systems, the notion of a qu$d$it is replaced by the concept of a *mode*. A mode can be thought of as a qu$d$it in the limit $d \to \infty$, that is, a quantum system with an infinite-dimensional Hilbert space of the form

$$\mathcal{H}_\infty \coloneqq \text{Span}\left\{|n\rangle :\ n \in \mathbb{N}\right\}\,. \tag{S26}$$

By definition, a *continuous-variable system* is a quantum system that consists of n modes and is described by the tensor product space $\mathcal{H}_\infty^{\otimes n}$. In this sense, a continuous variable system of n modes can be intuitively viewed as n qu$d$its with $d=\infty$. The computational basis state vector $|n\rangle$ of the Hilbert space of a single mode is called the *n-th Fock state*, and in quantum optics it represents the quantum state of light with n photons. In particular, the state vector $|0\rangle$ is the *vacuum*, i.e. the state with zero photons.

The *annihilation operator*, denoted as a, is defined in the Fock basis as

$$a = \sum_{m=0}^{\infty} \sqrt{m!}\, |m-1\rangle\langle m| \ . \tag{S27}$$

Its adjoint $a^\dagger$ is known as the *creation operator*. These two operators satisfy *canonical commutation relation*

$$[a, a^\dagger] = \mathbb{1}\,, \tag{S28}$$

as they are commonly stated, where $[A, B] \coloneqq AB - BA$ denotes the commutator and $\mathbb{1}$ is the identity operator. Using the orthonormality of the Fock states, one can show that the m-th Fock state can be obtained by applying the creation operator m times to the vacuum state vector

$$|m\rangle \coloneqq \frac{(a^\dagger)^m}{\sqrt{m!}}\, |0\rangle \ . \tag{S29}$$

The operator $a^\dagger a$ is known as the *single-mode photon number operator*, and it is diagonal in the Fock basis

$$a^\dagger a = \sum_{m=0}^{\infty} m\, |m\rangle\langle m| \ . \tag{S30}$$

The *position* and *momentum* operators, denoted by $\hat{x}$ and $\hat{p}$ respectively, can be defined in terms of the annihilation and creation operators as

$$\hat{x} \coloneqq \frac{a + a^\dagger}{\sqrt{2}}\,,\ \hat{p} \coloneqq \frac{a - a^\dagger}{\sqrt{2}i} \ . \tag{S31}$$

In terms of these operators, the canonical commutation relation takes the form $[\hat{x}, \hat{p}] = i\mathbb{1}$. When expressing the canonical commutation relations, unbounded operators can be avoided by rigorously capturing them in terms of the Weyl relations.

From a mathematical standpoint, the Hilbert space associated with a single mode is identified with the space $L^2(\mathbb{R})$ [**?**] which comprises all square-integrable complex-valued functions over $\mathbb{R}$. More generally, the Hilbert space of an n-mode continuous-variable system is given by $L^2(\mathbb{R}^n)$ — that is, the space of square-integrable, complex-valued functions over $\mathbb{R}^n$. The set of quantum states of such a system is denoted by $\mathcal{D}(L^2(\mathbb{R}^n))$. Notably, it holds that $L^2(\mathbb{R}^n) = L^2(\mathbb{R})^{\otimes n}$, so the Hilbert space of an n-mode system can be naturally understood as the n-fold tensor product of the Hilbert space of a single mode. On this Hilbert space, one can define the position and momentum operators of the j-th mode, denoted respectively by $\hat{x}_j$ and $\hat{p}_j$ [**?**]. From this, one can define the annihilation operator of the j-th mode, as

$$a_j \coloneqq \frac{\hat{x}_j + i\hat{p}_j}{\sqrt{2}} \ . \tag{S32}$$

By introducing the quadrature vector

$$\hat{\mathbf{R}} \coloneqq (\hat{x}_1, \hat{p}_1, \dots, \hat{x}_n, \hat{p}_n)^\intercal = (\hat{R}_1, \hat{R}_2, \dots, \hat{R}_{2n-1}, \hat{R}_{2n})^\intercal \tag{S33}$$

the canonical commutation relations can be expressed as

$$[\hat{R}_k, \hat{R}_l] = i\,(\Omega_n)_{k,l}\hat{\mathbb{1}} \qquad \forall\, k, l \in [2n]\,, \tag{S34}$$

where

$$\Omega_n \coloneqq \bigoplus_{i=1}^{n} \begin{pmatrix} 0 & 1 \\ -1 & 0 \end{pmatrix} = \mathbb{1}_n \otimes \begin{pmatrix} 0 & 1 \\ -1 & 0 \end{pmatrix} \tag{S35}$$

is the n-mode symplectic form, $\mathbb{1}_n$ is the $n \times n$ identity matrix, and $\hat{\mathbb{1}}$ is the identity operator over $L^2(\mathbb{R}^n)$. The relation in (S34) is usually expressed in the continuous variable literature [**?**] in a compact form as

$$[\hat{\mathbf{R}}, \hat{\mathbf{R}}^\intercal] = i\,\Omega_n\hat{\mathbb{1}}\,, \tag{S36}$$

which we denote as 'vectorial notation'. The energy operator $\hat{E}_n$ is defined as

$$\hat{E}_n \coloneqq \frac{1}{2}\hat{\mathbf{R}}^\intercal\hat{\mathbf{R}} = \sum_{j=1}^{n}\left(\frac{\hat{x}_j^2}{2} + \frac{\hat{p}_j^2}{2}\right) = \sum_{j=1}^{n}\left(a_j^\dagger a_j + \frac{\hat{\mathbb{1}}}{2}\right) = \hat{N}_n + \frac{n}{2}\hat{\mathbb{1}}\,, \tag{S37}$$

where $\hat{N}_n := \sum_{j=1}^{n} a_j^\dagger a_j$ is the photon number operator. The characteristic function

$$\chi_\rho : \mathbb{R}^{2n} \to \mathbb{C} \tag{S38}$$

of an n-mode state $\rho \in \mathcal{D}\big(L^2(\mathbb{R}^n)\big)$ is defined as $\chi_\rho(\mathbf{r}) := \mathrm{Tr}[\rho \hat{D}_\mathbf{r}]$, where for all $\mathbf{r} \in \mathbb{R}^{2n}$ the displacement operator $\hat{D}_\mathbf{r}$ is given by $\hat{D}_\mathbf{r} := e^{-i\mathbf{r}^\intercal \Omega_n \hat{\mathbf{R}}}$. Any state ρ can be written in terms of its characteristic function as [**?**]

$$\rho = \int_{\mathbb{R}^{2n}} \frac{\mathrm{d}^{2n}\mathbf{r}}{(2\pi)^n} \chi_\rho(\mathbf{r}) \hat{D}_\mathbf{r}^\dagger \,, \tag{S39}$$

and hence quantum states and characteristic functions are in one-to-one correspondence.

The Wigner function $W_\rho : \mathbb{R}^{2n} \to \mathbb{R}$ of an n-mode state ρ is defined as the inverse Fourier transform $\mathscr{F}^{-1}$ of the characteristic function χ_ρ, evaluated at $\Omega_n \mathbf{r}$

$$W_\rho(\mathbf{r}) = \mathscr{F}^{-1}\chi_\rho(\Omega_n \mathbf{r}) = \frac{1}{(2\pi)^{2n}} \int_{\mathbf{r}' \in \mathbb{R}^{2n}} \mathrm{d}^{2n}\mathbf{r}' \, \chi_\rho(\mathbf{r}') e^{i\mathbf{r}'^\intercal \Omega_n \mathbf{r}}. \tag{S40}$$

Consequently, the characteristic function χ_ρ can be expressed as the Fourier transform

$$\chi_\rho(\mathbf{r}) = \mathscr{F} W_\rho(\Omega_n \mathbf{r}) = \int_{\mathbf{r}' \in \mathbb{R}^{2n}} \mathrm{d}^{2n}\mathbf{r}' \, W_\rho(\mathbf{r}') e^{-i\mathbf{r}^\intercal \Omega_n \mathbf{r}'}, \tag{S41}$$

evaluated at $\Omega_n \mathbf{r}$, of the Wigner function W_ρ. Moreover, the Husimi function $Q_\rho(\mathbf{r}) : \mathbb{R}^{2n} \to \mathbb{R}$ of an n-mode state ρ is defined as

$$Q_\rho(\mathbf{r}) := \frac{1}{(2\pi)^n} \langle \mathbf{r} | \rho | \mathbf{r} \rangle \,, \tag{S42}$$

where $|\mathbf{r}\rangle := \hat{D}_\mathbf{r} |0\rangle$ is a coherent state vector. It turns out that the Fourier transform of the Husimi function, evaluated at $\Omega_n \mathbf{r}$, is related to the characteristic function as [**?**]

$$\int_{\mathbf{r}' \in \mathbb{R}^{2n}} \mathrm{d}^{2n}\mathbf{r}' \, Q_\rho(\mathbf{r}') e^{-i\mathbf{r}^\intercal \Omega_n \mathbf{r}'} = e^{-\frac{1}{4}\mathbf{r}^\intercal \mathbf{r}} \chi_\rho(\mathbf{r}) \,. \tag{S43}$$

The Husimi function $Q_\rho(\mathbf{r})$ is a useful quantity since it is the probability distribution of the outcome $\mathbf{r} \in \mathbb{R}^{2n}$ of a (experimentally feasible) measurement — known as *heterodyne measurement* — performed on the state ρ [**?**]. The first moment of a quantum state ρ is defined as $\mathbf{m}(\rho) := (m_1(\rho), \ldots, m_n(\rho))^\intercal$, where

$$m_k(\rho) := \mathrm{Tr}\Big[\hat{R}_k \, \rho\Big] \,, \tag{S44}$$

for each $k \in [n]$, or in its vectorial notation as $\mathbf{m}(\rho) = \mathrm{Tr}\Big[\hat{\mathbf{R}} \, \rho\Big]$. Additionally, the covariance matrix of ρ is defined by the matrix $V(\rho)$ with elements

$$[V(\rho)]_{k,l} := \mathrm{Tr}\Big[\Big\{\hat{R}_k - m_k(\rho)\mathbb{1}, \hat{R}_l - m_l(\rho)\mathbb{1}\Big\} \rho\Big] = \mathrm{Tr}\Big[\Big\{\hat{R}_k, \hat{R}_l\Big\} \rho\Big] - 2m_k(\rho)m_l(\rho) \,, \tag{S45}$$

for each $k, l \in [2n]$, where $\{\hat{A}, \hat{B}\} := \hat{A}\hat{B} + \hat{B}\hat{A}$ is the anti-commutator. In its vectorial notation this reads as

$$V(\rho) = \mathrm{Tr}\Big[\Big\{(\hat{\mathbf{R}} - \mathbf{m}(\rho)), (\hat{\mathbf{R}} - \mathbf{m}(\rho))^\intercal\Big\} \rho\Big] = \mathrm{Tr}\Big[\Big\{\hat{\mathbf{R}}, \hat{\mathbf{R}}^\intercal\Big\} \rho\Big] - 2\mathbf{m}(\rho)\mathbf{m}(\rho)^\intercal \,. \tag{S46}$$

Any covariance matrix $V(\rho)$ satisfies the inequality

$$V(\rho) + i\Omega_n \geq 0 \,, \tag{S47}$$

known as *uncertainty relation*. As a consequence, since Ω_n is skew-symmetric, any covariance matrix $V(\rho)$ is positive semi-definite on $\mathbb{R}^{2n}$. Conversely, for any symmetric $W \in \mathbb{R}^{2n,2n}$ such that $W + i\Omega_n \geq 0$ there exists an n-mode (Gaussian) state ρ with covariance matrix $V(\rho) = W$ [**?**].

Definition S7 (Gaussian state)**.** *An n-mode state ρ is said to be a Gaussian state if it can be written as a Gibbs state of a quadratic Hamiltonian $\hat{H}$ in the quadrature operators $\{\hat{R}_i\}_{i\in[2n]}$, that is,*

$$\hat{H} := \frac{1}{2}(\hat{\mathbf{R}} - \mathbf{m})^\intercal H(\hat{\mathbf{R}} - \mathbf{m}) \tag{S48}$$

for some symmetric positive-definite matrix $H \in \mathbb{R}^{2n,2n}$ and some vector $\mathbf{m} \in \mathbb{R}^{2n}$. The Gibbs states associated with the Hamiltonian $\hat{H}$ are given by

$$\rho = \left(\frac{e^{-\beta\hat{H}}}{\mathrm{Tr}[e^{-\beta\hat{H}}]}\right)_{\beta\in[0,\infty]}, \tag{S49}$$

where the parameter β is called the 'inverse temperature'.

Remark S8. *Definition S7 includes also the pathological cases where both β and certain terms of H diverge (e.g., this is the case for tensor products between pure Gaussian states and mixed Gaussian states). To formalise this mathematically, one can define the set of Gaussian states as the closure, with respect to the trace norm, of the set of Gibbs states of quadratic Hamiltonians [**?**].*

The characteristic function of a Gaussian state ρ is the Fourier transform of a Gaussian probability distribution, evaluated at $\Omega_n\mathbf{r}$, which can be written in terms of $\mathbf{m}(\rho)$ and $V(\rho)$ as [**?**]

$$\chi_\rho(\mathbf{r}) = \exp\left(-\frac{1}{4}(\Omega_n\mathbf{r})^\intercal V(\rho)\Omega_n\mathbf{r} + i(\Omega_n\mathbf{r})^\intercal\mathbf{m}(\rho)\right). \tag{S50}$$

Consequently, the Wigner function of a Gaussian state can be expressed as a Gaussian probability distribution as

$$W_\rho(\mathbf{r}) = \frac{e^{-(\mathbf{r}-\mathbf{m}(\rho))^\intercal[V(\rho)]^{-1}(\mathbf{r}-\mathbf{m}(\rho))}}{\pi^n\sqrt{\det[V(\rho)]}}. \tag{S51}$$

Note that a Gaussian probability distribution with first moment $\mathbf{m}$ and covariance matrix V is defined as

$$\mathcal{N}[\mathbf{m}, V](\mathbf{r}) := \frac{e^{-\frac{1}{2}(\mathbf{r}-\mathbf{m})^\intercal V^{-1}(\mathbf{r}-\mathbf{m})}}{(2\pi)^n\sqrt{\det V}}. \tag{S52}$$

Hence, using such notation, the Wigner function of a Gaussian state ρ is a Gaussian probability distribution with first moment $\mathbf{m}(\rho)$ and covariance matrix $V(\rho)/2$, that is,

$$W_\rho(\mathbf{r}) = \mathcal{N}\left[\mathbf{m}(\rho), \frac{V(\rho)}{2}\right](\mathbf{r}). \tag{S53}$$

Hence, (S43) and (S50) imply that the Husimi function of a Gaussian state ρ is a Gaussian probability distribution with first moment $\mathbf{m}(\rho)$ and covariance matrix $(V(\rho) + \mathbb{1})/2$, that is,

$$Q_\rho(\mathbf{r}) = \mathcal{N}\left[\mathbf{m}(\rho), \frac{V(\rho)+\mathbb{1}}{2}\right](\mathbf{r}). \tag{S54}$$

Since any quantum state is uniquely identified by its characteristic function, it follows from (S50) that any Gaussian state is uniquely identified by its first moment and covariance matrix. An example of Gaussian state is the single-mode thermal state

$$\tau_\nu := \frac{1}{\nu+1}\sum_{n=0}^{\infty}\left(\frac{\nu}{\nu+1}\right)^n |n\rangle\!\langle n|, \tag{S55}$$

where $\nu \geq 0$. It holds that $\mathrm{Tr}[a^\dagger a\,\tau_\nu] = \nu$, thus ν is the mean photon number of τ_ν. The first moment and the covariance matrix of τ_ν satisfy

$$\begin{aligned}\mathbf{m}(\tau_\nu) &= (0,0)^\intercal, \\ V(\tau_\nu) &= (2\nu+1)\mathbb{1}_2.\end{aligned} \tag{S56}$$

It is worth noting that the thermal state with $\nu = 0$ is the vacuum state vector, $\tau_0 = |0\rangle\!\langle 0|$. Thermal states are important since they maximise the von Neumann entropy among all states with a fixed mean photon number, as established by Lemma S9 [**?**].

Lemma S9 (Extremality of thermal states)**.** *For all $\nu > 0$, the maximum von Neumann entropy among all n-mode states with a given mean photon number ν is achieved by a thermal state $\tau_{\nu/n}^{\otimes n}$, i.e.,*

$$\max\left\{S(\rho):\ \rho \in \mathcal{D}(L^2(\mathbb{R}^n)),\ \operatorname{Tr}\left[\rho\sum_{i=1}^{n} a_i^\dagger a_i\right] \le \nu\right\} = S\left(\tau_{\nu/n}^{\otimes n}\right) = ng\left(\frac{\nu}{n}\right)\,, \tag{S57}$$

where

$$g(\nu) \coloneqq (\nu+1)\log_2(\nu+1) - \nu\log_2\nu \tag{S58}$$

is a monotone increasing function called the bosonic entropy.

In the setting of continuous variable systems, symplectic matrices play a crucial role. Recall that a matrix $S \in \mathbb{R}^{2n,2n}$ is *symplectic* matrix if and only if $S\Omega_n S^\intercal = \Omega_n$ and that the group of symplectic matrices is denoted by $\mathrm{Sp}(2n)$. Fixed a symplectic matrix $S \in \mathrm{Sp}(2n)$, one can define a suitable n-mode unitary U_S — dubbed *symplectic unitary* or the *metaplectic representation* of S — such that, for each $k \in [2n]$ it holds

$$U_S^\dagger \hat{R}_k U_S = \sum_{k,l}^{2n} S_{k,l}\hat{R}_l. \tag{S59}$$

In the vectorial notation, we can write this as

$$U_S^\dagger \hat{\mathbf{R}} U_S = S\hat{\mathbf{R}}\,. \tag{S60}$$

More explicitly, the symplecitic unitary U_S is defined in terms of the symplectic matrix S as follows [**?**]. Any symplectic matrix S can be written as $S = S_1S_2$, where $S_1 \coloneqq e^{\Omega_n H_1}$ and $S_2 \coloneqq e^{\Omega_n H_2}$ for some symmetric matrices H_1, H_2 [**?**]. Then, U_S is defined as $U_S \coloneqq U_{S_1}U_{S_2}$, where

$$\begin{aligned} U_{S_1} &\coloneqq e^{-\frac{i}{2}\hat{\mathbf{R}}^\intercal H_1 \hat{\mathbf{R}}}\,, \\ U_{S_2} &\coloneqq e^{-\frac{i}{2}\hat{\mathbf{R}}^\intercal H_2 \hat{\mathbf{R}}}\,. \end{aligned} \tag{S61}$$

In particular, (S60) implies that

$$\begin{aligned} \mathbf{m}(U_S\rho U_S^\dagger) &= S\mathbf{m}(\rho)\,, \\ V(U_S\rho U_S^\dagger) &= SV(\rho)S^\intercal\,. \end{aligned} \tag{S62}$$

Given two symplectic matrices S_1 and S_2, it holds that $U_{S_1}U_{S_2} = U_{S_1S_2}$. In addition, the displacement operator $\hat{D}_\mathbf{r}$ transforms the quadrature vector as

$$\hat{D}_\mathbf{r}\hat{\mathbf{R}}\hat{D}_\mathbf{r}^\dagger = \hat{\mathbf{R}} + \mathbf{r}\hat{\mathbb{1}}, \tag{S63}$$

which implies that

$$\begin{aligned} \mathbf{m}\left(\hat{D}_\mathbf{r}\rho\hat{D}_\mathbf{r}^\dagger\right) &= \mathbf{m}(\rho) + \mathbf{r}\,, \\ V\left(\hat{D}_\mathbf{r}\rho\hat{D}_\mathbf{r}^\dagger\right) &= V(\rho)\,. \end{aligned} \tag{S64}$$

Notably, any covariance matrix $V(\rho)$ of an n-mode state ρ satisfies the following decomposition, known as the Williamson decomposition [**?**]: there exists a symplectic matrix $S \in \mathrm{Sp}(2n)$ and n real numbers $d_1, d_2, \dots, d_n \ge 1$, called the symplectic eigenvalues of $V(\rho)$, such that

$$V(\rho) = SDS^\intercal\,, \tag{S65}$$

where

$$D = \bigoplus_{j=1}^{n}\left(d_j\begin{pmatrix}1 & 0\\ 0 & 1\end{pmatrix}\right) = \operatorname{diag}\left(d_1, d_1, d_2, d_2, \dots, d_n, d_n\right)\,. \tag{S66}$$

In addition, if ρ is Gaussian, it is possible to show that the above Williamson decomposition of the covariance matrix leads to the decomposition

$$\rho = \hat{D}_{\mathbf{m}(\rho)} U_S \left(\tau_{\nu_1} \otimes \tau_{\nu_2} \otimes \dots \tau_{\nu_n}\right) U_S^\dagger \hat{D}_{\mathbf{m}(\rho)}^\dagger \tag{S67}$$

for the state ρ, where $\{\tau_{\nu_k}\}_{k=1}^n$ are thermal states defined in Eq. (S55), and the mean photon numbers $\nu_1, \nu_2, \dots, \nu_n$ are defined in terms of the symplectic eigenvalues of $V(\rho)$ via the relation $\nu_i := (d_i - 1)/2$ for all $i \in [n]$. In other words, any Gaussian state is unitarily equivalent — through displacement and symplectic unitaries — to a multi-mode thermal state. In particular, any pure Gaussian state vector $|\phi\rangle$ can be written as

$$|\phi\rangle = \hat{D}_{\mathbf{r}} U_S |0\rangle^{\otimes n} . \tag{S68}$$

Moreover, Gaussian unitaries can be defined as follows.

Definition S10 (Gaussian unitary)**.** *An n-mode unitary is said to be Gaussian if it is the composition of unitaries generated by quadratic Hamiltonians. A Hamiltonian $\hat{H}$ is said to be quadratic if it can be written as $\hat{H} := \frac{1}{2}(\hat{\mathbf{R}} - \mathbf{m})^\intercal H (\hat{\mathbf{R}} - \mathbf{m})$, where $\mathbf{r} \in \mathbb{R}^{2n}$ and $H \in \mathbb{R}^{2n,2n}$ is a symmetric matrix.*

In particular, for any n-mode Gaussian unitary U there exists a symplectic matrix $S \in \mathrm{Sp}(2n)$ and a vector $\mathbf{r} \in \mathbb{R}^{2n}$ such that $U = \hat{D}_{\mathbf{r}} U_S$, where U_S is the symplectic unitary associated with S. Conversely, for any $S \in \mathrm{Sp}(2n)$ and $\mathbf{r} \in \mathbb{R}^{2n}$, the unitary $\hat{D}_{\mathbf{r}} U_S$ is a Gaussian unitary. In other words, Gaussian unitaries are the composition of displacement and symplectic unitaries.

Notably, any symplectic matrix $S \in \mathrm{Sp}(2n)$ can be written in the so-called Euler (or Bloch-Messiah) decomposition as follows: there exist symplectic orthogonal matrices $O_1, O_2 \in \mathrm{O}(2n) \cap \mathrm{Sp}(2n)$ and real numbers $z_1, z_1, \dots, z_n \geq 1$ such that

$$S = O_1 Z O_2 \tag{S69}$$

with

$$Z := \bigoplus_{j=1}^n \begin{pmatrix} z_j & 0 \\ 0 & z_j^{-1} \end{pmatrix} . \tag{S70}$$

Symplectic unitaries associated with symplectic matrices of the form of Z are said to be *squeezing* unitaries. Moreover, symplectic unitaries associated with symplectic orthogonal matrices are said to be *passive*. Importantly, passive unitaries preserve the energy operator, meaning that given $O \in \mathrm{O}(2n) \cap \mathrm{Sp}(2n)$ then

$$U_O^\dagger \hat{E}_n U_O = \hat{E}_n . \tag{S71}$$

Moreover, note that the Euler decomposition in (S69) implies that any Gaussian unitary can be written as a composition of passive unitaries and squeezing unitary, i.e., $U_S = U_{O_1} U_Z U_{O_2}$.

Finally, let us briefly mention two measurements that are presumably experimentally most feasible to implement in common quantum optics laboratories: *heterodyne* and *homodyne* measurements [**?**]. Heterodyne measurement is a POVM whose elements are given by all the coherent states, suitably normalised. Specifically, these POVM elements are $\{\frac{1}{(2\pi)^n} \mathrm{d}^{2n}\mathbf{r}\, |\mathbf{r}\rangle\!\langle\mathbf{r}|\}_{r \in \mathbb{R}^{2n}}$, which sum up to the identity thanks to the over-complete relation of coherent states [**?**] according to

$$\frac{1}{(2\pi)^n} \int_{\mathbb{R}^{2n}} \mathrm{d}^{2n}\mathbf{r}\, |\mathbf{r}\rangle\!\langle\mathbf{r}| = \mathbb{1} . \tag{S72}$$

Moreover, a homodyne measurement entails measuring a quadrature observable, which is a component of either the quadrature operator vector $\hat{\mathbf{R}}$ or, more generally, a rotated version $U_S^\dagger \hat{\mathbf{R}} U_S = S\hat{\mathbf{R}}$, where U_S represents the symplectic unitary associated with the symplectic matrix S.

D. Preliminaries on quantum learning theory

In this section, we introduce the concept of *quantum state tomography*, which forms the basis of our investigation. We start by formulating the problem of quantum state tomography.

Problem S11 (Quantum state tomography)**.** *Let $n \in \mathbb{N}$ be the number of modes/qudits. Let $\mathcal{S} \subseteq \mathcal{D}(\mathcal{H}^{\otimes n})$ be a subset of the set of quantum states $\mathcal{D}(\mathcal{H}^{\otimes n})$. Consider $\varepsilon, \delta \in (0,1)$ and $N \in \mathbb{N}$. Let $\rho \in \mathcal{S}$ be an unknown quantum state. Given access to N copies of ρ, the goal is to provide a classical description of a quantum state $\tilde{\rho}$ such that*

$$\Pr\left(\frac{1}{2}\|\tilde{\rho} - \rho\|_1 \leq \varepsilon\right) \geq 1 - \delta . \tag{S73}$$

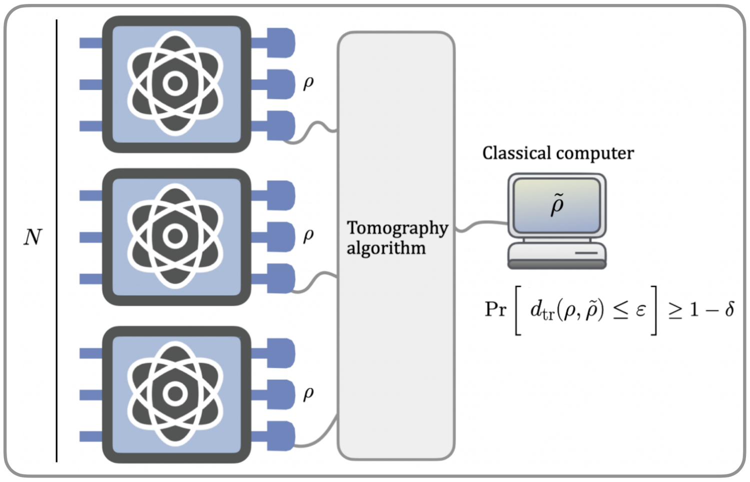

Figure S2. Pictorial representation of a quantum state tomography algorithm. Given access to N copies of an unknown state ρ, the goal of a tomography algorithm is to construct a classical description of a state $\tilde{\rho}$ that serves as a 'good approximation' of the true unknown state ρ. Mathematically, the error incurred in such an approximation is measured by the trace distance between ρ and $\tilde{\rho}$. This is the most meaningful way to measure the error incurred in a tomography algorithm, due to the operational meaning of the trace distance given by the Holevo-Helstrom theorem [**? ?**]. Additionally, since quantum measurements inherently yield probabilistic outcomes, the output $\tilde{\rho}$ is probabilistic rather than deterministic. We thus require that the probability that 'the trace distance is small' is high. Mathematically, this translates to $\Pr\left[\frac{1}{2}\|\tilde{\rho} - \rho\|_1 \le \varepsilon\right] \ge 1 - \delta$, where ε represents the trace distance error and δ denotes the failure probability. Fixed ε and δ, the minimum number of copies N required to achieve quantum state tomography with trace distance error ε and failure probability δ is called the sample complexity.

That is, with a probability $\ge 1 - \delta$, the trace distance between $\tilde{\rho}$ and ρ is at most ε. Here, ε is called the accuracy, while δ is called the failure probability.

Definition S12 (Sample, time, and memory complexity of a tomography algorithm)**.** *A quantum state tomography algorithm is an algorithm that solves Problem S11. It is characterised by the sample complexity, i.e., the number of copies of the unknown state needed by the algorithm, represented by N in Problem S11. The time complexity of the tomography algorithm is the amount of classical and quantum computation time required to execute the algorithm. The memory complexity quantifies the amount of classical memory required by the algorithm.*

We say that a tomography algorithm is *efficient* if its sample, time, and memory complexity scale polynomially in the number of modes/qudits n. It is worth noting that the time complexity of an algorithm always upper bound its memory complexity, as well as its sample complexity. In Fig. S2 we provide a pictorial representation of a quantum state tomography algorithm.

In the literature, several tomography algorithms [**? ? ?**] are tailored to finite-dimensional rank-r quantum states $\rho \in \mathcal{D}(\mathbb{C}^D)$, where D is the dimension of the Hilbert space. In the case of n-qudits systems with local dimension d, then the dimension of the Hilbert space is $D = d^n$. If no assumptions on the states are known, then it is known that any tomography algorithm needs at least $\tilde{\Omega}(rD/\varepsilon^2)$ copies of the unknown state to solve the tomography problem [**?**], where ε is the accuracy parameter. In this case, the tilde above the Big-O asymptotic functions means that we neglect logarithmic factors. There are algorithms that match these lower bounds, i.e., they require $\tilde{O}(rD/\varepsilon^2)$ copies of the unknown state to solve the tomography problem [**? ?**]. Such algorithms that achieve optimal performances use entangled measurements between multiple copies of the unknown state. However, there are also algorithms, which might be more experimentally feasible, that use *unentangled* or *single-copies* measurements between the queried copies of the unknown state; in this case, a lower bound $\tilde{\Omega}(r^2D/\varepsilon^2)$ is known to hold [**?**]. Also in this case, there are known algorithms which achieve optimal performances [**?**]. In the case of an n-qudits system, all these algorithms necessarily scale exponentially with the number of qudits n. However, if further restrictions on the class of states are provided, then one might achieve a sample and time complexity scaling which is polynomial in n (or, equivalently, logarithmic in D). In the finite-dimensional case, only a few classes of states are currently known to be efficiently learnable, such as matrix product states [**?**], finitely-correlated states [**?**], states prepared by shallow quantum circuits [**?**], quantum phase states [**?**], stabiliser states [**?**], states prepared by Clifford circuits 'doped' with at most $O(\log(n))$ T-gates [**? ? ?**], fermionic Gaussian states [**?**], and states prepared by fermionic Gaussian circuits 'doped' with at most $O(\log(n))$ non-Gaussian gates [**?**]. For a detailed literature review of quantum learning theory, see Ref. [**?**].

Given a tomography algorithm, we define a shorthand for denoting its sample and time complexity that will be useful later.

Definition S13 (Sample and time complexity)**.** *Let $\rho \in \mathcal{D}(\mathbb{C}^D)$ be a D-dimensional quantum state where $D \in \mathbb{N}$. Let $\varepsilon, \delta \in (0,1)$. Let $\mathcal{A}$ be a tomography algorithm. We denote as $N_{\mathrm{tom}}(\mathcal{A}, D, \varepsilon, \delta)$ and $T_{\mathrm{tom}}(\mathcal{A}, D, \varepsilon, \delta)$, respectively, the sample and time*

complexity of the algorithm $\mathcal{A}$ to solve the tomography problem of the state ρ with accuracy at most ε and a failure probability at most δ. That is, given access to $N_{\mathrm{tom}}(\mathcal{A}, D, \varepsilon, \delta)$ copies of ρ and using at most $T_{\mathrm{tom}}(\mathcal{A}, D, \varepsilon, \delta)$ computational time, the algorithm $\mathcal{A}$ outputs, with probability $\geq 1-\delta$, a classical description of a state $\tilde{\rho}$ such that

$$\frac{1}{2}\|\tilde{\rho}-\rho\|_1 \leq \varepsilon. \tag{S74}$$

We now present a standard proof technique to provide lower bound on the sample complexity of a tomography algorithm (see, e.g., Ref. [**?**]), which relies on the use of Fano's inequality [**?**] and Holevo's bound [**?**].

Lemma S14 (Lower bound on the sample complexity)**.** *Let $M \in \mathbb{N}$ and $\varepsilon > 0$. Consider a distance metric $d(\cdot,\cdot)$ between quantum states (e.g., trace distance). Define the discrete set $\mathcal{M}_\varepsilon := \{\rho_1, \dots, \rho_M\}$ as a set of quantum states such that for every $i \neq j \in [M]$, $d(\rho_i, \rho_j) > 2\varepsilon$. Let $\mathcal{S}$ be a subset of quantum states such that $\mathcal{M}_\varepsilon \subseteq \mathcal{S}$. Any tomography algorithm that learns states within the metric $d(\cdot,\cdot)$ from the set $\mathcal{S}$ with accuracy $\leq \varepsilon$ and failure probability $\leq \delta$ must use a number of state copies N satisfying:*

$$\chi \geq (1-\delta)\log_2(M) - H_2(\delta), \tag{S75}$$

where $\chi := S\left(\frac{1}{M}\sum_{j=1}^M \rho_j^{\otimes N}\right) - \frac{1}{M}\sum_{j=1}^M S(\rho_j^{\otimes N})$ is the Holevo information, $S(\cdot)$ denotes the von Neumann entropy, and $H_2(x) := -x\log_2 x - (1-x)\log_2(1-x)$ is the binary entropy. For instance, if the states in $\mathcal{M}_\varepsilon$ live in a 2^n dimensional Hilbert space, then this implies that the number of copies N satisfies

$$N \geq \Omega\left(\frac{\log_2(M)}{n}\right). \tag{S76}$$

Proof. Consider a communication protocol between Alice and Bob. First, define a codebook that Alice will use to encode classical information in quantum states: each number $i \in [M]$ is associated with the quantum state $\rho_i \in \mathcal{M}_\varepsilon$. Alice samples uniformly a number $x \in [M]$. Let us denote the random variable associated to x with X. Then Alice prepares the state $\rho_x^{\otimes N}$ and sends it to Bob. Now Bob runs the tomography algorithms and learns the classical description of a matrix $\hat{\rho}$ such that $d(\rho_x, \hat{\rho}) \leq \varepsilon$ with failure probability $\leq \delta$. In case the tomography protocol succeeds, this would imply that for all $j \in [M]$ such that $j \neq x$, we have $d(\rho_j, \hat{\rho}) > \varepsilon$, because

$$d(\rho_j, \hat{\rho}) \geq d(\rho_j, \rho_x) - d(\rho_x, \hat{\rho}) > 2\varepsilon - \varepsilon = \varepsilon\,. \tag{S77}$$

Thus, in such a case, there exists only one element in the set $\mathcal{M}_\varepsilon$ that is ε-close to $\hat{\rho}$ and this has to be ρ_x. Hence, by going through all the M states in $\mathcal{M}_\varepsilon$ and for each of them computing the trace distance with $\hat{\rho}$, Bob can find the number $x \in [M]$ corresponding to ρ_x (with probability $\geq 1-\delta$). Thus, at the ends of this procedure, Bob selects a number $y \in [M]$ and with probability $\geq 1-\delta$, it will coincide with x, i.e., the message sent by Alice. Therefore, the probability that Bob decodes the wrong message is $\leq \delta$. Call Y the random variable associated with y. By Fano's inequality (see Ref. [**?**]), we have

$$I(X:Y) \geq (1-\delta)\log_2(M) - H_2(\delta), \tag{S78}$$

where $I(X:Y)$ is the mutual information (see, e.g. Ref. [**?**] for the standard definition) and $H_2(\cdot)$ is the binary entropy. By the data processing inequality [**?**], we have

$$I(X:Y) \leq I(X:Z), \tag{S79}$$

where Z is the random variable associated with the POVM outcome associated with the tomography algorithm. By Holevo's theorem [**? ?**], we have

$$I(X:Z) \leq S\left(\frac{1}{M}\sum_{j=1}^M \rho_j^{\otimes N}\right) - \frac{1}{M}\sum_{j=1}^M S(\rho_j^{\otimes N}) = \chi. \tag{S80}$$

Hence, we get

$$\chi \geq (1-\delta)\log_2(M) - H_2(\delta). \tag{S81}$$

If the states in $\mathcal{M}_\varepsilon$ live in a 2^n dimensional Hilbert space, then we have

$$\chi \leq S\left(\frac{1}{M}\sum_{j=1}^M \rho_j^{\otimes N}\right) \leq N\log(2^n). \tag{S82}$$

This implies that

$$N \geq \frac{1}{\log_2(2^n)}\left((1-\delta)\log_2(M) - H_2(\delta)\right) = \Omega\left(\frac{\log_2(M)}{n}\right). \tag{S83}$$

□

E. Preliminaries on ε-nets

In this section, we introduce key concepts related to ε-nets [?] before delving into our novel results on the topic. Let us begin by giving definitions relative to the notion of ε-covering net [?].

Definition 1 (ε-covering net/number)**.** Let $(T, \|\cdot\|)$ be a normed space with distance induced by the norm $\|\cdot\|$, $K \subseteq T$ be a subset, and let $\varepsilon > 0$. We define

- **ε-covering net:** A subset $C \subseteq K$ is an ε-covering net of K if, for every $x \in K$, there exists $x_0 \in C$ such that $\|x - x_0\| \leq \varepsilon$.
- **ε-covering number:** The covering number of K, denoted as $\mathcal{C}(K, \|\cdot\|, \varepsilon)$, is the smallest cardinality of the ε-covering nets of K.
- **Optimal ε-covering:** $C \subseteq K$ is an optimal ε-covering of K if and only if $|C| = \mathcal{C}(K, \|\cdot\|, \varepsilon)$, where $|C|$ denotes the cardinality of C.

We now move to define definitions related to the notion of ε-packing net [?].

Definition 2 (ε-packing net/number)**.** Let $(T, \|\cdot\|)$ be a normed space with distance induced by the norm $\|\cdot\|$, $K \subseteq T$ be a subset, and let $\varepsilon > 0$. We define:

- **ε-packing net:** A subset $P \subseteq K$ is an *ε-packing net* of K if $\|x - y\| > \varepsilon$ for every $x, y \in P$.
- **ε-packing number:** The packing number of K, denoted as $\mathcal{P}(K, \|\cdot\|, \varepsilon)$, is the largest cardinality of the ε-packing nets of K.
- **Optimal ε-packing:** $P \subseteq K$ is is an optimal ε-covering of K if and only if $|P| = \mathcal{P}(K, \|\cdot\|, \varepsilon)$.

A result in the theory of ε-net [?] connects the ε-covering number $\mathcal{C}(K, \|\cdot\|, \varepsilon)$ to the ε-packing number $\mathcal{P}(K, \|\cdot\|, \varepsilon)$.

Lemma S15 (Ref. [?], Lemma 4.2.8) **.** *Let $(T, \|\cdot\|)$ be a normed space, $K \subseteq T$ be a subset, and let $\varepsilon > 0$. Then, it holds that*

$$\mathcal{P}(K, \|\cdot\|, \varepsilon) \geq \mathcal{C}(K, \|\cdot\|, \varepsilon) \geq \mathcal{P}(K, \|\cdot\|, 2\varepsilon)\,. \tag{S84}$$

F. Our results on ε-nets

In this section we will present our results concerning ε-nets. We will begin by proving a lemma that relates the covering number of a $(d-1)$-dimensional sphere with unit radius to the covering number of the surface of a d-dimensional sphere with unit radius, where $d \in \mathbb{N}_+$.

Lemma S16 (Covering numbers)**.** *Consider the normed vector space $(\mathbb{R}^d, \|\cdot\|_2)$, where $d \in \mathbb{N}_+$. Let $B_1(d)$ be the d-dimensional unit ball and $\partial B_1(d)$ its boundary, defined as*

$$B_1(d) := \{x \in \mathbb{R}^d \,:\, \|x\|_2 \leq 1\}, \tag{S85}$$

$$\partial B_1(d) := \{x \in \mathbb{R}^d \,:\, \|x\|_2 = 1\}. \tag{S86}$$

For all $d \in \mathbb{N}_+$ and $\varepsilon > 0$, the covering numbers of $B_1(d-1)$ and $\partial B_1(d)$ are related as

$$\mathcal{C}(\partial B_1(d), \|\cdot\|_2, \varepsilon) \geq \mathcal{C}(B_1(d-1), \|\cdot\|_2, \varepsilon). \tag{S87}$$

Proof. Consider an *optimal* ε-covering net of $\partial B_1(d)$, denote with $C^{\varepsilon,\text{opt}}_{\partial B_1(d)}$. Note that for any $y \in B_1(d-1)$, the d-dimensional vector $v(y) := \left(y, \sqrt{1 - \|y\|_2^2}\right)$ satisfies $v(y) \in \partial B_1(d)$. Therefore, for any $y \in B_1(d-1)$ there exists $x \in C^{\varepsilon,\text{opt}}_{\partial B_1(d)}$ such that

$$\|v(y) - (x_1, \ldots, x_d)\|_2 \leq \varepsilon. \tag{S88}$$

Thus, we also have that

$$\|y-(x_1,\dots,x_{d-1})\|_2 \le \sqrt{\|y-(x_1,\dots,x_{d-1})\|_2^2+(\sqrt{1-\|y\|_2^2}-x_d)^2} = \|v(y)-(x_1,\dots,x_d)\|_2 \le \varepsilon\,. \tag{S89}$$

This implies that the set

$$C^{\varepsilon}_{B_1(d-1)} := \{(x_1,\dots,x_{d-1})\in\mathbb{R}^{d-1} \,|\, x\in C^{\varepsilon,\mathrm{opt}}_{\partial B_1(d)}\} \tag{S90}$$

is an ε-covering net for $B_1(d-1)$. Thus, we have

$$\mathcal{C}(\partial B_1(d),\|\cdot\|_2,\varepsilon) = |C^{\varepsilon,\mathrm{opt}}_{\partial B_1(d)}| \ge |C^{\varepsilon}_{B_1(d-1)}| \ge \mathcal{C}(B_1(d-1),\|\cdot\|_2,\varepsilon) \tag{S91}$$

where the first step follows from the definition of covering number, the second step follows from the fact that $C^{\varepsilon}_{B_1(d-1)}$ contains at most the same number of elements as $C^{\varepsilon,\mathrm{opt}}_{\partial B_1(d)}$, and the last inequality follows from the fact that the ε-covering number is a lower bound on the cardinality of every possible ε-covering net. This concludes the proof. □

We now present a lemma which relates the covering number of the set of pure states density matrices with respect to the (matrix) 1-norm and the covering number of the set of pure states vectors with respect to the (vector) 2-norm.

Lemma S17 (Covering numbers revisited)**.** *Let $d\in\mathbb{N}$ and $\varepsilon\in[0,1)$. We define the sets $\mathcal{S}_{|*\rangle\langle*|}$ and $\mathcal{S}_{|*\rangle}$, as*

$$\mathcal{S}_{|*\rangle\langle*|} := \{|v\rangle\langle v| \,:\, |v\rangle\in\mathbb{C}^d \text{ such that } |\langle v|v\rangle|=1\}, \tag{S92}$$

$$\mathcal{S}_{|*\rangle} := \{|v\rangle \,:\, |v\rangle\in\mathbb{C}^d \text{ such that } |\langle v|v\rangle|=1\}. \tag{S93}$$

It follows the relation

$$\mathcal{C}(\mathcal{S}_{|*\rangle\langle*|},\|\cdot\|_1,\varepsilon) \ge \frac{\varepsilon}{8\pi}\mathcal{C}(\mathcal{S}_{|*\rangle},\|\cdot\|_2,\varepsilon)\,. \tag{S94}$$

between their covering numbers.

Proof. Let $C^{\varepsilon,\mathrm{opt}}_{\mathcal{S}_{|*\rangle\langle*|}} = \{|\psi_1\rangle\langle\psi_1|,\dots,|\psi_M\rangle\langle\psi_M|\}$ be an optimal ε-covering net of the set $\mathcal{S}_{|*\rangle\langle*|}$ with respect to the trace norm $\|\cdot\|_1$, where we defined $M:=\mathcal{C}(\mathcal{S}_{|*\rangle\langle*|},\|\cdot\|_1,\varepsilon)$. For each $j\in[M]$, we take $|\phi_j\rangle$ to be a state such that $|\phi_j\rangle\langle\phi_j| = |\psi_j\rangle\langle\psi_j|$. Let $\delta>0$ be a number that we will fix later in terms of ε. We define the set

$$C_\delta := \left\{ e^{i\delta m}|\phi_j\rangle \,:\, j\in[M],\, m\in\left\{0,1,\dots,\left\lfloor\frac{2\pi}{\delta}\right\rfloor\right\}\right\}\,. \tag{S95}$$

Let us show that C_δ is an $(\frac{\varepsilon}{\sqrt{2}}+\delta)$-covering net of $\mathcal{S}_{|*\rangle}$ with respect to the 2-norm $\|\cdot\|_2$. To this end, fixed a state vector $|\psi\rangle\in\mathbb{C}^d$, it suffices to construct a state vector $|\tilde{\psi}\rangle\in C_\delta$ such that

$$\|\,|\psi\rangle-|\tilde{\psi}\rangle\,\|_2 \le \frac{\varepsilon}{\sqrt{2}}+\delta\,. \tag{S96}$$

By definition of $C^{\varepsilon,\mathrm{opt}}_{\mathcal{S}_{|*\rangle\langle*|}}$, there exists $j\in[M]$ such that $\|\,|\phi_j\rangle\langle\phi_j|-|\psi\rangle\langle\psi|\,\|_1\le\varepsilon$. Let $\bar{m}:=\lfloor\frac{2\pi-\arg(\langle\psi|\phi_j\rangle)}{\delta}\rfloor$, where $\arg(\langle\psi|\phi_j\rangle)$ is such that $\langle\psi|\phi_j\rangle = |\langle\psi|\phi_j\rangle|e^{i\arg(\langle\psi|\phi_j\rangle)}$, and let us define the state vector $|\tilde{\psi}\rangle := e^{i\delta\bar{m}}|\phi_j\rangle\in C_\delta$. Let us now show that the so-defined $|\tilde{\psi}\rangle$ satisfies Eq. (S96) according to

$$\begin{aligned}
\|\,|\psi\rangle-|\tilde{\psi}\rangle\,\|_2 &= \|\,|\psi\rangle - e^{i\delta\bar{m}}|\phi_j\rangle\,\|_2\\
&\le \|\,|\psi\rangle - e^{-i\arg(\langle\psi|\phi_j\rangle)}|\phi_j\rangle\,\|_2 + \|e^{-i\arg(\langle\psi|\phi_j\rangle)}|\phi_j\rangle - e^{i\delta\bar{m}}|\phi_j\rangle\,\|_2\\
&\overset{\text{(i)}}{\le} \frac{1}{\sqrt{2}}\|\,|\psi\rangle\langle\psi|-|\phi_j\rangle\langle\phi_j|\,\|_1 + \|e^{-i\arg(\langle\psi|\phi_j\rangle)}|\phi_j\rangle - e^{i\delta\bar{m}}|\phi_j\rangle\,\|_2\\
&\le \frac{\varepsilon}{\sqrt{2}} + |e^{i(2\pi-\arg(\langle\psi|\phi_j\rangle))} - e^{i\delta\bar{m}}|\\
&\overset{\text{(ii)}}{\le} \frac{\varepsilon}{\sqrt{2}} + |2\pi-\arg(\langle\psi|\phi_j\rangle)-\delta\bar{m}|\\
&\le \frac{\varepsilon}{\sqrt{2}}+\delta\,.
\end{aligned} \tag{S97}$$

Here, (i) follows from the fact that

$$\left\| |\psi\rangle - e^{-i\arg(\langle\psi|\phi_j\rangle)}\,|\phi_j\rangle \right\|_2 = \sqrt{2\left(1-\mathrm{Re}\left(e^{-i\arg(\langle\psi|\phi_j\rangle)}\,\langle\psi|\phi_j\rangle\right)\right)} \\ = \sqrt{2\left(1-|\langle\psi|\phi_j\rangle|\right)} \\ \leq \sqrt{2\left(1-|\langle\psi|\phi_j\rangle|^2\right)} \\ = \frac{1}{\sqrt{2}}\left\| |\psi\rangle\!\langle\psi| - |\phi_j\rangle\!\langle\phi_j| \right\|_1 , \tag{S98}$$

where in the last step we have used the well-known formula for the trace distance between two pure states (see, e.g. Ref. [**?**]). Moreover, (ii) is a consequence of the fact that for any $x, y \in \mathbb{R}$ it holds that

$$|e^{ix} - e^{iy}| = \sqrt{2}\sqrt{1-\cos(x-y)} \leq |x-y|\,. \tag{S99}$$

Hence, we have proved that C_δ is a $(\frac{\varepsilon}{\sqrt{2}}+\delta)$-covering net of $\mathcal{S}_{|*\rangle}$ with respect to the 2-norm $\|\cdot\|_2$. Consequently, by setting $\delta := \varepsilon - \frac{\varepsilon}{\sqrt{2}}$, we have that $C_{\varepsilon-\frac{\varepsilon}{\sqrt{2}}}$ is an ε-covering net of $\mathcal{S}_{|*\rangle}$ with respect to the 2-norm $\|\cdot\|_2$. In particular, its cardinality has to satisfy

$$|C_{\varepsilon-\frac{\varepsilon}{\sqrt{2}}}| \geq \mathcal{C}(\mathcal{S}_{|*\rangle}, \|\cdot\|_2, \varepsilon)\,. \tag{S100}$$

Consequently, we can conclude that

$$\mathcal{C}(\mathcal{S}_{|*\rangle\langle*|}, \|\cdot\|_1, \varepsilon) = |C^{\varepsilon,\mathrm{opt}}_{\mathcal{S}_{|*\rangle\langle*|}}| \\ \geq \left(\left\lfloor \frac{2\pi}{\varepsilon-\frac{\varepsilon}{\sqrt{2}}}\right\rfloor + 1\right)^{-1} |C_{\varepsilon-\frac{\varepsilon}{\sqrt{2}}}| \\ \geq \left(\left\lfloor \frac{2\pi}{\varepsilon-\frac{\varepsilon}{\sqrt{2}}}\right\rfloor + 1\right)^{-1} \mathcal{C}(\mathcal{S}_{|*\rangle}, \|\cdot\|_2, \varepsilon) \\ \geq \frac{\varepsilon}{8\pi}\mathcal{C}(\mathcal{S}_{|*\rangle}, \|\cdot\|_2, \varepsilon), \tag{S101}$$

where the first inequality follows by a simple counting argument, based on Eq. (S95), which shows that $|C_\delta| \leq |C^{\varepsilon,\mathrm{opt}}_{\mathcal{S}_{|*\rangle\langle*|}}|(\lfloor\frac{2\pi}{\delta}\rfloor+1)$, and the last inequality follows by inspection. □

The following lemma establishes a lower bound on the ε-packing number of the set of pure state density matrices with respect to the trace norm.

Lemma S18 (ε-packings)**.** *Let $d \in \mathbb{N}$ and $\varepsilon \in [0,1)$. Define the set of pure state density matrices $\mathcal{S}_{|*\rangle\langle*|}$ as*

$$\mathcal{S}_{|*\rangle\langle*|} := \{|v\rangle\!\langle v| \,:\, |v\rangle \in \mathbb{C}^d \text{ such that } |\langle v|v\rangle| = 1\}. \tag{S102}$$

Then, we have

$$\mathcal{P}(\mathcal{S}_{|*\rangle\langle*|}, \|\cdot\|_1, \varepsilon) \geq \frac{1}{8\pi}\varepsilon^{-2(d-1)}\,. \tag{S103}$$

Proof. Consider the set of pure state vectors $\mathcal{S}_{|*\rangle\langle*|}$ defined as

$$\mathcal{S}_{|*\rangle} := \{|v\rangle \,:\, |v\rangle \in \mathbb{C}^d \text{ such that } |\langle v|v\rangle| = 1\}. \tag{S104}$$

Let $B_1(k)$ be the k-dimensional unit ball and $\partial B_1(k)$ its boundary, with $k \in \mathbb{N}$ and $r \in \mathbb{R}_+$, defined as

$$B_r(k) := \{x \in \mathbb{R}^k \,:\, \|x\|_2 \leq r\}, \tag{S105}$$
$$\partial B_r(k) := \{x \in \mathbb{R}^k \,:\, \|x\|_2 = r\}. \tag{S106}$$

We can establish the chain of inequalities

$$
\begin{aligned}
\mathcal{P}(\mathcal{S}_{|*\rangle\langle*|}, \|\cdot\|_1, \varepsilon) &\overset{\text{(i)}}{\geq} \mathcal{C}(\mathcal{S}_{|*\rangle\langle*|}, \|\cdot\|_1, \varepsilon) \\
&\overset{\text{(ii)}}{\geq} \frac{\varepsilon}{8\pi}\mathcal{C}(\mathcal{S}_{|*\rangle}, \|\cdot\|_2, \varepsilon) \\
&\overset{\text{(iii)}}{=} \frac{\varepsilon}{8\pi}\mathcal{C}(\partial B_1(2d), \|\cdot\|_2, \varepsilon) \\
&\overset{\text{(iv)}}{\geq} \frac{\varepsilon}{8\pi}\mathcal{C}(B_1(2d-1), \|\cdot\|_2, \varepsilon) \\
&\overset{\text{(v)}}{\geq} \frac{\varepsilon}{8\pi}\frac{\mathrm{Vol}(B_1(2d-1))}{\mathrm{Vol}(B_\varepsilon(2d-1))} \\
&\overset{\text{(vi)}}{=} \frac{\varepsilon^{-2(d-1)}}{8\pi}\,.
\end{aligned}
\tag{S107}
$$

Here, (i) and (ii) follow from Lemma S15 and Lemma S17, respectively. Moreover, (iii) stems from viewing the set of pure states $\mathcal{S}_{|*\rangle}$ as the boundary of the $2d$-dimensional real ball $B_1(2d)$. In (iv), we apply Lemma S16. In (v), we denote $\mathrm{Vol}(B_1(2d-1))$ as the volume of the $(2d-1)$-dimensional ball with unit radius and $\mathrm{Vol}(B_\varepsilon(2d-1))$ as the volume of the $(2d-1)$-dimensional ball with radius ε. We then exploit a simple counting argument to get $\mathrm{Vol}(B_\varepsilon(2d-1))\mathcal{C}(B_1(2d-1), \|\cdot\|_2, \varepsilon) \geq \mathrm{Vol}(B_1(2d-1))$. Finally, in (vi), we use the known fact that the volume of an n-dimensional ball with radius r is $r^n\mathrm{Vol}(B_1(2d-1))$. ☐

We now present a lemma which relates the covering number of the set of quantum states on $\mathbb{C}^d$ with the covering number of the set of Hermitian matrices with trace norm smaller than one.

Lemma S19 (Covering number of the set of Hermitian matrices)**.** *Let $\varepsilon > 0$ and $d \in \mathbb{N}_+$. Let $\mathcal{D}(\mathbb{C}^d)$ and $\mathrm{Herm}_{\|*\|_1\leq 1}(d)$ be respectively the set of quantum states on $\mathbb{C}^d$ and the set of Hermitian matrices with trace norm smaller than one, i.e.,*

$$
\begin{aligned}
\mathcal{D}(\mathbb{C}^d) &:= \left\{\rho \in \mathbb{C}^{d,d} \,:\, \rho \geq 0,\ \mathrm{Tr}\,\rho = 1\right\}, \\
\mathrm{Herm}_{\|*\|_1\leq 1}(d) &:= \left\{X \in \mathbb{C}^{d,d} \,:\, X^\dagger = X,\ \|X\|_1 \leq 1\right\}.
\end{aligned}
\tag{S108}
$$

Then, the relation between their covering numbers

$$
\mathcal{C}(\mathcal{D}(\mathbb{C}^d), \|\cdot\|_1, \varepsilon) \geq \sqrt{\mathcal{C}(\mathrm{Herm}_{\|*\|_1\leq 1}(d-1), \|\cdot\|_1, 2\varepsilon)}\,. \tag{S109}
$$

follows.

Proof. The strategy of the proof is to construct a covering net for $\mathrm{Herm}_{\|*\|_1\leq 1}(d-1)$ from an optimal ε-covering net of set of quantum states $\mathcal{D}(\mathbb{C}^d)$. Let $C^{\varepsilon,\mathrm{opt}}_{\mathcal{D}(\mathbb{C}^d)}$ be an optimal ε-covering net for $\mathcal{D}(\mathbb{C}^d)$. Let us fix $X \in \mathrm{Herm}_{\|*\|_1\leq 1}(d-1)$, and let us write it in its Jordan–Hahn decomposition [?] as follows, $X = X_1 - X_2$, where $X_1, X_2 \geq 0$ and $X_1X_2 = X_2X_1 = 0$. Since

$$
1 \geq \|X\|_1 = \mathrm{Tr}\,X_1 + \mathrm{Tr}\,X_2\,, \tag{S110}
$$

it follows that

$$
\begin{aligned}
1 - \mathrm{Tr}\,X_1 \geq 0\,, \\
1 - \mathrm{Tr}\,X_2 \geq 0\,.
\end{aligned}
\tag{S111}
$$

Let us consider the $d \times d$ block diagonal matrices

$$
\begin{aligned}
\bar{X}_1 &:= \begin{pmatrix} X_1 & \mathbf{0} \\ \mathbf{0}^\intercal & 1 - \mathrm{Tr}\,X_1 \end{pmatrix}, \\
\bar{X}_2 &:= \begin{pmatrix} X_2 & \mathbf{0} \\ \mathbf{0}^\intercal & 1 - \mathrm{Tr}\,X_2 \end{pmatrix},
\end{aligned}
\tag{S112}
$$

where $\mathbf{0}$ is the $(d-1)$-dimensional zero vector. Hence, both $\bar{X}_1$ and $\bar{X}_2$ are density operators, i.e., $\bar{X}_1, \bar{X}_2 \in \mathcal{D}(\mathbb{C}^d)$. By definition of $C^{\varepsilon,\mathrm{opt}}_{\mathcal{D}(\mathbb{C}^d)}$, there exist $\rho_1, \rho_2 \in C^{\varepsilon,\mathrm{opt}}_{\mathcal{D}(\mathbb{C}^d)}$ such that $\|\bar{X}_1 - \rho_1\|_1 \leq \varepsilon$ and $\|\bar{X}_2 - \rho_2\|_1 \leq \varepsilon$. Given a matrix A, let us denote

as $A|_n$ its $n \times n$ top-left block. Specifically, we denote as $\rho_1|_{d-1}$ and as $\rho_2|_{d-1}$ the $(d-1) \times (d-1)$ top-left blocks of ρ_1 and ρ_2, respectively. We have that

$$\begin{aligned}\left\|X - (\rho_1|_{d-1} - \rho_2|_{d-1})\right\|_1 &= \|X_1 - X_2 - (\rho_1|_{d-1} - \rho_2|_{d-1})\|_1 \\ &\leq \|X_1 - \rho_1|_{d-1}\|_1 + \|X_2 - \rho_2|_{d-1}\|_1 \\ &\overset{\text{(i)}}{\leq} \|\bar{X}_1 - \rho_1\|_1 + \|\bar{X}_2 - \rho_2\|_1 \\ &\leq 2\varepsilon \,.\end{aligned} \tag{S113}$$

Here, in (i), we have exploited the general fact that the trace norm of any matrix A is an upper bound on the trace norm of its $n \times n$ top-left block $A|_n$, i.e., $\|A|_n\|_1 \leq \|A\|_1$. This latter fact can be easily proved by exploiting the *minimax principle for singular values* [**?** , Problem III.6.1]. Therefore, we have proved that the set

$$S := \{\rho_1|_{d-1} - \rho_2|_{d-1} \;:\; \rho_1, \rho_2 \in C^{\varepsilon,\mathrm{opt}}_{\mathcal{D}(\mathbb{C}^d)}\} \tag{S114}$$

constitutes a 2ε-covering net of $\mathrm{Herm}_{\|*\|_1 \leq 1}(d-1)$, and thus $|S| \geq \mathcal{C}(\mathrm{Herm}_{\|*\|_1 \leq 1}(d-1), \|\cdot\|_1, 2\varepsilon)$. Therefore, we conclude that

$$\mathcal{C}(\mathrm{Herm}_{\|*\|_1 \leq 1}(d-1), \|\cdot\|_1, 2\varepsilon) \leq |S| \leq |C^{\varepsilon,\mathrm{opt}}_{\mathcal{D}(\mathbb{C}^d)}|^2 = \mathcal{C}(\mathcal{D}(\mathbb{C}^d), \|\cdot\|_1, \varepsilon)^2 \,. \tag{S115}$$

□

We now present a lemma which gives a lower bound on the covering number of the set of Hermitian matrices with trace norm smaller than one.

Lemma S20 (Lower bound on the covering number)**.** *Let* $\varepsilon > 0$ *and* $d \in \mathbb{N}_+$*. Let* $\mathrm{Herm}_{\|*\|_1 \leq 1}(d)$ *be the set of Hermitian matrices with trace norm smaller than one, i.e.,*

$$\mathrm{Herm}_{\|*\|_1 \leq 1}(d) := \left\{X \in \mathbb{C}^{d,d} \;:\; X^\dagger = X,\; \|X\|_1 \leq 1\right\} \,. \tag{S116}$$

Then, its covering number can be lower bounded by

$$\mathcal{C}(\mathrm{Herm}_{\|*\|_1 \leq 1}(d), \|\cdot\|_1, \varepsilon) \geq \varepsilon^{-d^2} \,. \tag{S117}$$

Proof. Let us define the ball of $d \times d$ Hermitian matrices with radius r with respect the trace norm as

$$B_r(d) := \left\{X \in \mathbb{C}^{d,d} \;:\; X^\dagger = X,\; \|X\|_1 \leq r\right\} , \tag{S118}$$

Note that the ball of radius one $B_1(d)$ is exactly equal to the set $\mathrm{Herm}_{\|*\|_1 \leq 1}(d)$. The volume of the ball with radius r can be expressed in terms of the volume of the ball with unit radius as

$$\mathrm{Vol}(B_r(d)) = r^{d^2} \mathrm{Vol}(B_1(d)) \,. \tag{S119}$$

Let us prove this latter fact. First, note that there are d^2 independent real parameters $y_1, y_2, \ldots, y_{d^2}$ that define any $d \times d$ Hermitian matrix. Specifically, there are $d^2 - d$ independent real parameters for the off diagonal elements, and d independent real parameters for the diagonal ones. Given the d^2-dimensional vector $y = (y_1, y_2, \ldots, y_{d^2}) \in \mathbb{R}^{d^2}$ of such parameters, let $X(y)$ be the associated Hermitian matrix. Consequently, we have that

$$\mathrm{Vol}(B_r(d)) = \int\limits_{\substack{y \in \mathbb{R}^{d^2} \\ \|X(y)\|_1 \leq r}} 1 \, \mathrm{d}^{d^2} y \overset{\text{(i)}}{=} r^{d^2} \int\limits_{\substack{y \in \mathbb{R}^{d^2} \\ \|X(y)\|_1 \leq 1}} 1 \, \mathrm{d}^{d^2} y = r^{d^2} \mathrm{Vol}(B_1(d)) \,, \tag{S120}$$

where in (i) we made the change of variable $y \mapsto ry$ and we have exploited that $X(ry) = rX(y)$. Hence, we have proved (S119). Finally, a simple counting argument shows that

$$\mathcal{C}(\mathrm{Herm}_{\|*\|_1 \leq 1}(d), \|\cdot\|_1, \varepsilon) \geq \frac{\mathrm{Vol}(B_1(d))}{\mathrm{Vol}(B_\varepsilon(d))} \,, \tag{S121}$$

which, together with (S119), concludes the proof. □

The following corollary establishes a lower bound on the covering number of the set of (mixed) quantum states.

Corollary S21. *Let $\varepsilon > 0$ and $d \in \mathbb{N}_+$. Let $\mathcal{D}\big(\mathbb{C}^d\big)$ be the set of quantum states on $\mathbb{C}^d$, i.e.,*

$$\mathcal{D}\big(\mathbb{C}^d\big) := \left\{\rho \in \mathbb{C}^{d,d} \,:\, \rho \geq 0,\ \operatorname{Tr}\rho = 1\right\} . \tag{S122}$$

Then, its packing number can be lower bounded as follows

$$\mathcal{P}(\mathcal{D}\big(\mathbb{C}^d\big), \|\cdot\|_1, \varepsilon) \geq (2\varepsilon)^{-\frac{1}{2}(d-1)^2} \tag{S123}$$

Proof. The result follows from the following chain of inequalities:

$$\begin{aligned}\mathcal{P}(\mathcal{D}\big(\mathbb{C}^d\big), \|\cdot\|_1, \varepsilon) &\overset{\text{(i)}}{\geq} \mathcal{C}(\mathcal{D}\big(\mathbb{C}^d\big), \|\cdot\|_1, \varepsilon) \\ &\overset{\text{(ii)}}{\geq} \sqrt{\mathcal{C}(\mathrm{Herm}_{\|*\|_1\leq 1}(d-1), \|\cdot\|_1, 2\varepsilon)} \\ &\overset{\text{(iii)}}{\geq} (2\varepsilon)^{-\frac{1}{2}(d-1)^2} .\end{aligned} \tag{S124}$$

Here, (i), (ii), and (iii) follow from Lemma S15, Lemma S19, and Lemma S20, respectively. □

S2. TOMOGRAPHY OF MOMENT-CONSTRAINED STATES

In this section we consider the problem of quantum state tomography of continuous-variable systems. Since continuous-variable states are associated with an infinite dimensional Hilbert space, a first trivial observation is that quantum state tomography is impossible if one has no any extra prior information about the unknown state. However, in a practical scenario, experimentalists often possess knowledge about the energy budget available of their light sources — and hence about the mean energy of the unknown quantum state. Thus, it is crucial to analyse the problem of tomography of n-mode states subject to an energy constraint. Such energy constraint can be expressed as the following upper bound on the mean photon number of the unknown n-mode state ρ:

$$\mathrm{Tr}[\hat{N}_n\rho] \le nN_{\text{phot}}\,, \tag{S125}$$

where $\hat{N}_n$ is the n-mode photon number operator, and N_{phot} is a real number. In other words, one may have the extra prior information that the unknown quantum state is contained in the set

$$\left\{\rho \in \mathcal{D}\big(L^2(\mathbb{R}^n)\big) : \quad \mathrm{Tr}\left[\hat{N}_n\rho\right] \le nN_{\text{phot}}\right\} \tag{S126}$$

of energy constrained states. Here, the number of modes n and the energy constraint N_{phot} are *known* parameters, in the sense that the tomography algorithm may explicitly depend on their values. Note that we normalise the right-hand-side of (S125) with the factor n because the mean photon number $\mathrm{Tr}[\hat{N}_n\rho]$ is extensive, meaning that $\mathrm{Tr}[\hat{N}_n\sigma^{\otimes n}] = n\,\mathrm{Tr}[\hat{N}_1\sigma]$ for all single-mode states σ. In this section, we first consider the following question:

> Suppose there exists a tomography algorithm designed to learn, with ε-precision in trace distance and high probability, an unknown n-mode pure state ψ satisfying the energy constraint $\mathrm{Tr}[\hat{N}_n\psi] \le nN_{\text{phot}}$. What is the required sample complexity, represented by the number N of state copies?

In this section, we answer this question by showing that *any such tomography algorithm needs at least* $N = \Omega\left(\frac{N_{phot}}{\varepsilon^2}\right)^n$ *state copies*. This result imposes strong limitations on quantum state tomography of continuous-variable systems, due to the unfavourable scaling not only in the number of modes n but also in the trace-distance error ε. In contrast, in the finite-dimensional setting, quantum state tomography of an unknown n-qubit state needs at least $N = \tilde{\Theta}(2^n/\varepsilon^2)$ copies of the state (indicating a more favorable dependence on ε). Conversely, we also show that there exists a tomography algorithm that achieves the same sample complexity performances. Thus, we have the following main result:

Theorem S22 (Tomography of energy-constrained pure states - informal version)**.** *$N = \Theta\left(\frac{N_{phot}}{\varepsilon^2}\right)^n$ state copies are necessary and sufficient for quantum state tomography of energy-constrained n-mode pure states with trace-distance error ε.*

In the general case of n-mode energy-constained (possibly mixed) states we show the following:

Theorem S23 (Tomography of energy-constrained mixed states - informal version)**.** *$N = \Omega\left(\frac{N_{phot}}{\varepsilon}\right)^{2n}$ state copies are necessary for quantum state tomography of energy-constrained n-mode (possibly mixed) states with trace-distance error ε. Conversely, $N = O\left(\frac{N_{phot}}{\varepsilon^{3/2}}\right)^{2n}$ state copies are sufficient for this task.*

In practical scenarios, experimentalists often have knowledge not only about the mean energy but also about higher moments (e.g., energy variance) of their light sources. Therefore, it is meaningful to analyse tomography of states satisfying constraints on higher moments of energy. Mathematically, the k-th moment constraint can be expressed as

$$\left(\mathrm{Tr}[\hat{N}_n^k\rho]\right)^{1/k} \le nN_{\text{phot}}\,. \tag{S127}$$

Note that we normalise the right-hand-side of (S127) with the factor n because the k-th moment $\left(\mathrm{Tr}[\hat{N}_n^k\rho]\right)^{1/k}$ is extensive when evaluated on Fock states $\rho = |m\rangle\!\langle m|^{\otimes n}$, meaning that

$$\left(\mathrm{Tr}[\hat{N}_n^k\,|m\rangle\!\langle m|^{\otimes n}]\right)^{1/k} = n\left(\mathrm{Tr}[\hat{N}_1^k\,|m\rangle\!\langle m|]\right)^{1/k}. \tag{S128}$$

In summary, it is sometimes meaningful to possess the extra prior information that the unknown quantum state is contained in the following set of k-th moment-constrained states:

$$\mathcal{S}(n, N_{\text{phot}}, k) := \left\{\rho \in \mathcal{D}\big(L^2(\mathbb{R}^n)\big) : \quad \mathrm{Tr}\left(\left[\hat{N}_n^k\rho\right]\right)^{1/k} \le nN_{\text{phot}}\right\}\,, \tag{S129}$$

where we stress that n, N_{phot}, and k are *known* parameters. Note that the higher the value of k, the more prior extra information we have about the unknown quantum state. Indeed, this is established by the following simple result.

Lemma S24 (High moment-constraint implies low moment-constraint). *For any $k, q \in \mathbb{N}_+$ with $k \geq q$, it holds that*

$$\mathcal{S}(n, N_{phot}, k) \subseteq \mathcal{S}(n, N_{phot}, q). \tag{S130}$$

Proof. By Eq. S7 we have that, for any concave function f, any Hermitian matrix X, and any quantum state ρ, it holds that

$$\mathrm{Tr}(\rho f(X)) \leq f(\mathrm{Tr}(\rho X)). \tag{S131}$$

Now, the claim follows from the fact that

$$\left(\mathrm{Tr}\left[\rho\, \hat{N}_n^q\right]\right)^{1/q} = \left(\mathrm{Tr}\left[\rho\left(\hat{N}_n^k\right)^{q/k}\right]\right)^{1/q} \leq \left(\mathrm{Tr}\left[\rho\, \hat{N}_n^k\right]\right)^{1/k}, \tag{S132}$$

where in the first step we use the semi-definite positivity of $\hat{N}_n$, and the inequality follows from the concavity of the function $x \mapsto x^{q/k}$ for $x \geq 0$. □

In this section, we also analyse tomography of moment-constrained states and establish the following results.

Theorem S25 (Tomography of moment-constrained states - informal version). *Let ρ be an unknown n-mode state satisfying the k-th moment constraint $(\mathrm{Tr}[\hat{N}_n^k \rho])^{1/k} \leq nN_{phot}$. The following facts hold:*

a. *The number of copies of ρ required to achieve quantum state tomography with precision ε in trace distance scales at least as $\Omega\left(\frac{N_{phot}}{\varepsilon^{1/k}}\right)^{2n}$.*

b. *There exists a tomography algorithm with sample complexity scaling as $O\left(\frac{N_{phot}}{\varepsilon^{3/(2k)}}\right)^{2n}$.*

c. *If we assume the unknown state ρ to be pure, then $\Theta\left(\frac{N_{phot}}{\varepsilon^{2/k}}\right)^{n}$ copies of ρ are necessary and sufficient for tomography.*

We note that the upper bound $O\left(\frac{N_{\text{phot}}}{\varepsilon^{3/(2k)}}\right)^{2n}$ on the sample complexity of tomography of moment-constrained (mixed) states provided in Theorem S25 outperforms the one attainable through the CV classical shadow algorithm outlined in References [**?** **?**].

This section is organised as follows:

- In Subsection S2 A, we derive lower bounds on the sample complexity of tomography of moment-constrained states.
- In Subsection S2 B, we show upper bounds on the sample complexity by providing explicit tomography algorithms to learn moment-constrained states. Throughout this section, we also establish key properties of moment-constrained states, demonstrating that they can be effectively approximated by states with finite local dimension and rank.

Note: Throughout this section, we opted to simplify the notation in the use of parentheses in asymptotic expressions. For example, when we write $\Theta\left(\frac{N_{\text{phot}}}{\varepsilon^{2/k}}\right)^n$ we formally mean $\left[\Theta\left(\frac{N_{\text{phot}}}{\varepsilon^{2/k}}\right)\right]^n$. We stress that this notation omits only factors that are independent of the parameters of the problem, i.e., n, ε, and N_{phot}. For instance, with this notation, it is true that $\Theta\left(\frac{2N_{\text{phot}}}{\varepsilon^{2/k}}\right)^n = \Theta\left(\frac{N_{\text{phot}}}{\varepsilon^{2/k}}\right)^n$, but $\Theta\left(\frac{N_{\text{phot}}^2}{\varepsilon^{2/k}}\right)^n \neq \Theta\left(\frac{N_{\text{phot}}}{\varepsilon^{2/k}}\right)^n$. Importantly, to ensure clarity and avoid ambiguity, we express every sample complexity bound using both asymptotic notation and explicit exact expressions.

A. Sample complexity lower bounds

To establish lower bounds on the sample complexity of tomography in continuous variable systems, we rely on the concept of ε-nets (as discussed in Section S1 E). In the subsequent subsubsection S2 A 1, we delve into the analysis of sample complexity lower bounds for pure state tomography. Following that, we proceed to demonstrate lower bounds for mixed state tomography in subsubsection S2 A 2.

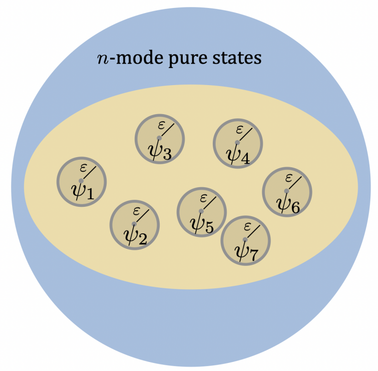

Figure S3. Pictorial representation of the set of all n-mode pure states (blue) and the set of of all n-mode pure states with bounded k-moment (yellow). We consider M moment-constrained pure states $\{\psi_1, \psi_2, \cdots, \psi_M\}$ such that they are at least 2ε-far from each other with respect to the trace distance.

1. *Lower bound for pure state tomography*

We now present and prove our main theorem concerning the number of copies necessary for learning moment-constrained pure states. The proof strategy hinges on the utilisation of Lemma S14, which relies on Fano's inequality and Holevo's bound, and on identifying a suitable 'packing' for the set of moment-constrained pure states.

Theorem S26 (Lower bound on sample complexity for moment-constrained pure states)**.** *Let us consider a tomography algorithm that learns, within a trace distance $\le \varepsilon$ and failure probability $\le \delta$, an arbitrary state belonging to the set*

$$\mathcal{S}_{pure}(n,k,N_{phot}) := \left\{ |\psi\rangle : n\text{-mode pure state}, \left(\mathrm{Tr}\left[\left(\hat{N}_n\right)^k |\psi\rangle\langle\psi|\right]\right)^{1/k} \le nN_{phot} \right\} \tag{S133}$$

of n-mode pure states with bounded k-moment. Then, such a tomography algorithm must use a number of state copies N satisfying

$$\begin{aligned} N &\ge \frac{1}{ng(N_{phot})}\left[2(1-\delta)\left(\frac{N_{phot}}{(12\varepsilon)^{2/k}} - \frac{1}{n}\right)^n - (1-\delta)\log_2(32\pi) - H_2(\delta)\right] \\ &= \Theta\left(\frac{N_{phot}}{\varepsilon^{2/k}}\right)^n . \end{aligned} \tag{S134}$$

Here, $g(x) := (x+1)\log_2(x+1) - x\log_2 x$ is the bosonic entropy, and $H_2(x) := -x\log_2 x - (1-x)\log_2(1-x)$ is the binary entropy.

Proof. Let $M \in \mathbb{N}$ be such that there exist M states

$$\{|\psi_1\rangle, |\psi_2\rangle, \ldots, |\psi_M\rangle\} \subseteq \mathcal{S}_{\text{pure}}(n,k,N_{\text{phot}}) \tag{S135}$$

such that for every $i \neq j \in [M]$ it holds that

$$\frac{1}{2}\|\psi_i - \psi_j\|_1 > 2\varepsilon\,, \tag{S136}$$

where $\psi_i := |\psi_i\rangle\langle\psi_i|$ and $\psi_j := |\psi_j\rangle\langle\psi_j|$ (as depicted in Fig. S2 A 1). Thanks to Lemma S14, any tomography algorithm that learns states from the set $\mathcal{S}_{\text{pure}}(n,k,N_{\text{phot}})$ with accuracy $\le \varepsilon$ in trace distance and failure probability $\le \delta$ must use a number of state copies N satisfying:

$$\chi \ge (1-\delta)\log_2(M) - H_2(\delta)\,, \tag{S137}$$

where $\chi := S\left(\frac{1}{M}\sum_{j=1}^M \psi_j^{\otimes N}\right) - \frac{1}{M}\sum_{j=1}^M S(\psi_j^{\otimes N})$ is the Holevo information, and $S(\cdot)$ is the von Neumann entropy. Note that the latter quantity can be upper bounded as

$$\chi = S\left(\frac{1}{M}\sum_{j=1}^M \psi_j^{\otimes N}\right) - \frac{1}{M}\sum_{j=1}^M S(\psi_j^{\otimes N}) \le S\left(\frac{1}{M}\sum_{j=1}^M \psi_j^{\otimes N}\right) \le nNg(N_{\text{phot}})\,, \tag{S138}$$

where in the last inequality we have exploited the fact that the von Neumann entropy of a state with a fixed mean photon number can be upper bounded in terms of the bosonic entropy function $g(\cdot)$ (see Lemma S9). Specifically, note that the mean total photon number of the nN-mode state $\frac{1}{M}\sum_{j=1}^{M}\psi_j^{\otimes N}$ can be upper bounded as

$$\mathrm{Tr}\left[\hat{N}_{nN}\left(\frac{1}{M}\sum_{j=1}^{M}\psi_j^{\otimes N}\right)\right]=N\frac{1}{M}\sum_{j=1}^{M}\mathrm{Tr}[\hat{N}_n\psi_j]\le N\frac{1}{M}\sum_{j=1}^{M}\left(\mathrm{Tr}[\hat{N}_n^k\psi_j]\right)^{1/k}\le nN N_{\text{phot}}\,, \tag{S139}$$

where in the first inequality we have used that $\mathrm{Tr}[\hat{N}_n\psi_j]=\mathrm{Tr}[(\hat{N}_n^k)^{\frac{1}{k}}\psi_j]$ and exploited the concavity of $x\mapsto x^{1/k}$ for $x>0, k\in\mathbb{N}$ (see Eq. (S7)), while in the last inequality we have exploited that $\psi_j\in\mathcal{S}_{\text{pure}}(n,k,N_{\text{phot}})$. Hence, Lemma S9 establishes that

$$S\left(\frac{1}{M}\sum_{j=1}^{M}\psi_j^{\otimes N}\right)\le nNg(N_{\text{phot}})\,. \tag{S140}$$

By putting together (S137) and (S138), we deduce that, if such M exists, then the number of state copies N required for tomography of an unknown state belonging to $\mathcal{S}_{\text{pure}}(n,k,N_{\text{phot}})$ has to be at least

$$N\ge\frac{(1-\delta)\log_2(M)-H_2(\delta)}{ng(N_{\text{phot}})}\,. \tag{S141}$$

Hence, in order to get a good lower bound on N, we need to find a large value of M such that there exist M states

$$\{\psi_1,\psi_2,\dots,\psi_M\}\subseteq\mathcal{S}_{\text{pure}}(n,k,N_{\text{phot}}) \tag{S142}$$

for which it holds that $\frac{1}{2}\|\psi_i-\psi_j\|_1>2\varepsilon$ for all $i\neq j\in[M]$. To this end, let us make a suitable construction of such M states.

Let $m\in\mathbb{N}$ such that $m\ge nN_{\text{phot}}$ which will be fixed later. Let us define the finite-dimensional Hilbert space

$$\mathcal{H}_m:=\left\{|\phi\rangle\in\mathrm{span}\left(|\mathbf{k}\rangle:\ \mathbf{k}\in\mathbb{N}^n\setminus\{0\}\,,\sum_{i=1}^{n}k_i\le m\right)\,:\,\langle\phi|\phi\rangle=1\right\}\,, \tag{S143}$$

spanned by all the n-mode Fock states, apart from the n-mode vacuum state, with total number of photons less than m, where $|\mathbf{k}\rangle$ is the n-mode Fock state defined by $|\mathbf{k}\rangle=|k_1\rangle\otimes|k_2\rangle\otimes\ldots\otimes|k_n\rangle$.

Given a state vector $|\phi\rangle\in\mathcal{H}_m$, we can define a corresponding state vector $|\psi_\phi\rangle\in\mathcal{S}_{\text{pure}}(n,k,N_{\text{phot}})$ as

$$|\psi_\phi\rangle:=\sqrt{1-\left(\frac{nN_{\text{phot}}}{m}\right)^k}\,|0\rangle^{\otimes n}+\sqrt{\left(\frac{nN_{\text{phot}}}{m}\right)^k}\,|\phi\rangle\,. \tag{S144}$$

Indeed, the k-moment of $|\psi_\phi\rangle$ can be upper bounded as

$$\mathrm{Tr}[\hat{N}_n^k\,|\psi_\phi\rangle\!\langle\psi_\phi|]=\frac{(nN_{\text{phot}})^k}{m^k}\,\mathrm{Tr}[\hat{N}_n^k\,|\phi\rangle\!\langle\phi|]\le\frac{(nN_{\text{phot}})^k}{m^k}m^k=(nN_{\text{phot}})^k\,, \tag{S145}$$

and thus $|\psi_\phi\rangle\in\mathcal{S}_{\text{pure}}(n,k,N_{\text{phot}})$. Moreover, as we show below in (S154), the following inequality holds

$$\frac{1}{2}\left\||\psi_\phi\rangle\!\langle\psi_\phi|-|\psi_{\tilde{\phi}}\rangle\!\langle\psi_{\tilde{\phi}}|\right\|_1\ge\sqrt{\frac{(nN_{\text{phot}})^k}{8m^k}}\left\||\phi\rangle\!\langle\phi|-|\tilde{\phi}\rangle\!\langle\tilde{\phi}|\right\|_1 \tag{S146}$$

for all $|\phi\rangle\,,|\tilde{\phi}\rangle\in\mathcal{H}_m$. Hence, by choosing

$$m:=\left\lfloor\frac{nN_{\text{phot}}}{(12\varepsilon)^{2/k}}\right\rfloor\,, \tag{S147}$$

one can check that for all $|\phi\rangle\,,|\tilde{\phi}\rangle\in\mathcal{H}_m$ such that

$$\left\||\phi\rangle\!\langle\phi|-|\tilde{\phi}\rangle\!\langle\tilde{\phi}|\right\|_1\ge\frac{1}{2}\,, \tag{S148}$$

the inequality in (S146) guarantees that

$$\frac{1}{2}\left\| |\psi_\phi\rangle\langle\psi_\phi| - |\psi_{\tilde{\phi}}\rangle\langle\psi_{\tilde{\phi}}| \right\|_1 \geq 2\varepsilon\,. \tag{S149}$$

Therefore, let us find $M \in \mathbb{N}$ such that there exist M states $\{|\phi_1\rangle, |\phi_2\rangle, \dots, |\phi_M\rangle\} \subseteq \mathcal{H}_m$ such that $\| |\phi_i\rangle\langle\phi_i| - |\phi_j\rangle\langle\phi_j| \|_1 \geq \frac{1}{2}$ for all $i \neq j \in [M]$. By definition of packing number (see Definition 2), such M can be chosen to be the $\frac{1}{2}$-packing number of $\mathcal{H}_m$ with respect to the trace norm, i.e., $M \coloneqq \mathcal{P}\big(\mathcal{H}_m, \|\cdot\|_1, \frac{1}{2}\big)$. We can find a simple lower bound on M as

$$M = \mathcal{P}\left(\mathcal{H}_m, \|\cdot\|_1, \frac{1}{2}\right) \overset{\text{(i)}}{\geq} \frac{1}{8\pi} 2^{2(\dim \mathcal{H}_m - 1)} \overset{\text{(ii)}}{\geq} \frac{1}{8\pi} 2^{2[\binom{n+m}{n} - 2]} \overset{\text{(iii)}}{\geq} \frac{1}{8\pi} 2^{2[(\frac{m}{n})^n - 1]} = \frac{1}{32\pi} 2^{2(\frac{m}{n})^n}\,. \tag{S150}$$

Here, in (i), we exploit Lemma S18; in (ii), we observed that the dimension of $\mathcal{H}_m$ is given by

$$\dim \mathcal{H}_m = \left| \left\{ \mathbf{k} \in \mathbb{N}^n \setminus \{0\}\,, \quad \sum_{i=1}^n k_i \leq m \right\} \right| = \binom{m+n}{n} - 1\,, \tag{S151}$$

where the last equality follows from a standard combinatorial argument; in (iii), we just exploited that $\binom{m+n}{n} \geq (\frac{m+n}{n})^n \geq (\frac{m}{n})^n + 1$. Consequently, (S141) implies that

$$N \geq \frac{(1-\delta)2(\frac{m}{n})^n - (1-\delta)\log_2(32\pi) - H_2(\delta)}{n g(N_{\text{phot}})}\,. \tag{S152}$$

Finally, by exploiting the definition of m in (S147) we conclude that

$$N \geq \frac{2(1-\delta)\left(\frac{N_{\text{phot}}}{(12\varepsilon)^{2/k}} - \frac{1}{n}\right)^n - (1-\delta)\log_2(32\pi) - H_2(\delta)}{n g(N_{\text{phot}})}\,. \tag{S153}$$

We are just left to show (S146), which follows from

$$\begin{aligned}
\| \, |\psi_\phi\rangle\langle\psi_\phi| - |\psi_{\tilde{\phi}}\rangle\langle\psi_{\tilde{\phi}}| \, \|_1 &= 2\sqrt{1 - |\, \langle\psi_\phi|\psi_{\tilde{\phi}}\rangle \,|^2} \\
&= 2\sqrt{1 - \left| \left(1 - \frac{(nN_{\text{phot}})^k}{m^k}\right) + \frac{(nN_{\text{phot}})^k}{m^k} \langle\phi|\tilde{\phi}\rangle \right|^2} \\
&\geq 2\sqrt{1 - \left(1 - \frac{(nN_{\text{phot}})^k}{m^k} + \frac{(nN_{\text{phot}})^k}{m^k} \left|\langle\phi|\tilde{\phi}\rangle\right| \right)^2} \\
&= 2\sqrt{2\frac{(nN_{\text{phot}})^k}{m^k}\left(1 - \left|\langle\phi|\tilde{\phi}\rangle\right|\right) - \left(\frac{(nN_{\text{phot}})^k}{m^k}\left(1 - \left|\langle\phi|\tilde{\phi}\rangle\right|\right)\right)^2} \\
&\geq 2\sqrt{\frac{(nN_{\text{phot}})^k}{m^k}\left(1 - \left|\langle\phi|\tilde{\phi}\rangle\right|\right)} \\
&\geq \sqrt{2}\sqrt{\frac{(nN_{\text{phot}})^k}{m^k}}\sqrt{\left(1 - \left|\langle\phi|\tilde{\phi}\rangle\right|\right)\left(1 + \left|\langle\phi|\tilde{\phi}\rangle\right|\right)} \\
&= \sqrt{2}\sqrt{\frac{(nN_{\text{phot}})^k}{m^k}}\sqrt{\left(1 - \left|\langle\phi|\tilde{\phi}\rangle\right|^2\right)} \\
&= \sqrt{\frac{(nN_{\text{phot}})^k}{2m^k}} \left\| |\phi\rangle\langle\phi| - |\tilde{\phi}\rangle\langle\tilde{\phi}| \right\|_1\,,
\end{aligned} \tag{S154}$$

which concludes the proof. □

In particular, in the single-mode case, i.e., $n = 1$, the number of state copies N has to satisfy

$$\begin{aligned}
N &\geq \frac{1}{g(N_{\text{phot}})}\left[2(1-\delta)\left(\frac{N_{\text{phot}}}{(12\varepsilon)^{2/k}} - 1\right) - (1-\delta)\log_2(32\pi) - H_2(\delta)\right] \\
&= \Theta\left(\frac{N_{\text{phot}}}{\varepsilon^{2/k}}\right)\,.
\end{aligned} \tag{S155}$$

2. *Lower bound for mixed state tomography*

In this subsection, we establish a theorem concerning the number of copies necessary for learning moment-constrained mixed states. The proof strategy follows a similar logic to the one used previously for the pure state case.

Theorem S27 (Lower bound on sample complexity for moment-constrained mixed states)**.** *Let us consider a tomography algorithm that learns, within a trace distance $\le \varepsilon$ and failure probability $\le \delta$, an arbitrary state belonging to the set*

$$\mathcal{S}(n,k,N_{phot}) := \left\{ \rho \in \mathcal{D}(L^2(\mathbb{R}^n)), \quad \left(\mathrm{Tr}\left[\left(\hat{N}_n\right)^k \rho\right]\right)^{1/k} \le nN_{phot} \right\} \tag{S156}$$

of n-mode (possibly mixed) states with bounded k-moment. Then, such a tomography algorithm must use a number of state copies N satisfying

$$\begin{aligned} N &\ge \frac{1}{2ng(N_{phot})}\left[(1-\delta)\left(\frac{N_{phot}}{(16\varepsilon)^{1/k}} - \frac{1}{n}\right)^{2n} - \frac{1}{2}(1-\delta) - 2H_2(\delta)\right] \\ &= \Theta\left(\frac{N_{phot}}{\varepsilon^{1/k}}\right)^{2n} . \end{aligned} \tag{S157}$$

Here, $g(x) := (x+1)\log_2(x+1) - x\log_2 x$ is the bosonic entropy, and $H_2(x) := -x\log_2 x - (1-x)\log_2(1-x)$ is the binary entropy.

Proof. Let $M \in \mathbb{N}$ be such that there exist M states

$$\{\rho_1, \rho_2, \ldots, \rho_M\} \subseteq \mathcal{S}(n,k,N_{\text{phot}}) \tag{S158}$$

such that for every $i \neq j \in [M]$ it holds that

$$\frac{1}{2}\|\rho_i - \rho_j\|_1 > 2\varepsilon . \tag{S159}$$

By applying Lemma S14 in the exact same way as we did in the proof of Theorem S26, we deduce that the number of state copies N required for tomography of an unknown state belonging to $\mathcal{S}(n,k,N_{\text{phot}})$ has to be at least

$$N \ge \frac{(1-\delta)\log_2(M) - H_2(\delta)}{ng(N_{\text{phot}})} . \tag{S160}$$

Let $m \in \mathbb{N}$ such that $m \ge nN_{\text{phot}}$. Let $\mathcal{H}_m$ be the Hilbert space, defined in (S143), spanned by all the n-mode Fock states, apart from the n-mode vacuum state, with total number of photons less than m. Moreover, let $\mathcal{D}(\mathcal{H}_m)$ be the set of density operators on $\mathcal{H}_m$. Given a state $\rho \in \mathcal{D}(\mathcal{H}_m)$, we can define a corresponding state $\sigma_\rho \in \mathcal{S}(n,k,N_{\text{phot}})$ as

$$\sigma_\rho := \left[1 - \left(\frac{nN_{\text{phot}}}{m}\right)^k\right] |0\rangle\langle 0|^{\otimes n} + \left(\frac{nN_{\text{phot}}}{m}\right)^k \rho . \tag{S161}$$

Indeed, the k-moment of $|\psi_\phi\rangle$ can be upper bounded as

$$\mathrm{Tr}[\hat{N}_n^k \sigma_\rho] = \left(\frac{nN_{\text{phot}}}{m}\right)^k \mathrm{Tr}[\hat{N}_n^k \rho] \le \frac{(nN_{\text{phot}})^k}{m^k} m^k = (nN_{\text{phot}})^k , \tag{S162}$$

and thus $\sigma_\rho \in \mathcal{S}(n,k,N_{\text{phot}})$. Moreover, it holds that

$$\|\sigma_\rho - \sigma_{\rho'}\|_1 = \left(\frac{nN_{\text{phot}}}{m}\right)^k \|\rho - \rho'\|_1 \tag{S163}$$

for all $\rho, \rho' \in \mathcal{D}(\mathcal{H}_m)$. Hence, by choosing

$$m := \left\lfloor \frac{nN_{\text{phot}}}{(16\varepsilon)^{1/k}} \right\rfloor , \tag{S164}$$

one can check that for all $\rho, \rho' \in \mathcal{D}(\mathcal{H}_m)$ such that

$$\|\rho - \rho'\|_1 \geq \frac{1}{4}, \tag{S165}$$

the relation in (S163) guarantees that

$$\frac{1}{2}\|\sigma_\rho - \sigma_{\rho'}\|_1 \geq 2\varepsilon . \tag{S166}$$

Therefore, let us find $M \in \mathbb{N}$ such that there exist M states $\{\rho_1, \rho_2, \ldots, \rho_M\} \subseteq \mathcal{D}(\mathcal{H}_m)$ such that $\|\rho_i - \rho_j\|_1 \geq \frac{1}{4}$ for all $i \neq j \in [M]$. By definition of packing number (see Definition 2), such M can be chosen to be the $\frac{1}{4}$-packing number of $\mathcal{D}(\mathcal{H}_m)$ with respect to the trace norm

$$M := \mathcal{P}\left(\mathcal{D}(\mathcal{H}_m), \|\cdot\|_1, \frac{1}{4}\right) . \tag{S167}$$

We can find a simple lower bound on M as

$$\begin{aligned} M &= \mathcal{P}\left(\mathcal{D}(\mathcal{H}_m), \|\cdot\|_1, \frac{1}{4}\right) \\ &\overset{\text{(i)}}{\geq} 2^{\frac{(\dim \mathcal{H}_m - 1)^2}{2}} \\ &\overset{\text{(ii)}}{\geq} 2^{\frac{\left(\binom{n+m}{n} - 2\right)^2}{2}} \\ &\overset{\text{(iii)}}{\geq} 2^{\frac{1}{2}\left(\left(\frac{m}{n}\right)^{2n} - 1\right)} \\ &= \frac{1}{\sqrt{2}} 2^{\frac{1}{2}\left(\frac{m}{n}\right)^{2n}} . \end{aligned} \tag{S168}$$

Here, in (i), we exploit Corollary S21; in (ii), we have exploited (S151); in (iii), we have used that $\binom{m+n}{n} \geq \left(\frac{m+n}{n}\right)^n \geq \left(\frac{m}{n}\right)^n + 1$. Consequently, (S160) implies that

$$N \geq \frac{\frac{1-\delta}{2}\left(\frac{m}{n}\right)^{2n} - (1-\delta)\log_2(\sqrt{2}) - H_2(\delta)}{ng(N_{\text{phot}})} . \tag{S169}$$

Finally, by exploiting the definition of m in (S147) we conclude that

$$N \geq \frac{1}{2ng(N_{\text{phot}})}\left[(1-\delta)\left(\frac{N_{\text{phot}}}{(16\varepsilon)^{1/k}} - \frac{1}{n}\right)^{2n} - \frac{1}{2}(1-\delta) - 2H_2(\delta)\right] . \tag{S170}$$

□

In particular, in the single-mode case, i.e., $n = 1$, the number of state copies N has to satisfy

$$\begin{aligned} N &\geq \frac{1}{2g(N_{\text{phot}})}\left[(1-\delta)\left(\frac{N_{\text{phot}}}{(16\varepsilon)^{1/k}} - 1\right)^2 - \frac{1}{2}(1-\delta) - 2H_2(\delta)\right] \\ &= \Theta\left(\frac{N_{\text{phot}}^2}{\varepsilon^{2/k}}\right) . \end{aligned} \tag{S171}$$

B. Sample complexity upper bounds

In this subsection, we present a tomography algorithm aimed at learning a classical description of an unknown n-mode k-th moment-constrained state. We analyse both pure and mixed scenarios. The tomography algorithm involves two main steps: first, projecting onto a finite-dimensional subspace, and second, applying a known tomography algorithm specifically designed for finite-dimensional systems.

The subsection is organised as follows. In Subsubsection S2 B 1, we establish useful properties of moment-constrained states. Specifically, we demonstrate how to approximate a moment-constrained state using a finite-dimensional state with bounded effective rank. In Subsubsection S2 B 2, we compare our approximation with those of other works, highlighting the improvements achieved. Subsubsection S2 B 3 outlines the precise performances of an optimal tomography algorithm applicable to finite-dimensional systems. Finally, in Subsubsection S2 B 4, we present the tomography algorithms for moment-constrained pure and mixed states and establish their correctness. We denote the Euler's Constant as e throughout this subsection.

1. Properties of moment-constrained states: approximation with finite-dimensional and finite-rank states

The following lemma establishes that k-th moment-constrained states can be effectively approximated by finite-dimensional states.

Lemma S28 (Effective dimension of moment-constrained states). *Let $\varepsilon \in (0,1)$ and let ρ be an n-mode state with k-moment bounded as $(\mathrm{Tr}[\hat{N}_n^k\rho])^{1/k} \le nN_{phot}$. Then, ρ is ε-close in trace distance to a state supported on a space of dimension $\Theta\Big(\frac{eN_{phot}}{\varepsilon^{2/k}}\Big)^n$.*

More specifically, let $\mathcal{H}_m$ be the Hilbert space spanned by all the n-mode Fock states with total photon number less than m:

$$\mathcal{H}_m := \mathrm{Span}\left\{ |k_1\rangle \otimes |k_2\rangle \otimes \ldots \otimes |k_n\rangle : \; k_1, k_2, \ldots, k_n \in \mathbb{N},\; \sum_{i=1}^n k_i \le m \right\} . \tag{S172}$$

Let $m := \left\lceil \frac{nN_{phot}}{\varepsilon^{2/k}} \right\rceil$. Define the state $\rho_{d_{eff}} := \Pi_m\rho\,\Pi_m / \mathrm{Tr}[\Pi_m\rho\,\Pi_m]$ as the projection of ρ onto $\mathcal{H}_m$, where Π_m is the projector onto $\mathcal{H}_m$, whose dimension satisfies

$$d_{eff} := \dim \mathcal{H}_m = \binom{m+n}{n} \le \left(\frac{eN_{phot}}{\varepsilon^{2/k}} + 2e\right)^n = \Theta\left(\frac{eN_{phot}}{\varepsilon^{2/k}}\right)^n . \tag{S173}$$

Then, $\rho_{d_{eff}}$ is ε-close in trace distance to ρ in that

$$\frac{1}{2}\left\|\rho - \rho_{d_{eff}}\right\|_1 \le \varepsilon . \tag{S174}$$

Proof. First, observe that the dimension $d_{\text{eff}} = \dim \mathcal{H}_m$ is given by

$$\begin{aligned} \dim \mathcal{H}_m &= \left| \left\{ k_1, k_2, \ldots, k_n \in \mathbb{N} , \quad \sum_{i=1}^n k_i \le m \right\} \right| \\ &\overset{\text{(i)}}{=} \binom{m+n}{n} \\ &\overset{\text{(ii)}}{\le} \left(\frac{e(m+n)}{n}\right)^n \\ &\overset{\text{(iii)}}{\le} \left(\frac{eN_{\text{phot}}}{\varepsilon^{2/k}} + 2e\right)^n . \end{aligned} \tag{S175}$$

Here, in (i), we have exploited a standard combinatorial argument; in (ii), we have used the fact that $\binom{a}{b} \le (\frac{ea}{b})^b$ for all $a, b \in \mathbb{N}$; in (iii), we have used the definition of $m := \left\lceil \frac{nN_{\text{phot}}}{\varepsilon^{2/k}} \right\rceil$. Moreover, let us note that

$$\begin{aligned} \mathrm{Tr}[(\mathbb{1} - \Pi_m)\rho] &\overset{\text{(iv)}}{\le} \frac{1}{m^k} \mathrm{Tr}\left[\hat{N}_n^k \rho\right] \\ &\overset{\text{(v)}}{\le} \frac{(nN_{\text{phot}})^k}{m^k} \\ &\overset{\text{(vi)}}{\le} \varepsilon^2 , \end{aligned} \tag{S176}$$

where: (iv) follows by the operator inequality $m^k(\mathbb{1} - \Pi_m) \le \hat{N}_n^k$; (v) follows by the hypothesis on the k-th moment of ρ; in (vi) we have used again the definition of m. Finally, the trace distance between ρ and the projected state $\rho_{d_{\text{eff}}}$ satisfies

$$\begin{aligned} \frac{1}{2}\left\|\rho_{d_{\text{eff}}} - \rho\right\|_1 &= \frac{1}{2}\left\| \frac{\Pi_m\rho\,\Pi_m}{\mathrm{Tr}[\Pi_m\rho\,\Pi_m]} - \rho \right\|_1 \\ &\overset{\text{(vii)}}{\le} \sqrt{\mathrm{Tr}[(\mathbb{1} - \Pi_m)\rho]} \\ &\le \varepsilon , \end{aligned} \tag{S177}$$

where in (vii) we have used the *gentle measurement lemma* [? , Lemma 6.15]. □

We now mention a lemma which will be useful consequently.

Lemma S29 (Infinite-dimensional Schur-Horn theorem ([**?**], Proposition 6.4))**.** *Let $\mathcal{H}$ be a Hilbert space. Let $N \in \mathbb{N}$ and $M \geq 0$ be a semi-definite operator on $\mathcal{H}$. For any set of orthonormal vectors $\{|v_n\rangle \in \mathcal{H} : n \in \{0, 1, \ldots, N-1\}\}$, it holds that*

$$\sum_{n=0}^{N-1} \lambda_n \geq \sum_{n=0}^{N-1} \langle v_n|M|v_n\rangle, \tag{S178}$$

where $\lambda_0 \geq \lambda_1 \geq \cdots \geq \lambda_{N-1}$ are the N largest eigenvalues of M.

In the following lemma, we establish that any moment-constrained state is close in trace distance to a state with bounded rank.

Lemma S30 (Effective rank of moment-constrained states)**.** *Let $\varepsilon \in (0,1)$. Consider an n-mode state ρ satisfying the k-th moment constraint $(\mathrm{Tr}[\rho \hat{N}_n^k])^{1/k} \leq nN_{phot}$. Then, ρ is ε-close in trace distance to a state $\rho_{r_{eff}}$ with rank $r_{eff} = \Theta\left(\frac{eN_{phot}}{\varepsilon^{1/k}}\right)^n$.*

More specifically, there exists a state $\rho_{r_{eff}}$ with rank r_{eff}, where

$$r_{eff} := \mathrm{rank}(\rho_{r_{eff}}) \leq \left(\frac{eN_{phot}}{\varepsilon^{1/k}} + 2e\right)^n = \Theta\left(\frac{eN_{phot}}{\varepsilon^{1/k}}\right)^n, \tag{S179}$$

such that $\frac{1}{2}\|\rho - \rho_{r_{eff}}\|_1 \leq \varepsilon$.

Proof. Let $\rho = \sum_{j=1}^{\infty} \lambda_j^{\downarrow} \psi_j$ be the the spectral decomposition of ρ, with $(\lambda_j^{\downarrow})_{j \in \mathbb{N}_+}$ being its eigenvalues ordered in decreasing order with respect to j. For each $r_{\mathrm{eff}} \in \mathbb{N}$, let us define the operator

$$\Theta_{r_{\mathrm{eff}}} := \sum_{j=1}^{r_{\mathrm{eff}}} \lambda_j^{\downarrow} \psi_j \tag{S180}$$

which has rank equal to r_{eff}. Note that the *infinite-dimensional Schur Horn theorem* (Lemma S29) implies that for any r_{eff}-rank projector $\Pi^{(r_{\mathrm{eff}})}$ it holds that

$$\|\rho - \Theta_{r_{\mathrm{eff}}}\|_1 = \sum_{j=r_{\mathrm{eff}}+1}^{\infty} \lambda_j^{\downarrow} \leq \mathrm{Tr}[(\mathbb{1} - \Pi^{(r_{\mathrm{eff}})})\rho]\,. \tag{S181}$$

Now, let us choose $\Pi^{(r_{\mathrm{eff}})}$ to be the projector $\Pi_{m'}$ onto the subspace $\mathcal{H}_{m'}$, defined in (S172), spanned by the n-mode Fock states with total photon number at most m', with $m' := \left\lceil \frac{nN_{\mathrm{phot}}}{\varepsilon^{1/k}} \right\rceil$. Hence, we have that

$$r_{\mathrm{eff}} := \mathrm{Tr}\,\Pi_{m'} = \binom{m'+n}{n} \leq \left(\frac{e(m'+n)}{n}\right)^n \leq \left(\frac{eN_{\mathrm{phot}}}{\varepsilon^{1/k}} + 2e\right)^n. \tag{S182}$$

Thanks to (S176), we have that

$$\mathrm{Tr}[(\mathbb{1} - \Pi_{m'})\rho] \leq \left(\frac{nN_{\mathrm{phot}}}{m'}\right)^k \leq \varepsilon\,, \tag{S183}$$

and thus it holds that $\mathrm{Tr}[(\mathbb{1} - \Pi^{(r_{\mathrm{eff}})})\rho] \leq \varepsilon$. Consequently, we deduce that $\|\rho - \Theta_{r_{\mathrm{eff}}}\|_1 \leq \varepsilon$. Therefore, by setting

$$\rho_{r_{\mathrm{eff}}} := \frac{\Theta_{r_{\mathrm{eff}}}}{\mathrm{Tr}\,\Theta_{r_{\mathrm{eff}}}} \tag{S184}$$

we have that

$$\begin{aligned}
\|\rho - \rho_{r_{\mathrm{eff}}}\|_1 &= \left\|\rho - \frac{\Theta_{r_{\mathrm{eff}}}}{\mathrm{Tr}\,\Theta_{r_{\mathrm{eff}}}}\right\|_1 \\
&\leq \|\rho - \Theta_{r_{\mathrm{eff}}}\|_1 + \left\|\Theta_{r_{\mathrm{eff}}} - \frac{\Theta_{r_{\mathrm{eff}}}}{\mathrm{Tr}\,\Theta_{r_{\mathrm{eff}}}}\right\|_1 \\
&= \|\rho - \Theta_{r_{\mathrm{eff}}}\|_1 + \left|1 - \frac{1}{\mathrm{Tr}\,\Theta_{r_{\mathrm{eff}}}}\right| \mathrm{Tr}\,\Theta_{r_{\mathrm{eff}}} \\
&\leq \varepsilon + (1 - \mathrm{Tr}\,\Theta_{r_{\mathrm{eff}}}) \\
&\leq \varepsilon + \sum_{j=r_{\mathrm{eff}}+1}^{\infty} \lambda_j^{\downarrow} \\
&\leq 2\varepsilon\,.
\end{aligned} \tag{S185}$$

□

We have demonstrated that an n-mode k-th moment-constrained state is ε-close in trace distance to a qudit state residing in a known Hilbert space of dimension $d_{\text{eff}} \leq \left(\frac{eN_{\text{phot}}}{\varepsilon^{2/k}} + 2e\right)^n$. Additionally, we have shown that this moment-constrained state is also ε-close in trace distance to a state with rank $r_{\text{eff}} \leq \left(\frac{eN_{\text{phot}}}{\varepsilon^{1/k}} + 2e\right)^n$.

Now, we combine the previous two lemmas to show that any moment-constrained state is close to a finite-dimensional state which is close to a state with smaller rank.

Lemma S31 (Low rank and finite-dimensional approximation of moment-constrained states)**.** *Let $\varepsilon \in (0,1)$. Let ρ be an n-mode state with k-moment bounded as $\left(\mathrm{Tr}[\hat{N}_n^k \rho]\right)^{1/k} \leq nN_{phot}$. Let $\mathcal{H}_m$ be the Hilbert space, defined in (S172), spanned by all the n-mode Fock states with total photon number less than m with $m := \left\lceil \frac{nN_{phot}}{\varepsilon^{2/k}} \right\rceil$, with dimension*

$$\dim \mathcal{H}_m \leq \left(\frac{eN_{phot}}{\varepsilon^{2/k}} + 2e\right)^n = \Theta\left(\frac{N_{phot}}{\varepsilon^{2/k}}\right)^n . \tag{S186}$$

Let $\rho_{d_{\mathit{eff}}}$ be the projected state of ρ onto $\mathcal{H}_m$. Then, $\rho_{d_{\mathit{eff}}}$ is $\eta(\varepsilon)$-close in trace distance to a state on $\mathcal{H}_m$ with rank r_{eff} satisfying

$$r_{\mathit{eff}} \leq \left(\frac{N_{phot}}{\varepsilon^{1/k}} + 2e\right)^n = \Theta\left(\frac{N_{phot}}{\varepsilon^{1/k}}\right)^n , \tag{S187}$$

where we have defined $\eta(\varepsilon) := \left(2 + \frac{1}{\sqrt{1-\varepsilon}}\right)\varepsilon$.

Proof. Thanks to Lemma S30, there exists an n-mode state $\rho_{r_{\text{eff}}}$, with rank satisfying

$$\mathrm{rank}(\rho_{r_{\text{eff}}}) \leq \left(\frac{eN_{\text{phot}}}{\varepsilon^{1/k}} + 2e\right)^n = \Theta\left(\frac{eN_{\text{phot}}}{\varepsilon^{1/k}}\right)^n , \tag{S188}$$

which is ε-close in trace distance to ρ. Let ρ' be the projected state of $\rho_{r_{\text{eff}}}$ onto $\mathcal{H}_m$, that is,

$$\rho' := \frac{\Pi_m \rho_{r_{\text{eff}}} \Pi_m}{\mathrm{Tr}[\Pi_m \rho_{r_{\text{eff}}} \Pi_m]}, \tag{S189}$$

where Π_m is the projector onto $\mathcal{H}_m$. Clearly, ρ' is a state supported on $\mathcal{H}_m$. The chain of inequalities

$$\begin{aligned}
\frac{1}{2}\|\rho_{d_{\text{eff}}} - \rho'\|_1 &\overset{\text{(i)}}{\leq} \frac{1}{2}\|\rho_{d_{\text{eff}}} - \rho\|_1 + \frac{1}{2}\|\rho - \rho_{r_{\text{eff}}}\|_1 + \frac{1}{2}\|\rho_{r_{\text{eff}}} - \rho'\|_1 \\
&\overset{\text{(ii)}}{\leq} 2\varepsilon + \frac{1}{2}\|\rho_{r_{\text{eff}}} - \rho'\|_1 \\
&\overset{\text{(iii)}}{\leq} 2\varepsilon + \sqrt{\mathrm{Tr}[(\mathbb{1} - \Pi_m)\rho_{r_{\text{eff}}}]} \\
&\overset{\text{(iv)}}{\leq} 2\varepsilon + \frac{\varepsilon}{\sqrt{1-\varepsilon}} \\
&= \eta(\varepsilon)
\end{aligned} \tag{S190}$$

shows that ρ' is $\eta(\varepsilon)$-close in trace distance to $\rho_{d_{\text{eff}}}$. Here, in (i), we have used triangle inequality. In (ii), we have exploited Lemma S28 and Lemma S30 to ensure that $\frac{1}{2}\|\rho_{d_{\text{eff}}} - \rho\|_1 \leq \varepsilon$ and $\frac{1}{2}\|\rho - \rho_{r_{\text{eff}}}\|_1$, respectively. In (iii), we have used the *gentle measurement lemma* [? , Lemma 6.15]. In (iv), we have used that

$$\begin{aligned}
\mathrm{Tr}[(\mathbb{1} - \Pi_m)\rho_{r_{\text{eff}}}] &\overset{\text{(v)}}{=} \frac{1}{\mathrm{Tr}\,\Theta_{r_{\text{eff}}}} \mathrm{Tr}\left[(\mathbb{1} - \Pi_m)\Theta_{r_{\text{eff}}}\right] \\
&\overset{\text{(vi)}}{\leq} \frac{1}{1-\varepsilon} \mathrm{Tr}\left[(\mathbb{1} - \Pi_m)\Theta_{r_{\text{eff}}}\right] \\
&\overset{\text{(vii)}}{\leq} \frac{1}{1-\varepsilon} \mathrm{Tr}\left[(\mathbb{1} - \Pi_m)\rho\right] \\
&\overset{\text{(viii)}}{\leq} \frac{\varepsilon^2}{1-\varepsilon} .
\end{aligned} \tag{S191}$$

Here, in (v), we have used the definition $\rho_{r_{\text{eff}}} := \frac{\Theta_{r_{\text{eff}}}}{\operatorname{Tr}\Theta_{r_{\text{eff}}}}$ in (S184), where we recall that $\Theta_{r_{\text{eff}}}$ is defined in (S180) as $\Theta_{r_{\text{eff}}} := \sum_{j=1}^{r_{\text{eff}}} \lambda_j^{\downarrow}\psi_j$. In (vi), we have used that $\operatorname{Tr}\Theta_{r_{\text{eff}}} \geq 1-\varepsilon$, as it follows from the third and fifth line of (S185). In (vii), we have exploited that

$$\Theta_{r_{\text{eff}}} = \sum_{j=1}^{r_{\text{eff}}} \lambda_j^{\downarrow}\psi_j \leq \sum_{j=1}^{\infty} \lambda_j^{\downarrow}\psi_j = \rho\,. \tag{S192}$$

Finally, in (viii) we applied that (S176). □

2. Related works on approximating moment-constrained states

We demonstrated that an n-mode state ρ with k-moment bounded by N_{phot} can be approximated within trace distance precision ε by a projected state living in a subspace of *effective dimension* $d_{\text{eff}} = \lceil (eN_{\text{phot}}/\varepsilon^{2/k} + 2e)^n \rceil$ (Lemma S28). Naturally, the lower the effective dimension, the better the efficiency of the subsequent tomography algorithm for learning the projected state.

Previous works such as [**?** **?**] have established similar results for approximating n-mode moment-constrained states with a projected finite-dimensional state. However, our Lemma S28 improves upon these findings. Specifically, our effective dimension scales as $d_{\text{eff}} = \Theta(eN_{\text{phot}}/\varepsilon^{2/k})^n$, is in contrast to the scaling $d_{\text{eff}} = \Theta(nN_{\text{phot}}/\varepsilon^{2/k})^n$ obtained in [**?** **?**]. Unlike [**?** , Proposition 8], which involves approximating an n-mode state with an n-qudit state, our projection approximates an n-mode state with a single-qudit state. Furthermore, Ref. [**?** , Supplementary Note 1] exclusively focuses on the case $k = 1$ (energy-constrained states). While they use a projection similar to ours, they end up with an unfavourable scaling $d_{\text{eff}} = \Theta(nN_{\text{phot}}/\varepsilon)^n$ due to an overly crude upper bound on the effective dimension. In conclusion, our bound on the effective dimension of moment-constrained states represents to our knowledge the first achieved bound that is non-super-exponential in the number of modes.

3. Known optimal tomography algorithm for qudits

In this section, we review the guarantees of the optimal tomography algorithm known as the *truncated version of Keyl's algorithm*, which is detailed in Wright's PhD thesis [**?** , Theorem 1.4.13].

Lemma S32 (Truncated version of Keyl's algorithm [**?**])**.** *Let ρ be an unknown qudit state of dimension d, and let $\lambda_1^{\downarrow} \geq \lambda_2^{\downarrow} \geq \ldots \geq \lambda_d^{\downarrow}$ be its eigenvalues. For any $r \in [d]$, N copies of ρ are sufficient to build an estimator $\tilde{\rho}$ with rank r such that*

$$\mathbb{E}\|\rho - \tilde{\rho}\|_1 \leq \sum_{j=r+1}^{d} \lambda_j^{\downarrow} + 6\sqrt{\frac{rd}{N}}. \tag{S193}$$

We now introduce a lemma that later will be useful for expressing the sample complexity of the tomography algorithm.

Lemma S33. *Let ρ be a qudit state of dimension d (possibly $d = \infty$), and let $\lambda_1^{\downarrow} \geq \lambda_2^{\downarrow} \geq \ldots \geq \lambda_d^{\downarrow}$ be its eigenvalues ordered in decreasing order. For any state ρ_r with rank r, we have*

$$\sum_{j=r+1}^{d} \lambda_j^{\downarrow} \leq \frac{1}{2}\|\rho - \rho_r\|_1. \tag{S194}$$

Proof. Let Π_r be the projector onto the support of ρ_r. Note that

$$\begin{aligned}\sum_{j=r+1}^{d} \lambda_j^{\downarrow} &\overset{\text{(i)}}{\leq} \operatorname{Tr}[(\mathbb{1} - \Pi_r)\rho] \\ &\overset{\text{(ii)}}{=} \operatorname{Tr}[(\mathbb{1} - \Pi_r)(\rho - \rho_r)] \\ &\leq \max_{0 \leq E \leq \mathbb{1}} \operatorname{Tr}[E(\rho - \rho_r)] \\ &\overset{\text{(iii)}}{=} \frac{1}{2}\|\rho - \rho_r\|_1\,.\end{aligned} \tag{S195}$$

Here, in (i), we have used the *(infinite) dimensional Schur Horn theorem* (Lemma S29); in (ii), we just exploited the fact that $\Pi_r\rho_r = \rho_r\Pi_r = \rho_r$, which holds true since Π_r is the projector onto the support of ρ_r; in (iii), we just used the well-known variational characterisation of the trace distance in terms of POVM [**?**]. □

We are now ready to provide the precise performance guarantees of the algorithm [**?**].

Lemma S34 (Sample complexity of the optimal tomography algorithms for qudit systems)**.** *Let $\varepsilon, \delta \in (0,1)$. Let ρ be an (unknown) state of dimension d such that it is $\frac{\varepsilon}{3}$-close in trace distance to a state with rank r. Then, there exists a tomography algorithm such that given*

$$N \geq 2^{18} \frac{rd}{\varepsilon^2} \log\left(\frac{2}{\delta}\right) \tag{S196}$$

copies of ρ can build (a classical description of) an r-rank state estimator $\tilde{\rho}$ satisfying

$$\Pr\left[\frac{1}{2}\|\rho - \tilde{\rho}\|_1 \leq \varepsilon\right] \geq 1 - \delta\,. \tag{S197}$$

Proof. Thanks to Lemma S32, N' copies of ρ are sufficient in order to build an estimator $\tilde{\rho}$ (with rank r) such that

$$\mathbb{E}\|\rho - \tilde{\rho}\|_1 \leq \sum_{j=r+1}^{d} \lambda_j^{\downarrow} + 6\sqrt{\frac{rd}{N'}}\,, \tag{S198}$$

where $\lambda_1^{\downarrow} \geq \lambda_2^{\downarrow} \geq \ldots \geq \lambda_d^{\downarrow}$ are the eigenvalues of ρ. By assumption, there exists a state ρ_r with rank r such that $\frac{1}{2}\|\rho - \rho_r\|_1 \leq \frac{\varepsilon}{3}$. Consequently, Lemma S33 implies that $\sum_{j=r+1}^{d} \lambda_j^{\downarrow} \leq \frac{\varepsilon}{3}$. Hence, by choosing $N' := \left\lceil \frac{(18)^2 rd}{\varepsilon^2} \right\rceil$ and by applying Markov's inequality, we have that

$$\begin{aligned}
\Pr\left[\frac{1}{2}\|\rho - \tilde{\rho}\|_1 \geq \varepsilon\right] &\leq \frac{\mathbb{E}\|\rho - \tilde{\rho}\|_1}{2\varepsilon} \\
&\leq \frac{\sum_{j=r+1}^{d} \lambda_j^{\downarrow} + 6\sqrt{\frac{rd}{N'}}}{2\varepsilon} \\
&\leq \frac{1}{6} + \frac{3}{\varepsilon}\sqrt{\frac{rd}{N'}} \\
&\leq \frac{1}{3}\,.
\end{aligned} \tag{S199}$$

Consequently, we have proved that $N' = \left\lceil 324 \frac{rd}{\varepsilon^2} \right\rceil$ copies of ρ are sufficient in order to build an estimator $\tilde{\rho}$ such that $\Pr\left[\frac{1}{2}\|\rho - \tilde{\rho}\|_1 \leq \varepsilon\right] \geq \frac{2}{3}$. Now, we can apply the standard argument presented in Lemma S6 with $P_{\text{succ}} = \frac{2}{3}$ in order to enhance the probability of success from $\frac{2}{3}$ to $1 - \frac{\delta}{2}$. From such a lemma, by defining

$$m := \left\lceil \frac{2}{\left(1 - \frac{1}{2p_{\text{succ}}}\right)^2 p_{\text{succ}}} \log\left(\frac{2}{\delta}\right) \right\rceil = \left\lceil 48 \log\left(\frac{2}{\delta}\right) \right\rceil\,, \tag{S200}$$

it follows that a total of

$$mN' = \left\lceil 48 \log\left(\frac{2}{\delta}\right) \right\rceil \left\lceil 324 \frac{rd}{\varepsilon^2} \right\rceil \tag{S201}$$

copies of ρ suffices in order to build an estimator $\tilde{\rho}$ such that $\Pr\left[\frac{1}{2}\|\rho - \tilde{\rho}\|_1 \leq 3\varepsilon\right] \geq 1 - \frac{\delta}{2} \geq 1 - \delta$. Finally, by redefining $\varepsilon \mapsto \frac{\varepsilon}{3}$, we have that

$$\left\lceil 48 \log\left(\frac{2}{\delta}\right) \right\rceil \left\lceil 324 \frac{rd}{(\varepsilon/3)^2} \right\rceil \leq 2^{18} \frac{rd}{\varepsilon^2} \log\left(\frac{2}{\delta}\right)\,, \tag{S202}$$

and thus we conclude that $2^{18} \frac{rd}{\varepsilon^2} \log\left(\frac{2}{\delta}\right)$ copies of ρ are sufficient in order to build an estimator $\tilde{\rho}$ such that $\Pr\left[\frac{1}{2}\|\rho - \tilde{\rho}\|_1 \leq \varepsilon\right] \geq 1 - \delta$. ∎

One can prove that $\tilde{\Theta}\left(\frac{rd}{\varepsilon^2}\right)$ copies are not only sufficient (as established by the lemma above) but also necessary to perform quantum state tomography of a finite-dimensional quantum state [**? ?**].

Table S1. Tomography algorithm for n-mode k-th moment constrained states (pure and mixed). The algorithm for pure states and the one for mixed states are the same, apart from two distinctions: the number N of copies of the unknown state, and the specific input parameters provided to the subroutine called at Line 10 of the algorithm. For the mixed case the details of the algorithm are provided in the proof of Theorem S35; while for the pure case the details are reported in the proof of Theorem S36.

Input: Accuracy ε, failure probability δ, N copies of the unknown n-mode moment-constrained state ρ (as defined in Theorem S35 and Theorem S36 for mixed and pure states, respectively).
Output: A classical description of $\tilde{\rho}$, such that $\frac{1}{2}\|\tilde{\rho}-\rho\|_1 \le \varepsilon$ with probability at least $1-\delta$.

1: **for** $i \leftarrow 1$ **to** N **do**
2: Query a copy of ρ.
3: Perform the POVM $\{\Pi_m, \mathbb{1}-\Pi_m\}$, where Π_m is the projector onto the subspace $\mathcal{H}_m$, defined in Eq. (S172).
4: **if** the POVM outcome corresponds to $\mathbb{1}-\Pi_m$ **then**
5: Discard the post-measurement state.
6: **else**
7: Keep the post-measurement state.
8: **end if**
9: **end for**
10: Perform the full state tomography algorithm described in Lemma S34 on the kept copies of the post-measurement states, obtaining as output the classical description of a state $\tilde{\rho}$.
11: **return** $\tilde{\rho}$.

4. Tomography algorithm for moment-constrained mixed and pure states

In this subsection, we present a tomography algorithm to learn a classical description of an unknown n-mode moment-constrained state (pure and mixed), together with its sample complexity analysis. The tomography algorithm involves two main steps: first, projecting onto a finite-dimensional subspace (specifically, the subspace defined in Lemma S28), and second, applying a known tomography algorithm designed for finite-dimensional states (specifically, the algorithm described in Lemma S34). Table S1 presents the steps of our tomography algorithm. We begin with Theorem S35, which analyses the sample complexity of our tomography algorithm for moment-constrained mixed states. The basic idea of Theorem S35 is the following: the unknown moment-constrained mixed state is effectively a qudit state of dimension $d_{\text{eff}} = O\left(\frac{eN_{\text{phot}}}{\varepsilon^{2/k}}\right)^n$ and rank $r_{\text{eff}} = O\left(\frac{eN_{\text{phot}}}{\varepsilon^{1/k}}\right)^n$ (as established by Lemma S31), and thus the sample complexity of tomography is $O(r_{\text{eff}}\, d_{\text{eff}}) = O\left(\frac{N_{\text{phot}}}{\varepsilon^{3/(2k)}}\right)^{2n}$ (thanks to Lemma S34).

Theorem S35 (Learning moment-constrained mixed states)**.** *Let $\varepsilon, \delta \in (0,1)$. Let ρ be an n-mode state with k-moment bounded as $\left(\mathrm{Tr}[\hat{N}_n^k \rho]\right)^{1/k} \le nN_{phot}$. There exists a quantum algorithm that, utilising*

$$N = \left\lceil 2^{21} \frac{r_{\mathit{eff}}\, d_{\mathit{eff}}}{\varepsilon^2} \log\left(\frac{4}{\delta}\right)\right\rceil = O\left(\frac{N_{phot}}{\varepsilon^{3/(2k)}}\right)^{2n} \tag{S203}$$

copies of ρ, generates a classical representation of a d_{eff}-dimensional state $\tilde{\rho}$ with rank r_{eff} such that

$$\Pr\left[\frac{1}{2}\|\rho-\tilde{\rho}\|_1 \le \varepsilon\right] \ge 1-\delta\,, \tag{S204}$$

where $d_{\mathit{eff}} \le \left(\frac{eN_{phot}}{\varepsilon^{2/k}} + 2e\right)^n$ and $r_{\mathit{eff}} \le \left(\frac{eN_{phot}}{(\frac{\varepsilon}{20})^{1/k}} + 2e\right)^n$.

Proof. We aim to establish the correctness of the algorithm presented in Table S1. The algorithm queries $\lceil 2N' + 24\log\left(\frac{2}{\delta}\right)\rceil$ copies of ρ, where we will fix N' later. Let

$$m \coloneqq \left\lceil \frac{nN_{\text{phot}}}{(\varepsilon/2)^{2/k}} \right\rceil. \tag{S205}$$

On each copy, it executes the POVM $\{\Pi_m, \mathbb{1}-\Pi_m\}$, where $\Pi_m \coloneqq \sum_{\mathbf{m}:\, \sum_{i=1}^n m_i \le m} |\mathbf{m}\rangle\!\langle\mathbf{m}|$ is the projector onto the subspace $\mathcal{H}_m$, defined in Eq. (S172), spanned by the n-mode Fock states with total photon number at most m, with dimension (see

Lemma S28)

$$d_{\text{eff}} := \dim \mathcal{H}_m \leq \left(\frac{eN_{\text{phot}}}{\varepsilon^{2/k}} + 2e\right)^n = \Theta\left(\frac{eN_{\text{phot}}}{\varepsilon^{2/k}}\right)^n. \tag{S206}$$

The probability of obtaining the first POVM outcome is

$$\begin{aligned}\operatorname{Tr}[\Pi_m \rho] &= 1 - \operatorname{Tr}[(\mathbb{1} - \Pi_m)\rho] \\ &\geq 1 - \frac{1}{m^k}\operatorname{Tr}\left[\hat{N}_n^k \rho\right] \\ &\geq 1 - \frac{(nN_{\text{phot}})^k}{m^k} \\ &\geq 1 - \frac{\varepsilon^2}{4} \\ &\geq \frac{3}{4},\end{aligned} \tag{S207}$$

where the second step follows from the operator inequality $m^k(\mathbb{1} - \Pi_m) \leq \hat{N}_n^k$ and the third step follows from the hypothesis on the k-th moment of ρ.

Therefore, applying Lemma S5, we can assert that the algorithm, which uses a number of copies $\left\lceil 2N' + 24\log\left(\frac{2}{\delta}\right)\right\rceil$, with a probability $\geq 1 - \frac{\delta}{2}$ obtains at least N' copies of the post-measurement state $\rho_{d_{\text{eff}}} := \Pi_m \rho \Pi_m / \operatorname{Tr}[\Pi_m \rho]$. Due to Lemma S28, the post-measurement state satisfies

$$\frac{1}{2}\left\|\rho_{d_{\text{eff}}} - \rho\right\|_1 \leq \frac{\varepsilon}{2}. \tag{S208}$$

Moreover, Lemma S31 implies that $\rho_{d_{\text{eff}}}$ is $\eta(\varepsilon/20)$-close in trace distance to a state supported on $\mathcal{H}_m$ with rank r_{eff} satisfying

$$r_{\text{eff}} \leq \left(\frac{eN_{\text{phot}}}{(\frac{\varepsilon}{20})^{1/k}} + 2e\right)^n = \Theta\left(\frac{N_{\text{phot}}}{\varepsilon^{1/k}}\right)^n, \tag{S209}$$

where

$$\eta(\frac{\varepsilon}{20}) := \left(2 + \frac{1}{\sqrt{1 - \frac{\varepsilon}{20}}}\right)\frac{\varepsilon}{20} \leq \frac{\varepsilon}{6}. \tag{S210}$$

Thus, from Lemma S34, it follows that there exists a quantum algorithm that, utilising only

$$N' \geq 2^{18}\frac{r_{\text{eff}}\, d_{\text{eff}}}{(\varepsilon/2)^2}\log\left(\frac{4}{\delta}\right) \tag{S211}$$

copies of the post-measurement state $\rho_{d_{\text{eff}}}$, builds a classical description of an r_{eff}-rank state estimator $\tilde{\rho}$ such that, with a probability $\geq 1 - \frac{\delta}{2}$, it holds that

$$\frac{1}{2}\|\rho_{d_{\text{eff}}} - \tilde{\rho}\|_1 \leq \frac{\varepsilon}{2}. \tag{S212}$$

By the triangle inequality, we then conclude that $\frac{1}{2}\|\rho - \tilde{\rho}\|_1 \leq \varepsilon$. The total failure probability of the algorithm is $\leq \delta$ by applying a union bound. □

We now proceed with Theorem S36, which shows an upper bound on the sample complexity of tomography of moment-constrained pure states. The basic idea of Theorem S36 is the following: the unknown moment-constrained pure state is effectively a pure qudit state of dimension $d_{\text{eff}} = O\left(\frac{eN_{\text{phot}}}{\varepsilon^{2/k}}\right)^n$ (as established by Lemma S28), and thus the sample complexity of tomography is $O(d_{\text{eff}}) = O\left(\frac{N_{\text{phot}}}{\varepsilon^{2/k}}\right)^n$ (thanks to Lemma S34).

Theorem S36 (Learning moment-constrained pure states). *Let $\varepsilon, \delta \in (0,1)$. Consider an n-mode pure state ψ with its k-th moment bounded by $\left(\operatorname{Tr}[\hat{N}_n^k \psi]\right)^{1/k} \leq nN_{phot}$. There exists a quantum algorithm that, utilising*

$$N = \left\lceil 2^{21}\frac{d_{\text{eff}}}{\varepsilon^2}\log\left(\frac{4}{\delta}\right)\right\rceil = O\left(\frac{N_{phot}}{\varepsilon^{2/k}}\right)^n \tag{S213}$$

copies of ψ, generates a classical representation of a pure state $\tilde{\psi}$ such that

$$\Pr\left[\frac{1}{2}\|\psi-\tilde{\psi}\|_1 \le \varepsilon\right] \ge 1-\delta, \tag{S214}$$

where $d_{\mathit{eff}} \le \left(\frac{eN_{\mathit{phot}}}{\varepsilon^{2/k}}+2e\right)^n$.

Proof. We adopt the same notation used in Theorem S35. The algorithm is presented in Table S1, and the proof of its correctness follows similar steps to the previous mixed state case (Theorem S35). Specifically, the algorithm queries $\lceil 2N' + 24\log\left(\frac{2}{\delta}\right)\rceil$ copies of ψ and on each of them performs the POVM $\{\Pi_m, \mathbb{1}-\Pi_m\}$, where Π_m is the projector onto the subspace $\mathcal{H}_m$ (defined in Eq. (S172)). As in the proof of Theorem S35, with probability $\ge 1-\frac{\delta}{2}$, on at least N' copies the post-measurement state will be $\psi_{d_{\text{eff}}} := \Pi_m\psi\,\Pi_m/\operatorname{Tr}\left[\Pi_m\psi\right]$, satisfying $\frac{1}{2}\left\|\psi_{d_{\text{eff}}}-\psi\right\|_1 \le \frac{\varepsilon}{2}$.

By applying Lemma S34 with $r=1$, there exists a quantum algorithm that, utilising

$$N' \ge 2^{18}\frac{d_{\text{eff}}}{(\varepsilon/2)^2}\log\left(\frac{4}{\delta}\right), \tag{S215}$$

copies of the post-measurement state $\psi_{d_{\text{eff}}}$, builds a classical description of a pure state $\tilde{\psi}$ such that, with a probability $\ge 1-\frac{\delta}{2}$, it holds that

$$\frac{1}{2}\|\psi_{d_{\text{eff}}}-\tilde{\psi}\|_1 \le \frac{\varepsilon}{2}. \tag{S216}$$

By the triangle inequality, we conclude that $\frac{1}{2}\left\|\psi-\tilde{\psi}\right\|_1 \le \varepsilon$. The total failure probability of the algorithm is $\le \delta$ by applying a union bound. □

Remarkably, as a consequence of the sample-complexity lower bound proved in Theorem S26 and the sample-complexity upper bound proved in Theorem S36, we have the following.

Theorem S37 (Optimal sample complexity of tomography of moment-constrained pure states)**.** *Let ψ be an unknown pure n-mode state vector satisfying the k-th moment constraint $(\operatorname{Tr}[\hat{N}_n^k\rho])^{1/k} \le nN_{\mathit{phot}}$. Then, $\Theta\left(\frac{N_{\mathit{phot}}}{\varepsilon^{2/k}}\right)^n$ copies of ψ are necessary and sufficient to perform quantum state tomography with precision ε in trace distance.*

S3. TOMOGRAPHY OF BOSONIC GAUSSIAN STATES

Gaussian states play a crucial role in applications of quantum optics, such as quantum sensing, quantum communication, and optical quantum computing [**? ?**], and they form a small subset of the entire infinite-dimensional Hilbert space of continuous variable quantum states. In contrast to an arbitrary continuous variable quantum state, which is defined in terms of an infinite number of parameters, a Gaussian state is uniquely characterised by only a few parameters — specifically those present in its first moment and its covariance matrix. Indeed, it is well established that: 'In order to *know* a Gaussian state it is sufficient to *know* its first moment and its covariance matrix.' However, in practice, we never know the first moment and the covariance matrix *exactly*, but we can only have estimates of them, meaning that we can only approximately know the Gaussian state. Crucially, the *trace distance* between the exact quantum state and its approximation is the most meaningful figure of merit to measure of the error incurred in the approximation, due to the operational meaning of the trace distance given by the Holevo–Helstrom theorem [**? ?**]. It is thus a fundamental problem — yet never tackled before — of Gaussian quantum information to determine what is the error incurred in *trace distance* when estimating the first moment and covariance matrix of an unknown Gaussian state up to a precision ε. In this section, we address this fundamental problem, by finding upper and lower bounds on the trace distance between two arbitrary Gaussian states, determined by the norm distance of their covariance matrices and first moments. We present such bounds in the forthcoming Theorem S38.

One might be inclined to believe that there is a simple approach to solving this problem, involving first bounding the trace distance in terms of the fidelity and then employing the known formula for fidelity between Gaussian states [**?**]. Although this approach may seem promising at first glance, it is actually highly non-trivial because the expressions involved in the fidelity formula appear to be too complicated to allow the derivation of a bound in terms of the norm distance between the first moments and the covariance matrices.

Theorem S38 (Bounds on the trace distance between Gaussian states)**.** *Let ρ_1 and ρ_2 be n-mode Gaussian states satisfying the energy constraint* $\mathrm{Tr}[\rho_1\,\hat{N}_n]\le N$ *and* $\mathrm{Tr}[\rho_2\,\hat{N}_n]\le N$*. Let $\boldsymbol{m}_1$ and $\boldsymbol{m}_2$ be the first moments and let V_1 and V_2 be the covariance matrices of ρ_1 and ρ_2, respectively. The trace distance between ρ_1 and ρ_2 can be upper bounded as*

$$\frac{1}{2}\|\rho_1-\rho_2\|_1\le f(N)\left(\|\boldsymbol{m}_1-\boldsymbol{m}_2\|_2+\sqrt{2}\sqrt{\|V_1-V_2\|_1}\right)\,, \tag{S217}$$

where $f(N):=\frac{1}{\sqrt{2}}\left(\sqrt{N}+\sqrt{N+1}\right)$*. Moreover, it can be lower bounded as*

$$\frac{1}{2}\|\rho_1-\rho_2\|_1\ge\frac{1}{200}\max\left(\min\left(1,\frac{\|\boldsymbol{m}_1-\boldsymbol{m}_2\|_2}{\sqrt{h(N,n)}}\right),\min\left(1,\frac{\|V_1-V_2\|_2}{h(N,n)}\right)\right)\,, \tag{S218}$$

where $h(N,n):=4N+2n+1$.

Theorem S38 is not only a technical result of independent interest for the field of Gaussian quantum information, but also it answers the fundamental question: *if we approximate the first moment and covariance matrix of an unknown Gaussian state with precision ε, what is the resulting trace distance error on the state?* Theorem S38 establishes that if we make an error $O(\varepsilon)$ in approximating the first moment and the covariance matrix, then the trace-distance error that we make in approximating the unknown Gaussian state is *at most* $O(\sqrt{\varepsilon})$ and *at least* $O(\varepsilon)$.

The upper bound presented in Theorem S38 allows us to analyse the sample complexity of tomography of Gaussian states. In particular, we prove that tomography of (energy-constrained) Gaussian states is efficient, as there exists a tomography algorithm whose sample and time complexity scales polynomially in the number of modes. Notably, our result demonstrates that Gaussian states can be *efficiently* learned by estimating the first moment and the covariance matrix, a result that has been previously assumed but never rigorously proved in the literature.

Theorem S39 (Upper bound on the sample complexity of tomography of Gaussian states - informal version)**.** *A number* $O\!\left(\frac{n^7N_{\mathit{phot}}^4}{\varepsilon^4}\right)$ *of state copies are sufficient for quantum state tomography of n-mode Gaussian states ρ satisfying the energy constraint* $\mathrm{Tr}[\rho\hat{N}_n]\le nN_{\mathit{phot}}$*, where ε is the accuracy in trace distance.*

Notably, we can improve the trace distance upper bound in Theorem S38 if we assume one of the states, say ρ_2, to be a pure Gaussian state (interestingly, such an improved bound holds even if ρ_1 is not Gaussian).

Theorem S40 (Upper bound on the trace distance between a pure Gaussian state and a possibly non-Gaussian mixed state)**.** *Let $\psi:=|\psi\rangle\!\langle\psi|$ be a pure n-mode Gaussian state with first moment $\boldsymbol{m}(\psi)$ and second moment $V(\psi)$. Let ρ be an n-mode (possibly non-Gaussian) state with first moment $\boldsymbol{m}(\rho)$ and second moment $V(\rho)$. Assume that ρ and ψ satisfy the energy constraint* $\mathrm{Tr}[\rho\hat{N}_n]\le N$*. Then*

$$\frac{1}{2}\|\,\rho-|\psi\rangle\!\langle\psi|\,\|_1\le\sqrt{N+\frac{n}{2}}\sqrt{2\|\boldsymbol{m}(\rho)-\boldsymbol{m}(\psi)\|_2^2+\|V(\rho)-V(\psi)\|_\infty}\,. \tag{S219}$$

The improved bound presented in Theorem S40 allows us to analyse the sample complexity of tomography of pure Gaussian states.

Theorem S41 (Upper bound on the sample complexity of tomography of pure Gaussian states - informal version)**.** *A number* $O\left(\frac{n^5 N_{phot}^4}{\varepsilon^4}\right)$ *of state copies are sufficient for quantum state tomography of* n*-mode pure Gaussian states* ψ *satisfying the energy constraint* $\mathrm{Tr}[\psi \hat{N}_n] \leq nN_{phot}$*, where* ε *is the accuracy in trace distance.*

We have seen that learning unknown Gaussian states is efficient, with sample complexity scaling polynomially in the number of modes. But what if the unknown state is not exactly Gaussian? Is our tomography procedure robust against slight perturbations of the set of Gaussian states? These questions are crucial conceptually, given that experimental imperfections may transform Gaussian states into slightly-perturbed Gaussian states. In this section, we will also prove that *quantum state tomography of slightly-perturbed Gaussian states is efficient*. Technically, by 'slightly-perturbed Gaussian state' we mean that the minimum quantum relative entropy between the state and any Gaussian state is sufficiently small. The latter is a meaningful measure of 'non-Gaussianity', thanks to results from [**? ? ? ?**].

Our tomography algorithm for Gaussian states is feasible to realise in practice, as it only requires the estimation of the first moment and the covariance matrix of the unknown Gaussian state, tasks routinely accomplished in quantum optics laboratories through homodyne detection [**? ?**]. We stress that the non-trivial aspect of our tomography algorithm is that it comes with rigorous performance guarantees. That is, if the number of state copies is larger than a critical value (which scales polynomially in the number of modes), then the algorithm outputs a classical description of a Gaussian state which is guaranteed to be a 'good' approximation of the true state in trace distance with high probability.

This section is organised as follows:

- In Subsection S3 A, we prove upper and lower bounds on the trace distance between Gaussian states in terms of the norm distance between their first moments and their covariance matrices.
- In Subsection S3 B, we analyse the sample complexity of learning the first moment and the covariance matrix of unknown (possibly non-Gaussian) states.
- In Subsection S3 C, we put together all the above-mentioned results to analyse the sample complexity of tomography of (energy-constrained) Gaussian states both in the mixed-state and in the pure-state setting. We also how the second moment of the energy of a Gaussian state can be expressed in terms of its first moment and its covariance matrix, a result that turns out to be useful to obtain rigorous performance guarantees on tomography of energy-constrained Gaussian states. Finally, we show the our tomography procedure is robust under little perturbations from the set of Gaussian states, meaning that learning slightly-perturbed Gaussian states is efficient.

Note: Throughout this section, we sometimes represent energy constraints using the energy operator $\hat{E}_n$, such as $\mathrm{Tr}[\rho \hat{E}_n] \leq nE$, rather than the total photon number operator $\hat{N}_n$, like $\mathrm{Tr}[\rho \hat{N}_n] \leq nN_{\text{phot}}$. This simplifies the analysis without altering the sample complexity scaling, because of the identity $\hat{E}_n = \hat{N}_n + \frac{n}{2}\mathbb{1}$ that allows us to identify $E = N_{\text{phot}} + \frac{1}{2}$. For example, the upper bound on the sample-complexity of Gaussian-state tomography in Theorem S39, given by $O\left(\frac{n^7 N_{\text{phot}}^4}{\varepsilon^4}\right)$, can equivalently be expressed as $O\left(\frac{n^7 E^4}{\varepsilon^4}\right)$.

A. Bounds on the trace distance between Gaussian states

Possessing knowledge of the first moment and covariance matrix of a Gaussian state is sufficient to determine the state itself. However, when dealing with a finite number of copies of an unknown Gaussian state ρ, it is possible to obtain only estimates $\tilde{\mathbf{m}}$ and $\tilde{V}$ of its first moment $\mathbf{m}(\rho)$ and of its covariance matrix $V(\rho)$. Consequently, the resulting Gaussian state $\tilde{\rho}$ with first moment $\tilde{\mathbf{m}}$ and covariance matrix $\tilde{V}$ constitutes only an approximation of the true, unknown Gaussian state ρ. A natural question arises: What is the error incurred in the approximation $\tilde{\rho} \approx \rho$ in terms of the errors incurred in the approximations $\tilde{\mathbf{m}} \approx \mathbf{m}(\rho)$ and $V(\rho) \approx \tilde{V}$? The existing literature lacked such an error estimate, despite it being a natural question in Gaussian quantum information theory. In this section, we address this gap. To quantify the error incurred in the approximation $\tilde{\rho} \approx \rho$, we employ the trace distance $\frac{1}{2}\|\tilde{\rho} - \rho\|_1$ as it is the most meaningful notion of distance between quantum states, given its operational meaning [**? ?**]. Specifically:

- In Subsubsection S3 A 1, we derive an upper bound on the trace distance between two arbitrary (possibly mixed) Gaussian state in terms of the norm distance between their first moment and their covariance matrices. More explicitly, by using the notation introduced above, we find an upper bound on $\frac{1}{2}\|\tilde{\rho} - \rho\|_1$ in terms of $\|\tilde{\mathbf{m}} - \mathbf{m}(\rho)\|_2$ and $\|\tilde{V} - V(\rho)\|_1$.

- In Subsubsection S3 A 2, we find an improved upper bound on the trace distance between a pure Gaussian state and a possibly-mixed possibly-non-Gaussian state.
- In Subsubsection S3 A 3, we find a lower bound on the trace distance between two arbitrary (possibly mixed) Gaussian state. One may apply this lower bound, together with ε-net techniques, to obtain a lower bound on the sample complexity on tomography of Gaussian states. However, we do not conduct such an analysis in this work since it leads to a too weak lower bound on the sample complexity.

1. Upper bound in the general (possibly mixed-state) setting

This subsubsection is devoted to the proof of the following theorem, which is one of our main technical result.

Theorem S42. *Let ρ, σ be n-mode Gaussian states with mean photon number bounded by N, that is,*

$$\begin{aligned}\mathrm{Tr}\left[\rho\,\hat{N}_n\right] &\le N\,,\\ \mathrm{Tr}\left[\sigma\,\hat{N}_n\right] &\le N\,.\end{aligned}\tag{S220}$$

Then, the trace distance between ρ and σ is upper bounded by

$$\frac{1}{2}\left\|\rho-\sigma\right\|_1 \le f(N)\left(\|\boldsymbol{m}(\rho)-\boldsymbol{m}(\sigma)\|_2+\sqrt{2}\sqrt{\|V(\rho)-V(\sigma)\|_1}\right),\tag{S221}$$

where $f(x) := \frac{1}{\sqrt{2}}\left(\sqrt{x}+\sqrt{x+1}\right)$. Here, $\boldsymbol{m}(\rho), V(\rho)$ denote the first moment and covariance matrix of ρ, while $\boldsymbol{m}(\sigma), V(\sigma)$ denote the first moment and covariance matrix of σ.

Remark S43. *One might be tempted to believe that a straightforward approach exists for establishing an upper bound akin to the one presented in Theorem S42. Specifically, the Fuchs-van de Graaf inequality [**?**] implies that the trace distance between the Gaussian states ρ and σ can be upper bounded in terms of the fidelity $F(\rho,\sigma)$ as*

$$\frac{1}{2}\|\rho-\sigma\|_1 \le \sqrt{1-F(\rho,\sigma)^2}.\tag{S222}$$

*Additionally, a closed formula for the fidelity between Gaussian states is available [**?**]. However, it is important to note that this closed formula is exceedingly intricate, involving determinants, square roots, and inverses of functions of the covariance matrices $V(\rho), V(\sigma)$. Consequently, attempting to derive such an upper bound through this method appears too hard, and, indeed, our attempts in this direction were unsuccessful.*

Before proving Theorem S42, let us first provide some useful preliminary results.

For any positive semi-definite matrix $K \in \mathbb{R}^{2n,2n}$, the *Gaussian noise channel* $\mathcal{N}_K$ is the convex combination of displacement transformations $(\cdot) \mapsto \hat{D}_u(\cdot)\hat{D}_u^\dagger$, where $u \in \mathbb{R}^{2n}$ is distributed according to a Gaussian probability distribution with vanishing mean value and covariance matrix equal to K [**?** , Chapter 5]. Mathematically, $\mathcal{N}_K$ can be written as

$$\mathcal{N}_K(\Theta) := \int_{\mathbb{R}^{2n}} \mathrm{d}^{2n}u\; P_K(u)\,\hat{D}_u\Theta\hat{D}_u^\dagger\,,\tag{S223}$$

where $P_K(u)$ is a Gaussian probability distribution with vanishing mean and covariance matrix equal to K, and $\hat{D}_u := e^{iu^\intercal\Omega_n\hat{\mathbf{R}}}$ is the displacement operator (see the preliminaries subsection S1 C). Of course, in the case when K is strictly positive, $P_K(u)$ can be written as

$$P_K(u) := \frac{e^{-\frac{1}{2}u^\intercal K^{-1}u}}{(2\pi)^n\sqrt{\det K}}\,.\tag{S224}$$

Instead, when K has some zero eigenvalues, the definition of $P_K(u)$ involves Dirac deltas as follows. Let $K=\sum_{i=1}^{2n}\lambda_i v_i v_i^\intercal$ be a spectral decomposition of K, where $(v_i)_{i=1,\ldots,2n}$ are orthonormal eigenvectors, while $\lambda_1,\ldots,\lambda_r>0$ and $\lambda_{r+1},\ldots,\lambda_{2n}=0$ are its eigenvalues, with r being the rank of K. Then, $P_K(u)$ can be written as

$$P_K(u) := \frac{e^{-\frac{1}{2}u^\intercal\left(\sum_{i=1}^{r}\lambda_i^{-1}v_iv_i^\intercal\right)u}}{(2\pi)^{(2n-r)/2}\sqrt{\lambda_1\ldots\lambda_r}}\prod_{i=r+1}^{2n}\delta(v_i^\intercal u)\,,\tag{S225}$$

where $\delta(\cdot)$ denotes the Dirac delta distribution.

It turns out that $\mathcal{N}_K$ leaves the first moments unchanged, and it acts on the covariance matrix of the input state by adding K [**?** , Chapter 5]. For the sake of completeness, we prove the latter fact in the following lemma.

Lemma S44. ([? , Chapter 5]) *Let $K \in \mathbb{R}^{2n,2n}$ be a positive semi-definite matrix. The Gaussian noise channel $\mathcal{N}_K$, defined in (S223), acts on the first moments and covariance matrices as*

$$\boldsymbol{m}(\mathcal{N}_K(\rho)) = \boldsymbol{m}(\rho) \ , \tag{S226}$$
$$V(\mathcal{N}_K(\rho)) = V(\rho) + K \ , \tag{S227}$$

for all input states $\rho \in \mathcal{D}\big(L^2(\mathbb{R}^n)\big)$.

Proof. It holds that

$$\begin{aligned}
\mathbf{m}(\mathcal{N}_K(\rho)) &= \int_{\mathbb{R}^{2n}} \mathrm{d}^{2n}u \, P_K(u) \, \mathbf{m}\Big(\hat{D}_u \rho \hat{D}_u^\dagger\Big) \\
&= \int_{\mathbb{R}^{2n}} \mathrm{d}^{2n}u \, P_K(u) \, \left(\mathbf{m}(\rho) + u\right) \\
&= \mathbf{m}(\rho) + \int_{\mathbb{R}^{2n}} \mathrm{d}^{2n}u \, P_K(u) u \\
&= \mathbf{m}(\rho) \, ,
\end{aligned} \tag{S228}$$

where in the second step we have used Eq. (S64). Moreover, we have that

$$\begin{aligned}
V(\mathcal{N}_K(\rho)) &= \mathrm{Tr}[\{\hat{\mathbf{R}}, \hat{\mathbf{R}}^\intercal\}\mathcal{N}_K(\rho)] - 2\mathbf{m}(\mathcal{N}_K(\rho))\mathbf{m}(\mathcal{N}_K(\rho))^\intercal \\
&= \int_{\mathbb{R}^{2n}} \mathrm{d}^{2n}u \, P_K(u) \, \mathrm{Tr}\left[\rho\{\hat{\mathbf{R}} + u\mathbb{1}, \hat{\mathbf{R}}^\intercal + u^\intercal \mathbb{1}\}\right] - 2\mathbf{m}(\rho)\mathbf{m}(\rho)^\intercal \\
&= V(\rho) + \int_{\mathbb{R}^{2n}} \mathrm{d}^{2n}u \, P_K(u) \, u\mathbf{m}(\rho)^\intercal + \int_{\mathbb{R}^{2n}} \mathrm{d}^{2n}u \, P_K(u) \, \mathbf{m}(\rho)u^\intercal + \int_{\mathbb{R}^{2n}} \mathrm{d}^{2n}u \, P_K(u) \, uu^\intercal \\
&= V(\rho) + K \, ,
\end{aligned} \tag{S229}$$

where in the second step we have used Eq. (S63) and Eq. (S228), and in the last step we solved a Gaussian integral. ☐

Lemma S45. ([? , Chapter 5]) *For any $K \geq 0$, the Gaussian noise channel $\mathcal{N}_K$ is actually a Gaussian channel. That is, $\mathcal{N}_K(\rho)$ is a Gaussian state for any Gaussian state ρ.*

Let us recall the definition of *diamond norm*, as it will be useful for the following. The diamond norm of a superoperator $\Delta : \mathcal{D}(\mathcal{H}) \mapsto \mathcal{D}(\mathcal{H})$ is defined as

$$\|\Delta\|_\diamond := \sup_{\rho \in \mathcal{D}(\mathcal{H} \otimes \mathcal{H}_C)} \|\Delta \otimes \mathrm{Id}_C(\rho)\|_1 \ , \tag{S230}$$

where the sup is taken also over the choice of the ancilla system $\mathcal{H}_C$. In the infinite-dimensional scenario the topology induced by the diamond norm is often too strong (e.g., see Ref. [? , Proposition 1]) and thus it is customary to define the *energy-constrained diamond norm*. Given $N > 0$, given an n-mode system $\mathcal{H}_S = L^2(\mathbb{R}^n)$, the energy-constrained diamond norm of a superoperator $\Delta : \mathcal{D}(\mathcal{H}_S) \mapsto \mathcal{D}(\mathcal{H}_S)$ is defined as [? ? ?]

$$\|\Delta\|_{\diamond N} := \sup_{\rho \in \mathcal{D}(\mathcal{H}_S \otimes \mathcal{H}_C) : \ \mathrm{Tr}\left[\rho \, \hat{N}_n \otimes \mathbb{1}_C\right] \leq N} \|(\Delta \otimes I_C)\rho\|_1 \ , \tag{S231}$$

where the sup is taken also over the choice of the ancilla system $\mathcal{H}_C$, and $\hat{N}_n$ is the n-mode photon number operator on $\mathcal{H}_S$. The supremum in the definition of diamond norm in (S230), as well as the one in (S231), is achieved by taking the ancilla system $\mathcal{H}_C$ to be equal to the input system $\mathcal{H}$.

Lemma S46. ([? , Eq. (3)]) *Let $u \in \mathbb{R}^{2n}$ and let $\mathcal{D}_u(\cdot) := \hat{D}_u(\cdot)\hat{D}_u^\dagger$ be the displacement channel. Then, for all $N > 0$ the energy-constrained diamond norm of the difference between $\mathcal{D}_u$ and the identity map* Id *can be upper bounded as*

$$\frac{1}{2}\|\mathcal{D}_u - \mathrm{Id}\,\|_{\diamond N} \leq \sin\left(\min\left\{\|u\|_2 f(N), \, \frac{\pi}{2}\right\}\right) \ , \tag{S232}$$

where $f(N) := \frac{1}{\sqrt{2}}\left(\sqrt{N} + \sqrt{N+1}\right)$.

Lemma S47 (Diamond norm bound)**.** *Let $K \in \mathbb{R}^{2n,2n}$ be a positive semi-definite matrix. Then, for all $N \geq 0$ the energy-constrained diamond norm of the difference between the Gaussian noise channel $\mathcal{N}_K$ and the identity map Id can be upper bounded as*

$$\frac{1}{2}\left\|\mathcal{N}_K - \mathrm{Id}\right\|_{\diamond N} \leq f(N)\sqrt{\mathrm{Tr}\, K}\,, \tag{S233}$$

where $f(N) \coloneqq \frac{1}{\sqrt{2}}\left(\sqrt{N}+\sqrt{N+1}\right)$.

Proof. It holds that

$$\begin{aligned}
\frac{1}{2}\left\|\mathcal{N}_K - \mathrm{Id}\right\|_{\diamond N} &\overset{\text{(i)}}{\leq} \int \mathrm{d}^{2n}u\; P_K(u)\, \frac{1}{2}\left\|\mathcal{D}_u - \mathrm{Id}\right\|_{\diamond N} \\
&\overset{\text{(ii)}}{\leq} \int \mathrm{d}^{2n}u\; P_K(u)\, \sin\left(\min\left\{\|u\|_2 f(N),\, \frac{\pi}{2}\right\}\right) \\
&\leq f(N) \int \mathrm{d}^{2n}u\; P_K(u)\, \sqrt{u^\intercal u} \\
&\overset{\text{(iii)}}{\leq} f(N)\left(\int \mathrm{d}^{2n}u\; P_K(u)\, u^\intercal u\right)^{1/2} \\
&= f(N)\left(\mathrm{Tr}\, K\right)^{1/2}.
\end{aligned} \tag{S234}$$

Here, in (i), we have used the triangle inequality of the energy-constrained diamond norm and we have introduced the displacement channel $\mathcal{D}_u(\cdot) \coloneqq \hat{D}_u(\cdot)\hat{D}_u^\dagger$. In (ii), we have exploited Lemma S46. In (iii), we have used the concavity of the square root and Jensen inequality. □

Lemma S48 (Expectation value of energy operator)**.** *Let ρ be an n-mode state. The expectation value of the energy operator $\hat{E}_n \coloneqq \frac{\hat{\mathbf{R}}^\intercal \hat{\mathbf{R}}}{2}$ can be expressed in terms of the covariance matrix and the first moment as*

$$\mathrm{Tr}\left[\rho \hat{E}_n\right] = \frac{\mathrm{Tr} V(\rho)}{4} + \frac{\|\boldsymbol{m}(\rho)\|_2^2}{2}\,. \tag{S235}$$

In particular, in terms of the photon number operator $\hat{N}_n \coloneqq \sum_{i=1}^n a_i^\dagger a_i$, it holds that

$$\mathrm{Tr}\left[\rho\, \hat{N}_n\right] = \frac{\mathrm{Tr}[V(\rho) - \mathbb{1}]}{4} + \frac{\|\boldsymbol{m}(\rho)\|_2^2}{2}\,. \tag{S236}$$

Proof. By definition of covariance matrix, it holds that

$$\begin{aligned}
\mathrm{Tr} V(\rho) &= \sum_{i=1}^{2n} V_{i,i}(\rho) \\
&= 2\sum_{i=1}^{2n}\left[\mathrm{Tr}[\hat{R}_i^2 \rho] - m_i(\rho)^2\right] \\
&= 4\,\mathrm{Tr}[\rho \hat{E}_n] - 2\|\mathbf{m}(\rho)\|_2^2\,.
\end{aligned} \tag{S237}$$

Moreover, (S236) follows by exploiting $\hat{E}_n = \hat{N}_n + \frac{n}{2}\hat{\mathbb{1}}$. □

We are now ready to prove Theorem S42.

Proof of Theorem S42. Let us first prove the upper bound in (S221) under the assumption that the Gaussian states have the same first moments. Without loss of generality, we can clearly assume that their first moments vanish, as the trace distance is invariant under (displacement) unitary transformations. Given two Gaussian states ρ and σ with zero first moment, let V be the covariance matrix of ρ, and let W be the covariance matrix of σ. In addition, set

$$T \coloneqq \frac{1}{2}\left(V + W + |V - W|\right), \tag{S238}$$

where we recall that $|V - W| \coloneqq \sqrt{(V-W)^\dagger (V-W)}$. Since $T - V \geq 0$ and $T - W \geq 0$, we can consider the Gaussian noise channels $\mathcal{N}_{T-V}$ and $\mathcal{N}_{T-W}$, as defined in (S223). Since ρ and σ are Gaussian states and since $\mathcal{N}_{T-V}$ and $\mathcal{N}_{T-W}$ are

Gaussian channels (thanks to Lemma S45), it follows that $\mathcal{N}_{T-V}(\rho)$ and $\mathcal{N}_{T-W}(\sigma)$ are Gaussian states. Moreover, by exploiting Lemma S44, both the covariance matrix of $\mathcal{N}_{T-V}(\rho)$ and that of $\mathcal{N}_{T-W}(\sigma)$ are equal to T. Additionally, both the first moment of $\mathcal{N}_{T-V}(\rho)$ and that of $\mathcal{N}_{T-W}(\sigma)$ are equal to the zero vector. Hence, since the Gaussian states $\mathcal{N}_{T-V}(\rho)$ and $\mathcal{N}_{T-W}(\sigma)$ have identical first moments and covariance, they are actually the same state: $\mathcal{N}_{T-V}(\rho) = \mathcal{N}_{T-W}(\sigma)$. Then, we can write

$$\begin{aligned}
\frac{1}{2}\|\rho-\sigma\|_1 &\le \frac{1}{2}\|\rho - \mathcal{N}_{T-V}(\rho) + \mathcal{N}_{T-W}(\sigma) - \sigma\|_1 \\
&\le \frac{1}{2}\|\rho - \mathcal{N}_{T-V}(\rho)\|_1 + \frac{1}{2}\|\mathcal{N}_{T-W}(\sigma) - \sigma\|_1 \\
&= \frac{1}{2}\|(\mathcal{N}_{T-V} - \mathrm{Id})(\rho)\|_1 + \frac{1}{2}\|(\mathcal{N}_{T-W} - \mathrm{Id})(\sigma)\|_1 \\
&\le \frac{1}{2}\|\mathcal{N}_{T-V} - \mathrm{Id}\|_{\diamond N} + \frac{1}{2}\|\mathcal{N}_{T-W} - \mathrm{Id}\|_{\diamond N} \\
&\overset{\text{(i)}}{\le} f(N)\left(\sqrt{\mathrm{Tr}[T-V]} + \sqrt{\mathrm{Tr}[T-W]}\right) \\
&\overset{\text{(ii)}}{\le} 2\,f(N)\sqrt{\frac{1}{2}\mathrm{Tr}[T-V] + \frac{1}{2}\mathrm{Tr}[T-W]} \\
&= \sqrt{2}\,f(N)\sqrt{\mathrm{Tr}\,|V-W|} \\
&= \sqrt{2}\,f(N)\,\sqrt{\|V-W\|_1}\,,
\end{aligned} \tag{S239}$$

where in (i) we have used Lemma S47, and in (ii) we have used the concavity of the square root. This proves the claim under the assumption that the Gaussian states ρ and σ have zero first moment.

For the general case, we can denote by $\delta \coloneqq \mathbf{m}(\rho) - \mathbf{m}(\sigma)$ the difference between the first moments of ρ and σ. Then

$$\begin{aligned}
\frac{1}{2}\|\rho-\sigma\|_1 &\overset{\text{(iii)}}{=} \frac{1}{2}\left\|\hat{D}_{-\mathbf{m}(\sigma)}\rho\hat{D}^\dagger_{-\mathbf{m}(\sigma)} - \hat{D}_{-\mathbf{m}(\sigma)}\sigma\hat{D}^\dagger_{-\mathbf{m}(\sigma)}\right\|_1 \\
&= \frac{1}{2}\left\|\hat{D}_\delta\,\hat{D}_{-\mathbf{m}(\rho)}\rho\hat{D}^\dagger_{-\mathbf{m}(\rho)}\,\hat{D}^\dagger_\delta - \hat{D}_{-\mathbf{m}(\sigma)}\sigma\hat{D}^\dagger_{-\mathbf{m}(\sigma)}\right\|_1 \\
&\le \frac{1}{2}\left\|\hat{D}_\delta\,\hat{D}_{-\mathbf{m}(\rho)}\rho\hat{D}^\dagger_{-\mathbf{m}(\rho)}\,\hat{D}^\dagger_\delta - \hat{D}_{-\mathbf{m}(\rho)}\rho\hat{D}^\dagger_{-\mathbf{m}(\rho)}\right\|_1 + \frac{1}{2}\left\|\hat{D}_{-\mathbf{m}(\rho)}\rho\hat{D}^\dagger_{-\mathbf{m}(\rho)} - \hat{D}_{-\mathbf{m}(\sigma)}\sigma\hat{D}^\dagger_{-\mathbf{m}(\sigma)}\right\|_1 \\
&\overset{\text{(iv)}}{\le} \frac{1}{2}\|\mathcal{D}_\delta - \mathrm{Id}\|_{\diamond N} + \frac{1}{2}\left\|\hat{D}_{-\mathbf{m}(\rho)}\rho\hat{D}^\dagger_{-\mathbf{m}(\rho)} - \hat{D}_{-\mathbf{m}(\sigma)}\sigma\hat{D}^\dagger_{-\mathbf{m}(\sigma)}\right\|_1 \\
&\overset{\text{(v)}}{\le} \frac{1}{2}\|\mathcal{D}_\delta - \mathrm{Id}\|_{\diamond N} + \sqrt{2}\,f(N)\,\sqrt{\|V(\rho)-V(\sigma)\|_1} \\
&\overset{\text{(vi)}}{\le} f(N)\left(\|\delta\|_2 + \sqrt{2}\,\sqrt{\|V(\rho)-V(\sigma)\|_1}\right)\,.
\end{aligned} \tag{S240}$$

Here, in (iii), we have used the unitary invariance of the trace norm. In (iv), we have exploited the definition of energy-constrained diamond norm in Eq. (S231), together with the fact that

$$\mathrm{Tr}\left[\hat{N}_n\hat{D}_{-\mathbf{m}(\rho)}\rho\hat{D}^\dagger_{-\mathbf{m}(\rho)}\right] = \frac{\mathrm{Tr}[V(\rho)-\mathbb{1}]}{4} \le \frac{\mathrm{Tr}[V(\rho)-\mathbb{1}]}{4} + \frac{\|\mathbf{m}(\rho)\|_2^2}{2} = \mathrm{Tr}\left[\hat{N}_n\rho\right] \le N\,, \tag{S241}$$

where we have exploited Lemma S48. In (v), we leveraged (S239), as the two Gaussian states $\hat{D}_{-\mathbf{m}(\rho)}\rho\hat{D}^\dagger_{-\mathbf{m}(\rho)}$ and $\hat{D}_{-\mathbf{m}(\sigma)}\sigma\hat{D}^\dagger_{-\mathbf{m}(\sigma)}$ have zero first moment. Finally, in (vi) we have exploited Lemma S46, together with the inequality $\sin(x) \le x$ for all $x \ge 0$. □

2. *Upper bound in the pure-state setting*

Theorem S42 provides an upper bound on the trace distance between two Gaussian states ρ and σ, which are possibly mixed. We can obtain a tighter bound if we assume that one of the two states is pure, as proved in the following theorem.

Theorem S49. *Let $\psi \coloneqq |\psi\rangle\langle\psi|$ be a pure n-mode Gaussian state with first moment $\boldsymbol{m}(\psi)$ and second moment $V(\psi)$. Let ρ be an n-mode possibly non-Gaussian state with first moment $\boldsymbol{m}(\rho)$ and second moment $V(\rho)$. Then*

$$\|\,\rho - |\psi\rangle\langle\psi|\,\|_1 \le \sqrt{\mathrm{Tr}\,V(\psi)}\sqrt{\|V(\rho)-V(\psi)\|_\infty + 2\|\boldsymbol{m}(\rho)-\boldsymbol{m}(\psi)\|_2^2} \tag{S242}$$

In particular, if the mean energy of ψ is upper bounded by a constant E, that is,

$$\mathrm{Tr}\left[\rho\hat{E}_n\right]\le E \tag{S243}$$

where $\hat{E}_n:=\frac{\hat{\mathbf{R}}^\intercal\hat{\mathbf{R}}}{2}$*, then*

$$\frac{1}{2}\left\|\,\rho-|\psi\rangle\!\langle\psi|\,\right\|_1\le\sqrt{E}\sqrt{\|V(\rho)-V(\psi)\|_\infty+2\|\boldsymbol{m}(\rho)-\boldsymbol{m}(\psi)\|_2^2} \tag{S244}$$

Before proving Theorem S49, let us show some useful lemmas.

Lemma S50. *Let ψ be an n-mode pure Gaussian state. The inverse of the covariance matrix reads*

$$V(\psi)^{-1}=\Omega_nV(\psi)\Omega_n^\intercal\,, \tag{S245}$$

where Ω_n is the symplectic form defined in (S35).

Proof. The covariance matrix $V(\psi)$ of the pure Gaussian state ψ can be written as $V(\psi)=SS^\intercal$ with $S\in\mathrm{Sp}(2n)$ being a symplectic matrix (due to (S67)). Consequently, it holds that

$$\Omega_n^\intercal V(\psi)^{-1}\Omega_n=-\Omega_nV(\psi)^{-1}\Omega_n=-\Omega_n(S^\intercal)^{-1}S^{-1}\Omega_n=-S\Omega_n^2S^\intercal=SS^\intercal=V(\psi)\,, \tag{S246}$$

where we have used that $S^{-1}\in\mathrm{Sp}(2n)$. □

Lemma S51. *For any symplectic $S\in\mathrm{Sp}(2n)$ and for any unitarily invariant norm $\|\cdot\|$, it holds that*

$$\|S\|=\|S^{-1}\|\,. \tag{S247}$$

Proof. By exploiting that $S^{-1}\in\mathrm{Sp}(2n)$, we have that

$$S^{-1}=-S^{-1}\Omega_n\Omega_n=-\Omega_nS^\intercal\Omega_n=\Omega_nS^\intercal\Omega_n^\intercal\,. \tag{S248}$$

In particular, since Ω_n is orthogonal, it holds that $\|S^{-1}\|=\|S^\intercal\|=\|S\|$. □

We are now ready to prove Theorem S49.

Proof of Theorem S49. Since ψ is a pure Gaussian state, the symplectic eigenvalues of its covariance matrix $V(\psi)$ are all equal to 1. Consequently, there exists a symplectic matrix S such that

$$\begin{aligned}V(\psi)&=SS^\intercal\,,\\|\psi\rangle&=\hat{D}_{\mathbf{m}(\psi)}U_S\,|0\rangle\ ,\end{aligned} \tag{S249}$$

where we have denoted as $|0\rangle$ the n-mode vacuum state vector. Hence, it holds that

$$\langle\psi|\,\rho\,|\psi\rangle=\langle0|\,U_S^\dagger\hat{D}^\dagger_{\mathbf{m}(\psi)}\rho\hat{D}_{\mathbf{m}(\psi)}U_S\,|0\rangle=\langle0|\,\omega\,|0\rangle\ , \tag{S250}$$

where we have introduced the state

$$\omega:=U_S^\dagger\hat{D}^\dagger_{\mathbf{m}(\psi)}\rho\hat{D}_{\mathbf{m}(\psi)}U_S\,. \tag{S251}$$

By exploiting the operator inequality

$$|0\rangle\!\langle0|\ge\mathbb{1}-\sum_{i=1}^na_i^\dagger a_i\,, \tag{S252}$$

we find that

$$\begin{aligned}\langle\psi|\,\rho\,|\psi\rangle&=\langle0|\,\omega\,|0\rangle\\&=\mathrm{Tr}[\omega\,|0\rangle\!\langle0|]\\&\ge1-\mathrm{Tr}\left[\omega\sum_{i=1}^na_i^\dagger a_i\right]\\&=1-\frac{\mathrm{Tr}[V(\omega)-\mathbb{1}]}{4}-\frac{\|\mathbf{m}(\omega)\|_2^2}{2}\,,\end{aligned} \tag{S253}$$

where we also used Lemma S48. Note that the first moment and covariance matrix of ω are given by

$$\begin{aligned}\mathbf{m}(\omega) &= S^{-1}\left(\mathbf{m}(\rho)-\mathbf{m}(\psi)\right)\,,\\ V(\omega) &= S^{-1}V(\rho)(S^{-1})^\intercal\,.\end{aligned} \tag{S254}$$

Consequently, it holds that

$$\begin{aligned}|\mathrm{Tr}[V(\omega)-\mathbb{1}]| &= \left|\mathrm{Tr}[S^{-1}V(\rho)(S^{-1})^\intercal-\mathbb{1}]\right|\\ &= \left|\mathrm{Tr}[S^{-1}(V(\rho)-V(\psi))(S^{-1})^\intercal]\right|\\ &\overset{\text{(i)}}{\le} \|(S^{-1})^\intercal S^{-1}\|_1\,\|V(\rho)-V(\psi)\|_\infty\\ &\overset{\text{(ii)}}{=} \mathrm{Tr}\left[(S^{-1})^\intercal S^{-1}\right]\|V(\rho)-V(\psi)\|_\infty\\ &= \mathrm{Tr}[V(\psi)^{-1}]\,\|V(\rho)-V(\psi)\|_\infty\\ &\overset{\text{(iii)}}{=} \mathrm{Tr}\,V(\psi)\,\|V(\rho)-V(\psi)\|_\infty\,.\end{aligned} \tag{S255}$$

Here, in (i), we have used Hölder inequality $|\,\mathrm{Tr}[AB]|\le\|A\|_1\|B\|_\infty$. In (ii), we have exploited that $(S^{-1})^\intercal S^{-1}$ is positive semi-definite. Moreover, in (iii) we utilised Lemma S50, which asserts that the inverse of the covariance matrix $V(\psi)$ of the pure Gaussian state ψ can be written as $V(\psi)^{-1}=-\Omega_nV(\psi)\Omega_n$, and, in particular, this implies $\mathrm{Tr}\,V(\psi)=\mathrm{Tr}[V(\psi)^{-1}]$. In addition, it holds that

$$\begin{aligned}\|\mathbf{m}(\omega)\|_2 &= \|S^{-1}\left(\mathbf{m}(\rho)-\mathbf{m}(\psi)\right)\|_2\\ &\le \|S^{-1}\|_\infty\,\|\mathbf{m}(\rho)-\mathbf{m}(\psi)\|_2\\ &\overset{\text{(iv)}}{=} \|S\|_\infty\,\|\mathbf{m}(\rho)-\mathbf{m}(\psi)\|_2\\ &\le \sqrt{\mathrm{Tr}[SS^\intercal]}\,\|\mathbf{m}(\rho)-\mathbf{m}(\psi)\|_2\\ &= \sqrt{\mathrm{Tr}\,V(\psi)}\,\|\mathbf{m}(\rho)-\mathbf{m}(\psi)\|_2\,,\end{aligned} \tag{S256}$$

where in (iv) we have exploited Lemma S51. Consequently, (S253) implies that

$$\langle\psi|\,\rho\,|\psi\rangle \ge 1-\frac{\mathrm{Tr}\,V(\psi)}{4}\left(\|V(\rho)-V(\psi)\|_\infty+2\|\mathbf{m}(\rho)-\mathbf{m}(\psi)\|_2^2\right) \tag{S257}$$

Hence, Fuchs-van de Graaf inequality implies that

$$\begin{aligned}\|\rho-\psi\|_1 &\le 2\sqrt{1-\langle\psi|\,\rho\,|\psi\rangle}\\ &\le \sqrt{\mathrm{Tr}\,V(\psi)}\sqrt{\|V(\rho)-V(\psi)\|_\infty+2\|\mathbf{m}(\rho)-\mathbf{m}(\psi)\|_2^2}\,.\end{aligned} \tag{S258}$$

In particular, by exploiting Lemma S48 and the assumption that the mean energy of ψ is upper bounded by E, we conclude that

$$\mathrm{Tr}\,V(\psi)=4\,\mathrm{Tr}[\hat{E}_n\psi]-2\|\mathbf{m}(\psi)\|_2^2\le 4E\,. \tag{S259}$$

□

3. *Lower bound*

In this subsection we obtain a lower bound on the trace distance between Gaussian states in terms of the norm distance between their covariance matrices and their first moments.

Theorem S52. *Let ρ_1 and ρ_2 be n-mode Gaussian states with mean energy upper bounded by E, i.e.,*

$$\begin{aligned}\mathrm{Tr}\left[\rho_1\hat{E}_n\right] &\le E\,,\\ \mathrm{Tr}\left[\rho_2\hat{E}_n\right] &\le E\,.\end{aligned} \tag{S260}$$

Then, the trace distance between ρ_1 and ρ_2 can be lower bounded in terms of the norm distance between their first moments as

$$\frac{1}{2}\|\rho_1-\rho_2\|_1\ge\frac{1}{200}\min\left\{1,\frac{\|\boldsymbol{m}(\rho_1)-\boldsymbol{m}(\rho_2)\|_2}{\sqrt{4E+1}}\right\}\,, \tag{S261}$$

and in terms of the norm distance between their covariance matrices as

$$\frac{1}{2}\|\rho_1-\rho_2\|_1 \geq \frac{1}{200}\min\left\{1, \frac{\|V(\rho_2)-V(\rho_1)\|_2}{4E+1}\right\}. \tag{S262}$$

Proof. Recall that the Husimi function $Q_\rho : \mathbb{R}^{2n} \longmapsto \mathbf{R}$ of an n-mode quantum state ρ is defined as [**?**]

$$Q_\rho(\mathbf{r}) := \frac{1}{(2\pi)^n}\langle\mathbf{r}|\,\rho\,|\mathbf{r}\rangle\,, \tag{S263}$$

where $|\mathbf{r}\rangle := \hat{D}_\mathbf{r}|0\rangle$ is a coherent state. The Husimi function Q_ρ is the probability distribution of the outcome $\mathbf{r}$ of the heterodyne measurement performed on ρ [**?**]. Hence, by exploiting the monotonicity of the trace norm under quantum channels, the trace distance between ρ_1 and ρ_2 can be lower bounded in terms of the TV distance between their Husimi functions as

$$\frac{1}{2}\|\rho_1-\rho_2\|_1 \geq \frac{1}{2}\int_{\mathbf{r}\in\mathbb{R}^{2n}} \mathrm{d}^{2n}\mathbf{r}\,|Q_{\rho_1}(\mathbf{r})-Q_{\rho_2}(\mathbf{r})| = d_{\mathrm{TV}}\left(Q_{\rho_1},Q_{\rho_2}\right)\,, \tag{S264}$$

where in the last equality we have used the definition of TV distance. The Husimi function of a Gaussian state ρ is a Gaussian probability distribution with first moment $\mathbf{m}(\rho)$ and covariance matrix $\frac{V(\rho)+\mathbb{1}}{2}$ (see (S54) in the preliminaries section):

$$Q_\rho(\mathbf{r}) = \mathcal{N}\left[\mathbf{m}(\rho), \frac{V(\rho)+\mathbb{1}}{2}\right](\mathbf{r})\,, \tag{S265}$$

where $\mathcal{N}$ denotes a Gaussian probability distribution defined as follows. Given a real vector $\mathbf{m}$ and a positive matrix V, the Gaussian probability distribution $\mathcal{N}[\mathbf{m},V]$ with first moment $\mathbf{m}$ and covariance matrix V is defined as

$$\mathcal{N}[\mathbf{m},V](\mathbf{r}) := \frac{e^{-\frac{1}{2}(\mathbf{r}-\mathbf{m})^\intercal V^{-1}(\mathbf{r}-\mathbf{m})}}{(2\pi)^n\sqrt{\det V}}\,. \tag{S266}$$

Consequently, (S264) implies that

$$\frac{1}{2}\|\rho_1-\rho_2\|_1 \geq d_{\mathrm{TV}}\left(\mathcal{N}\left[\mathbf{m}(\rho_1), \frac{V(\rho_1)+\mathbb{1}}{2}\right], \mathcal{N}\left[\mathbf{m}(\rho_2), \frac{V(\rho_2)+\mathbb{1}}{2}\right]\right). \tag{S267}$$

Now, let us leverage recent results about tight bounds on the TV distance between two arbitrary Gaussian probability distributions [**?**]. By exploiting [**?** , Theorem 1.1], together with [**?** , Lemma B.3], the TV distance between two Gaussian probability distributions $\mathcal{N}[\mathbf{m}_1,V_1]$ and $\mathcal{N}[\mathbf{m}_2,V_2]$ can be lower bounded in terms of the norm distance between covariance matrices as

$$d_{\mathrm{TV}}\left(\mathcal{N}[\mathbf{m}_1,V_1],\mathcal{N}[\mathbf{m}_2,V_2]\right) \geq \frac{\min\left\{1, \left\|V_1^{-1/2}V_2V_1^{-1/2}-\mathbb{1}\right\|_2\right\}}{200}\,. \tag{S268}$$

Note that we can lower bound the term $\left\|V_1^{-1/2}V_2V_1^{-1/2}-\mathbb{1}\right\|_2$ in terms of $\|V_2-V_1\|_2$ as

$$\begin{aligned}\left\|V_1^{-1/2}V_2V_1^{-1/2}-\mathbb{1}\right\|_2 &= \left\|V_1^{-1/2}\left(V_2-V_1\right)V_1^{-1/2}\right\|_2\\ &= \sqrt{\mathrm{Tr}\left[V_1^{-1}(V_2-V_1)V_1^{-1}(V_2-V_1)\right]}\\ &\geq \sqrt{\mathrm{Tr}\left[V_1^{-1}(V_2-V_1)\frac{\mathbb{1}}{\|V_1\|_\infty}(V_2-V_1)\right]}\\ &\geq \frac{\sqrt{\mathrm{Tr}[(V_2-V_1)^2]}}{\|V_1\|_\infty}\\ &= \frac{\|V_2-V_1\|_2}{\|V_1\|_\infty}\,.\end{aligned} \tag{S269}$$

Consequently, we obtain the following lower bound on the TV distance between two Gaussian probability distributions

$$d_{\mathrm{TV}}\left(\mathcal{N}[\mathbf{m}_1,V_1],\mathcal{N}[\mathbf{m}_2,V_2]\right) \geq \frac{1}{200}\min\left\{1, \frac{\|V_2-V_1\|_2}{\|V_1\|_\infty}\right\}. \tag{S270}$$

In addition, thanks to Ref. [? , Theorem 1.1], the TV distance between two Gaussian probability distributions can also be lower bounded as

$$d_{\mathrm{TV}}\left(\mathscr{N}[\mathbf{m}_1, V_1], \mathscr{N}[\mathbf{m}_2, V_2]\right) \geq \frac{1}{200}\min\left\{1, \frac{\|\mathbf{m}_1-\mathbf{m}_2\|_2^2}{\sqrt{(\mathbf{m}_1-\mathbf{m}_2)^{\intercal}V_1\left(\mathbf{m}_1-\mathbf{m}_2\right)}}\right\}, \tag{S271}$$

which implies that

$$d_{\mathrm{TV}}\left(\mathscr{N}[\mathbf{m}_1, V_1], \mathscr{N}[\mathbf{m}_2, V_2]\right) \geq \frac{1}{200}\min\left\{1, \frac{\|\mathbf{m}_1-\mathbf{m}_2\|_2}{\sqrt{\|V_1\|_\infty}}\right\}. \tag{S272}$$

Consequently, by using (S267), together with (S270) and (S272), we obtain that

$$\begin{aligned}\frac{1}{2}\|\rho_1-\rho_2\|_1 &\geq \frac{1}{200}\min\left\{1, \frac{\|\mathbf{m}(\rho_1)-\mathbf{m}(\rho_2)\|_2}{\sqrt{\|V(\rho_1)+\mathbb{1}\|_\infty}}\right\}, \\ \frac{1}{2}\|\rho_1-\rho_2\|_1 &\geq \frac{1}{200}\min\left\{1, \frac{\|V(\rho_2)-V(\rho_1)\|_2}{\|V(\rho_1)+\mathbb{1}\|_\infty}\right\}.\end{aligned} \tag{S273}$$

Hence, since any n-mode state ρ satisfies $\|V(\rho)\|_\infty \leq 4\,\mathrm{Tr}[\rho\hat{E}_n]$, the assumption on the energy constraints in (S260) implies that

$$\begin{aligned}\frac{1}{2}\|\rho_1-\rho_2\|_1 &\geq \frac{1}{200}\min\left\{1, \frac{\|\mathbf{m}(\rho_1)-\mathbf{m}(\rho_2)\|_2}{\sqrt{4E+1}}\right\}, \\ \frac{1}{2}\|\rho_1-\rho_2\|_1 &\geq \frac{1}{200}\min\left\{1, \frac{\|V(\rho_2)-V(\rho_1)\|_2}{4E+1}\right\}.\end{aligned} \tag{S274}$$

□

B. Learning first moments and covariance matrices

In this subsection, we conduct the sample complexity analysis of an algorithm for estimating the first moment and covariance matrix of a (possibly non-Gaussian) quantum state. Recall that the covariance matrix $V(\rho)$ is defined by

$$V(\rho) = \mathrm{Tr}[\{\hat{\mathbf{R}}, \hat{\mathbf{R}}^{\intercal}\}\rho] - 2\mathbf{m}(\rho)\mathbf{m}(\rho)^{\intercal}, \tag{S275}$$

where $\mathbf{m}(\rho) := \mathrm{Tr}[\rho\hat{\mathbf{R}}]$ represents the first moment vector, and $\hat{\mathbf{R}} = (\hat{x}_1, \hat{p}_1, \hat{x}_2, \hat{p}_2, \ldots, \hat{x}_n, \hat{p}_n)$ denotes the quadrature vector. Additionally, the covariance matrix of any state must satisfy the uncertainty relation:

$$V(\rho) + i\Omega_n \geq 0. \tag{S276}$$

A direct method for estimating the covariance matrix involves estimating the first moment and the expectation values of all observables $(\{\hat{R}_i, \hat{R}_j\})_{i,j\in[2n]}$. However, there is a more efficient algorithm, as detailed by Aolita et al. [?], which requires fewer copies of ρ. We adopt this approach and provide a sample complexity analysis that extends beyond what was outlined in Ref. [?].

The algorithm's strategy involves grouping commuting observables whose expectation values form entries of the covariance matrix and performing joint measurements on these groups of commuting observables. This estimation procedure yields a symmetric matrix $\tilde{V} \in \mathbb{R}^{2n\times 2n}$ as an approximation of $V(\rho)$. However, the estimated matrix $\tilde{V}$ may not satisfy the uncertainty relation $\tilde{V} + i\Omega_n \geq 0$, implying that there might not exist a quantum state $\tilde{\rho}$ with covariance matrix $\tilde{V}$. We address this issue by showing that slightly perturbing $\tilde{V}$ suffices to obtain a valid covariance matrix. Ensuring that the estimated matrix is a valid covariance matrix is crucial, for example, when we need to consider a Gaussian state with covariance matrix $\tilde{V}$ (as we will do in Subsection S3 C), or when applying the Williamson decomposition to $\tilde{V}$ (e.g., we will need this in Section S4).

The algorithm's detailed steps are provided in Table S2. Notably, this algorithm exclusively relies on homodyne measurements, which are experimentally feasible in photonic platforms. In the forthcoming Theorem S53, we establish the algorithm's correctness and demonstrate that its sample complexity is $O\left(\log\left(\frac{n^2}{\delta}\right)\frac{n^3E^2}{\varepsilon^2}\right)$.

Table S2. Estimation algorithm for the first moment $\mathbf{m}(\rho)$ and covariance matrix $V(\rho)$ of an arbitrary n-mode quantum state ρ satisfying a second moment constraint.

Input: Accuracy ε, failure probability δ, second moment constraint E, N copies of the unknown n-mode quantum state ρ satisfying the moment constraint $\sqrt{\mathrm{Tr}\left[\hat{E}_n^2\rho\right]} \le nE$, where

$$N := \ (n+3)\left\lceil 68\log\left(\frac{2(2n^2+3n)}{\delta}\right)\frac{200(8n^2E^2+3n)}{\varepsilon^2}\right\rceil = O\left(\log\left(\frac{n^2}{\delta}\right)\frac{n^3E^2}{\varepsilon^2}\right)\,. \tag{S277}$$

Output: A vector $\tilde{\mathbf{m}} \in \mathbb{R}^{2n}$ and a symmetric matrix $\tilde{V}' \in \mathbb{R}^{2n,2n}$ such that

$$\Pr\left(\|\tilde{V}' - V(\rho)\|_\infty \le \varepsilon \quad \text{and} \quad \|\tilde{\mathbf{m}} - \mathbf{m}(\rho)\|_2 \le \frac{\varepsilon}{10\sqrt{8En}}\right) \ge 1-\delta\,. \tag{S278}$$

Set $N' := N/(n+3)$.
Query N' copies of ρ and, for each, perform a joint measurement of the position observables $\hat{x}_1, \hat{x}_2, \ldots, \hat{x}_n$. Then, construct median-of-means estimators (Lemma S4) of the expectation values of $(\{\hat{x}_i,\hat{x}_j\})_{i\le j}$ and of $(\hat{x}_i)_{i\in[n]}$.
Query N' copies of ρ and, for each, perform a joint measurement of the momentum observables $\hat{p}_1, \hat{p}_2, \ldots, \hat{p}_n$. Then, construct median-of-means estimators of the expectation values of $(\{\hat{p}_i,\hat{p}_j\})_{i\le j}$ and of $(\hat{p}_i)_{i\in[n]}$.
Query N' copies of ρ and, for each, perform a joint measurement of $\{\hat{x}_1,\hat{p}_1\}, \{\hat{x}_2,\hat{p}_2\}, \ldots, \{\hat{x}_n,\hat{p}_n\}$. Then, construct median-of-means estimators of the expectation value of $(\{\hat{x}_i,\hat{p}_i\})_{i\in[n]}$.
for $k \leftarrow 1$ **to** n **do**
    Query N' copies of ρ and, for each, perform a joint measurement of $(\hat{x}_i)_{i\neq k}$ and $\hat{p}_k$. Then, construct median-of-means estimators of the expectation values of $(\{\hat{p}_k,\hat{x}_i\})_{i\neq k}$.
end for
Combine all the aforementioned estimates to form the estimator $\tilde{\mathbf{m}} \in \mathbb{R}^{2n}$ for the first moment $\mathbf{m}(\rho)$, and the estimator $\tilde{W} \in \mathbb{R}^{2n,2n}$ for the matrix $\mathrm{Tr}[\rho\{\hat{\mathbf{R}},\hat{\mathbf{R}}^\intercal\}]$.
Set $\tilde{V} := \tilde{W} - 2\tilde{\mathbf{m}}\tilde{\mathbf{m}}^\intercal$.
Set $\tilde{V}' := \tilde{V} + \frac{\varepsilon}{2}\mathbb{1}$.
if $\tilde{V}' + i\Omega_n$ is positive semi-definite **then**
    return the estimator $\tilde{\mathbf{m}}$ for the first moment and the estimator $\tilde{V}'$ for the covariance matrix.
else
    Declare *failure* and abort.
end if

Theorem S53. *Let $\varepsilon,\delta \in (0,1)$ and $E \ge 0$. Let ρ be an n-mode quantum state satisfying the second moment constraint $\sqrt{\mathrm{Tr}\left[\hat{E}_n^2\rho\right]} \le nE$. Then, N copies of ρ, satisfying*

$$\begin{aligned} N &:= (n+3)\left\lceil 68\log\left(\frac{2(2n^2+3n)}{\delta}\right)\frac{200(8n^2E^2+3n)}{\varepsilon^2}\right\rceil \\ &= O\left(\log\left(\frac{n^2}{\delta}\right)\frac{n^3E^2}{\varepsilon^2}\right)\,, \end{aligned} \tag{S279}$$

are sufficient to build a vector $\tilde{\mathbf{m}} \in \mathbb{R}^{2n}$ and a symmetric matrix $\tilde{V}' \in \mathbb{R}^{2n,2n}$ such that

$$\Pr\left(\|\tilde{V}' - V(\rho)\|_\infty \le \varepsilon \quad \text{and} \quad \tilde{V}' + i\Omega_n \ge 0 \quad \text{and} \quad \|\tilde{\mathbf{m}} - \mathbf{m}(\rho)\|_2 \le \frac{\varepsilon}{10\sqrt{8En}}\right) \ge 1-\delta\,. \tag{S280}$$

Before proving Theorem S53, let us show the following lemma.

Lemma S54. *For all $n \in \mathbb{N}$, it holds that*

$$\sum_{j,k=1}^{2n}\{\hat{R}_j,\hat{R}_k\}^2 = 16\hat{E}_n^2 + 6n\hat{\mathbb{1}}\,, \tag{S281}$$

where $\hat{E}_n := \frac{\hat{\mathbf{R}}^\intercal\hat{\mathbf{R}}}{2}$ is the energy operator.

Proof. Note that

$$\begin{aligned}\sum_{j,k=1}^{2n}\{\hat R_j,\hat R_k\}^2 &= \sum_{j,k=1}^{2n}\left(\hat R_j\hat R_k+\hat R_k\hat R_j\right)^2\\ &=2\sum_{j,k=1}^{2n}\left(\hat R_j\hat R_k\hat R_j\hat R_k+\hat R_j\hat R_k^2\hat R_j\right)\\ &\overset{\text{(i)}}{=}2\sum_{j,k=1}^{2n}\left(2\hat R_j^2\hat R_k^2-3i\Omega_{jk}\hat R_j\hat R_k\right)\\ &\overset{\text{(ii)}}{=}4\sum_{j,k=1}^{2n}\hat R_j^2\hat R_k^2+6n\hat{\mathbb{1}}\\ &=4\left(\hat{\mathbf R}^\intercal\hat{\mathbf R}\right)^2+6n\hat{\mathbb{1}}\\ &=16\hat E_n^2+6n\hat{\mathbb{1}}\,.\end{aligned}\tag{S282}$$

Here, in (i), we have used three times that $\hat R_k\hat R_j=\hat R_j\hat R_k-i\Omega_{jk}\hat{\mathbb{1}}$ and, in (ii), we have used that $\sum_{j,k=1}^{2n}\Omega_{jk}\hat R_j\hat R_k=in\hat{\mathbb{1}}$. □

Proof of Theorem S53. We aim to establish the correctness of the algorithm presented in Table S2. In Line 2, we estimate the expectation value of $\{\hat x_i,\hat x_j\}$ for each $i\le j\in[n]$ and the expectation value of $\hat x_i$ for each $i\in[n]$, amounting to $\frac{n(n+1)}{2}+n$ quantities. In Line 3, we estimate the expectation value of $\{\hat p_i,\hat p_j\}$ for each $i\le j\in[n]$ and the expectation value of $\hat p_i$ for each $i\in[n]$, which are also $\frac{n(n+1)}{2}+n$ quantities. In Line 4, we estimate the expectation value of $\{\hat x_i,\hat p_i\}$ for each $i\in[n]$, constituting n quantities. Finally, in all iterations of Line 6, we estimate the expectation value of $\{\hat p_k,\hat x_i\}$ for each $i\neq k\in[n]$, resulting in $n(n-1)$ quantities. Consequently, across Lines 2–6, we estimate a total of $2n^2+3n$ quantities. By combining all these estimates, we can construct an estimator $\tilde{\mathbf m}\in\mathbb R^{2n}$ for the first moment $\mathbf m(\rho)$, and an estimator $\tilde W\in\mathbb R^{2n,2n}$ for the matrix $\mathrm{Tr}[\rho\{\hat{\mathbf R},\hat{\mathbf R}^\intercal\}]$. Therefore, by making use of Lemma S4 together with an union bound, for all $\varepsilon'\in(0,1)$ we can choose

$$N'\ge 68\log\left(\frac{2(2n^2+3n)}{\delta}\right)\frac{16E^2+6/n}{\varepsilon'^2}\tag{S283}$$

to ensure that with probability at least $1-\delta$ it holds that

$$\left|\tilde W_{i,j}-\mathrm{Tr}[\rho\{\hat R_i,\hat R_j\}]\right|\le\varepsilon'\sqrt{\frac{\mathrm{Tr}[\rho\{\hat R_i,\hat R_j\}^2]}{16E^2+6/n}}\qquad\forall\, i,j\in[2n]\,,\tag{S284}$$

and

$$|\tilde m_i-m_i(\rho)|\le\varepsilon'\sqrt{\frac{\mathrm{Tr}[\rho\hat R_i^2]}{16E^2+6/n}}\qquad\forall\, i\in[2n]\,.\tag{S285}$$

Here, we have used the fact that the variance of the random variable considered to construct the estimator $\tilde W_{i,j}$ can be upper bounded by $\mathrm{Tr}[\rho\{\hat R_i,\hat R_j\}^2]$ for each $i,j\in[2n]$, and the variance of the random variable considered to construct the estimator $\tilde m_i$ can be upper bounded by $\mathrm{Tr}[\rho\hat R_i^2]$ for each $i\in[2n]$. Moreover, if (S284) holds, then we have that

$$\begin{aligned}\left\|\tilde W-\mathrm{Tr}[\rho\{\hat{\mathbf R},\hat{\mathbf R}^\intercal\}]\right\|_\infty &\le\left\|\tilde W-\mathrm{Tr}[\rho\{\hat{\mathbf R},\hat{\mathbf R}^\intercal\}]\right\|_2\\ &=\sqrt{\sum_{i,j=1}^{2n}\left|\tilde W_{i,j}-\mathrm{Tr}[\rho\{\hat R_i,\hat R_j\}]\right|^2}\\ &\le\varepsilon'\sqrt{\frac{\sum_{i,j=1}^{2n}\mathrm{Tr}[\rho\{\hat R_i,\hat R_j\}^2]}{16E^2+6/n}}\\ &=n\varepsilon'\,,\end{aligned}\tag{S286}$$

where the last equality is a consequence of Lemma S54. In addition, if (S285) holds, we have that

$$\begin{aligned}
\|\tilde{\mathbf{m}}-\mathbf{m}(\rho)\|_2 &\le \varepsilon'\sqrt{\frac{\sum_{i=1}^{2n}\mathrm{Tr}[\rho\hat{R}_i^2]}{16E^2+6/n}} \\
&= \varepsilon'\sqrt{\frac{2\,\mathrm{Tr}[\rho\hat{E}_n]}{16E^2+6/n}} \\
&\overset{\text{(i)}}{\le} \varepsilon'\sqrt{\frac{2\sqrt{\mathrm{Tr}[\rho\hat{E}_n^2]}}{16E^2+6/n}} \\
&\le \varepsilon'\sqrt{\frac{2n\sqrt{E^2}}{16E^2+6/n}} \\
&\le \frac{\sqrt{n}\varepsilon'}{\sqrt{8}E^{1/2}}
\end{aligned} \tag{S287}$$

where (i) is a consequence of the fact that $\mathrm{Tr}[\hat{E}_n\rho]=\mathrm{Tr}[(\hat{E}_n^2)^{\frac{1}{2}}\rho]$ and exploited the concavity of $x\mapsto\sqrt{x}$ for $x>0$ (see Eq. (S7)). Furthermore, if (S285) holds, we have also that

$$\begin{aligned}
\|\tilde{\mathbf{m}}\tilde{\mathbf{m}}^{\intercal}-\mathbf{m}(\rho)\mathbf{m}(\rho)^{\intercal}\|_\infty &\le \|(\tilde{\mathbf{m}}-\mathbf{m}(\rho))\tilde{\mathbf{m}}^{\intercal}\|_\infty+\|\mathbf{m}(\rho)(\tilde{\mathbf{m}}-\mathbf{m}(\rho))^{\intercal}\|_\infty \\
&= \|\tilde{\mathbf{m}}-\mathbf{m}(\rho)\|_2\|\tilde{\mathbf{m}}\|_2+\|\mathbf{m}(\rho)\|_2\|\tilde{\mathbf{m}}-\mathbf{m}(\rho)\|_2 \\
&\le \|\tilde{\mathbf{m}}-\mathbf{m}(\rho)\|_2\left(\|\tilde{\mathbf{m}}-\mathbf{m}(\rho)\|_2+2\|\mathbf{m}(\rho)\|_2\right) \\
&\overset{\text{(ii)}}{\le} \frac{\sqrt{n}\varepsilon'}{\sqrt{8}E^{1/2}}\left(\frac{\sqrt{n}\varepsilon'}{\sqrt{8}E^{1/2}}+2\|\mathbf{m}(\rho)\|_2\right) \\
&\overset{\text{(iii)}}{\le} \frac{\sqrt{n}\varepsilon'}{\sqrt{8}E^{1/2}}\left(\frac{\sqrt{n}\varepsilon'}{\sqrt{8}E^{1/2}}+\sqrt{8n}E^{1/2}\right) \\
&= \left(\frac{\varepsilon'}{8E}+1\right)n\varepsilon' \\
&\overset{\text{(iv)}}{\le} 2n\varepsilon'.
\end{aligned} \tag{S288}$$

Here, in (ii) we have used (S287), while in (iii) we have exploited that

$$\|\mathbf{m}(\rho)\|_2=\sqrt{\sum_{i=1}^{2n}\mathrm{Tr}[\rho\hat{R}_i]^2}\le\sqrt{\sum_{i=1}^{2n}\mathrm{Tr}[\rho\hat{R}_i^2]}=\sqrt{2\,\mathrm{Tr}[\rho\hat{E}_n]}\le\sqrt{2\sqrt{\mathrm{Tr}[\rho\hat{E}_n^2]}}\le\sqrt{2n}E^{1/2}\,, \tag{S289}$$

where the first inequality and second inequality are a consequence of Eq. (S7). In (iv) we have used that the second moment satisfies $E\ge\frac{1}{2}$ (due to (S37)). Therefore, if (S284) and (S285) hold, then it holds that

$$\|\tilde{\mathbf{m}}-\mathbf{m}(\rho)\|_2\le\frac{\sqrt{n}\varepsilon'}{\sqrt{8}E^{1/2}} \tag{S290}$$

and

$$\|\tilde{V}-V(\rho)\|_\infty\le 5n\varepsilon'\,, \tag{S291}$$

where we have defined $\tilde{V}:=\tilde{W}-2\tilde{\mathbf{m}}\tilde{\mathbf{m}}^{\intercal}$ and we have used triangle inequality. Consequently, by setting $\varepsilon':=\frac{\varepsilon}{10n}$, we have that the choice

$$N'\ge 68\log\left(\frac{2(2n^2+3n)}{\delta}\right)\frac{200(8n^2E^2+3n)}{\varepsilon^2}\,, \tag{S292}$$

allows us to guarantee that

$$\Pr\left(\|\tilde{V}-V(\rho)\|_\infty\le\frac{\varepsilon}{2}\quad\text{and}\quad\|\tilde{\mathbf{m}}-\mathbf{m}(\rho)\|_2\le\frac{\varepsilon}{10\sqrt{8}E^{1/2}\sqrt{n}}\right)\ge 1-\delta\,. \tag{S293}$$

The total number of copies of ρ used in the algorithm in Table S2 is $N = (n+3)N'$. Then, the algorithm defines the matrix $\tilde{V}' := \tilde{V} + \frac{\varepsilon}{2}\mathbb{1}$. Consequently, in order to conclude the proof it suffices to show that if the condition $\|\tilde{V} - V(\rho)\|_\infty \le \frac{\varepsilon}{2}$ is satisfied, then $\tilde{V}'$ is a proper covariance matrix satisfying $\|\tilde{V}' - V(\rho)\|_\infty \le \varepsilon$. The latter condition follows by the triangle inequality. In order to show that $\tilde{V}'$ is a proper covariance matrix, let us observe that $\tilde{V}' + i\Omega_n \ge 0$, as

$$\begin{aligned}\tilde{V}' + i\Omega_n &= \tilde{V} + i\Omega_n + \frac{\varepsilon}{2}\mathbb{1}\\ &\overset{\text{(i)}}{\ge} \tilde{V} - V(\rho) + \frac{\varepsilon}{2}\mathbb{1}\\ &\overset{\text{(ii)}}{\ge} -\|\tilde{V} - V(\rho)\|_\infty\mathbb{1} + \frac{\varepsilon}{2}\mathbb{1}\\ &\ge 0\,,\end{aligned} \tag{S294}$$

where in (i) we have used that $V(\rho) + i\Omega_n \ge 0$, while in (ii) we have used that for any operator Θ it holds that $\Theta \ge -\|\Theta\|_\infty\mathbb{1}$. □

The sample complexity for estimating the covariance matrix of the above algorithm is $O\left(\frac{n^3E^2}{\varepsilon^2}\log\left(\frac{n^2}{\delta}\right)\right)$, outperforming the straightforward method of estimating indipendently the expectation values of all the observables $(\{\hat{R}_i, \hat{R}_j\})_{i,j\in[2n]}$, which leads to a worse sample complexity of $O\left(\frac{n^4E^2}{\varepsilon^2}\log\left(\frac{n^2}{\delta}\right)\right)$.

C. Learning Gaussian states

In this subsection, we analyse the algorithm for learning Gaussian states. In Subsubsection S3 C 1, we discuss the relation between the second moment energy constraint of a Gaussian state and its first moment and covariance matrix. Subsequently, in Subsubsection S3 C 2, we detail the algorithm for learning mixed Gaussian states and provide the associated recovery guarantees. Moreover, in Subsubsection S3 C 3, we rigorously show that this algorithm is noise-robust, if the state deviates slightly from the set of Gaussian states, the same tomography algorithm can still be applied. Finally, in Subsubsection S3 C 4, we outline the algorithm for learning pure Gaussian states and prove its recovery guarantees.

1. *Second moment of the energy of a Gaussian state*

A Gaussian state ρ is uniquely identified by its first moment $\mathbf{m}(\rho)$ and its covariance matrix $V(\rho)$. In the forthcoming lemma we will see how to express the second moment of the energy in terms of these quantities.

Lemma S55. *Let ρ be an n-mode Gaussian state with first moment $\boldsymbol{m}$ and covariance matrix V. The first and the second moment of the energy can be expressed in terms of the covariance matrix and the first moment as*

$$\mathrm{Tr}\left[\rho\hat{E}_n\right] = \frac{\mathrm{Tr}V}{4} + \frac{\|\boldsymbol{m}\|_2^2}{2}\,, \tag{S295}$$

$$\mathrm{Tr}\left[\rho\hat{E}_n^2\right] = \left(\frac{1}{4}\,\mathrm{Tr}\,V + \frac{1}{2}\|\boldsymbol{m}\|_2^2\right)^2 + \frac{1}{8}\,\mathrm{Tr}[V^2] + \frac{1}{2}\boldsymbol{m}^\intercal V\boldsymbol{m} - \frac{n}{4}\,, \tag{S296}$$

where $\hat{E}_n := \frac{\hat{\mathbf{R}}^\intercal\hat{\mathbf{R}}}{2}$ is the energy operator. In particular, this implies that the second moment of the energy can be upper bounded in terms of the mean energy as

$$\mathrm{Tr}[\rho\hat{E}_n^2] \le 3(\mathrm{Tr}[\rho\hat{E}_n])^2\,. \tag{S297}$$

Proof. (S295) holds because of Lemma S48. In order to prove (S296), let us employ [? , Lemma 7], which states that: for any $2n \times 2n$ real symmetric matrix X, it holds that

$$\mathrm{Tr}\left[(\hat{\mathbf{R}}^\intercal X\hat{\mathbf{R}})^2\,\rho(V,\mathbf{m})\right] = \left(\frac{1}{2}\,\mathrm{Tr}[VX] + \mathbf{m}^\intercal X\mathbf{m}\right)^2 + \frac{1}{2}\,\mathrm{Tr}[XVXV] + \frac{1}{2}\,\mathrm{Tr}[\Omega X\Omega X] + 2\mathbf{m}^\intercal XVX\mathbf{m}\,. \tag{S298}$$

Table S3. Estimation algorithm of an unknown n-mode Gaussian state ρ satisfying the energy constraint $\mathrm{Tr}[\rho\hat{E}_n] \le nE$.

Input: Accuracy ε, failure probability δ, mean energy per mode upper bound E, N of copies of the unknown n-mode Gaussian state ρ satisfying the energy constraint $\mathrm{Tr}[\rho\hat{E}_n] \le nE$, where

$$N := (n+3)\left\lceil 68\log\left(\frac{2(2n^2+3n)}{\delta}\right)\frac{200(24n^2E^2+3n)}{\varepsilon^4}2^{14}E^2n^4\right\rceil = O\left(\log\left(\frac{n^2}{\delta}\right)\frac{n^7E^4}{\varepsilon^4}\right). \tag{S300}$$

Output: With probability less than δ, the output is *failure*. Otherwise, with probability $\ge 1-\delta$, the output is a classical description of a Gaussian state $\tilde{\rho}$, such that

$$\frac{1}{2}\|\tilde{\rho}-\rho\|_1 \le \varepsilon\,. \tag{S301}$$

The classical description consists of the first moment $\mathbf{m}(\tilde{\rho}) \in \mathbb{R}^{2n}$ and in the covariance matrix $V(\tilde{\rho}) \in \mathbb{R}^{2n,2n}$, which uniquely characterise the Gaussian state $\tilde{\rho}$ and satisfy

$$\Pr\left(\|\tilde{V}-V(\rho)\|_\infty \le \frac{\varepsilon^2}{2^7En^2} \quad \text{and} \quad \|\tilde{\mathbf{m}}-\mathbf{m}(\rho)\|_2 \le \frac{\varepsilon^2}{2^7E^{3/2}n^{5/2}}\right) \ge 1-\delta\,. \tag{S302}$$

1: Query N copies of ρ and apply the algorithm in Table S2, which outputs, with probability $\ge 1-\delta$, a vector $\tilde{\mathbf{m}} \in \mathbb{R}^{2n}$ and a covariance matrix $\tilde{V} \in \mathbb{R}^{2n,2n}$. In case of failure of this algorithm, which happens with probability less than δ, declare *failure* and abort.
2: Define $\tilde{\rho}$ as the Gaussian state with first moment $\mathbf{m}(\tilde{\rho}) = \tilde{\mathbf{m}}$ and covariance matrix $V(\tilde{\rho}) = \tilde{V}$.
3: **return** the vector $\tilde{\mathbf{m}}$ and the matrix $\tilde{V}$, which form a classical description of the Gaussian state $\tilde{\rho}$.

By setting $X = \frac{\mathbb{1}}{2}$, this proves (S296). Moreover, note that

$$\begin{aligned}
\mathrm{Tr}\left[\rho\hat{E}_n^2\right] &= (\mathrm{Tr}[\rho\hat{E}_n])^2 + \frac{1}{8}\mathrm{Tr}[V^2] + \frac{1}{2}\mathbf{m}^\intercal V\mathbf{m} - \frac{n}{4} \\
&\le (\mathrm{Tr}[\rho\hat{E}_n])^2 + \frac{1}{8}[\mathrm{Tr}V]^2 + \frac{1}{2}\|\mathbf{m}\|_2^2\,\mathrm{Tr}V \\
&= (\mathrm{Tr}[\rho\hat{E}_n])^2 + \frac{1}{2}\mathrm{Tr}[\rho\hat{E}_n]\,\mathrm{Tr}V + \frac{1}{4}\|\mathbf{m}\|_2^2\,\mathrm{Tr}V \\
&= 3(\mathrm{Tr}[\rho\hat{E}_n])^2 - \mathrm{Tr}[\rho\hat{E}_n]\|\mathbf{m}\|_2^2 - \frac{1}{2}\|\mathbf{m}\|_2^4 \\
&\le 3(\mathrm{Tr}[\rho\hat{E}_n])^2\,,
\end{aligned} \tag{S299}$$

where we have used that $\mathrm{Tr}\left[\rho\hat{E}_n\right] = \frac{\mathrm{Tr}V}{4} + \frac{\|\mathbf{m}\|_2^2}{2}$. □

2. *Tomography algorithm for mixed Gaussian states*

In Table S3 we present a tomography algorithm to learn a classical description of an unknown n-mode Gaussian state ρ, by possessing the prior knowledge that ρ has mean energy per mode bounded by some known constant $E > 0$. The correctness of such a tomography algorithm is proved in Theorem S56. The idea of such a tomography algorithm is trivial: estimate first moment and covariance matrix, and then outputs the Gaussian state with these first moment and covariance matrix. The non-trivial aspect of the algorithm concerns the underlying sample complexity analysis, which crucially relies on upper bounding the error in trace distance in terms of the errors of first moment and covariance matrix. We have proved such an upper bound in Theorem S42 above.

Theorem S56. *Let $\varepsilon,\delta \in (0,1)$ and $E \ge 0$. Let ρ be an n-mode Gaussian state satisfying the energy constraint $\mathrm{Tr}[\rho\hat{E}_n] \le nE$. A number of copies N of ρ, such that*

$$\begin{aligned}
N &:= (n+3)\left\lceil 68\log\left(\frac{2(2n^2+3n)}{\delta}\right)\frac{200(24n^2E^2+3n)}{\varepsilon^4}2^{14}E^2n^4\right\rceil \\
&= O\left(\log\left(\frac{n^2}{\delta}\right)\frac{n^7E^4}{\varepsilon^4}\right),
\end{aligned} \tag{S303}$$

are sufficient to build a classical description of a Gaussian state $\tilde{\rho}$ *such that*

$$\Pr\left(\frac{1}{2}\|\tilde{\rho}-\rho\|_1 \le \varepsilon\right) \ge 1-\delta\,. \tag{S304}$$

The classical description consists of the first moment $\mathbf{m}(\tilde{\rho}) \in \mathbb{R}^{2n}$ *and in the covariance matrix* $V(\tilde{\rho}) \in \mathbb{R}^{2n,2n}$*, which uniquely characterise the Gaussian state* $\tilde{\rho}$ *and satisfy*

$$\Pr\left(\|\tilde{V}-V(\rho)\|_\infty \le \frac{\varepsilon^2}{2^7 E n^2} \quad \textit{and} \quad \|\tilde{\mathbf{m}}-\mathbf{m}(\rho)\|_2 \le \frac{\varepsilon^2}{2^7 E^{3/2} n^{5/2}}\right) \ge 1-\delta\,. \tag{S305}$$

Proof. We aim to establish the correctness of the algorithm presented in Table S3. First, let us observe that the second moment of the energy of the Gaussian state ρ, which satisfies the energy constraint $\mathrm{Tr}[\rho\hat{E}_n] \le nE$, can be upper bounded as

$$\frac{1}{n}\sqrt{\mathrm{Tr}[\rho\hat{E}_n^2]} \le \sqrt{3}\frac{\mathrm{Tr}[\rho\hat{E}_n]}{n} \le \sqrt{3}E\,, \tag{S306}$$

where we have used Lemma S55. Consequently, for any $\varepsilon' \in (0,\frac{1}{2})$ Theorem S53 establishes that a number

$$N \coloneqq (n+3)\left\lceil 68\log\left(\frac{2(2n^2+3n)}{\delta}\right)\frac{200(24n^2E^2+3n)}{\varepsilon'^2}\right\rceil \tag{S307}$$

of copies of ρ suffices to construct a vector $\tilde{\mathbf{m}} \in \mathbb{R}^{2n}$ and a covariance matrix $\tilde{V} \in \mathbb{R}^{2n,2n}$ such that

$$\Pr\left(\|\tilde{V}-V(\rho)\|_\infty \le \varepsilon' \quad \text{and} \quad \|\tilde{\mathbf{m}}-\mathbf{m}(\rho)\|_2 \le \frac{\varepsilon'}{10(3)^{1/4}\sqrt{8En}}\right) \ge 1-\delta\,. \tag{S308}$$

Let $\tilde{\rho}$ be the Gaussian state with first moment $\mathbf{m}(\tilde{\rho}) = \tilde{\mathbf{m}}$ and covariance matrix $V(\tilde{\rho}) = \tilde{V}$. Let us show that, with probability at least $1-\delta$, the energy of the estimator $\tilde{\rho}$ is at most twice the energy of the true state ρ, since

$$\begin{aligned}
\mathrm{Tr}[\tilde{\rho}\hat{E}_n] &\overset{\text{(i)}}{=} \frac{\mathrm{Tr}\,\tilde{V}}{4} + \frac{\|\tilde{\mathbf{m}}\|_2^2}{2} \\
&\overset{\text{(ii)}}{\le} \frac{\mathrm{Tr}\,V(\rho)}{4} + \frac{2n\|\tilde{V}-V(\rho)\|_\infty}{4} + \frac{(\|\tilde{\mathbf{m}}-\mathbf{m}(\rho)\|_2 + \|\mathbf{m}(\rho)\|_2)^2}{2} \\
&\overset{\text{(iii)}}{\le} \mathrm{Tr}[\rho\hat{E}_n] + \frac{n\varepsilon'}{2} + \frac{\left(\frac{\varepsilon'}{10(3)^{1/4}\sqrt{8En}}\right)^2 + 2\|\mathbf{m}(\rho)\|_2\left(\frac{\varepsilon'}{10(3)^{1/4}\sqrt{8En}}\right)}{2} \\
&\le \mathrm{Tr}[\rho\hat{E}_n] + \frac{n\varepsilon'}{2} + \frac{1+2\|\mathbf{m}(\rho)\|_2}{2}\left(\frac{\varepsilon'}{10(3)^{1/4}\sqrt{8En}}\right) \\
&\overset{\text{(iv)}}{\le} \mathrm{Tr}[\rho\hat{E}_n] + \frac{n\varepsilon'}{2} + \frac{1+2\sqrt{2nE}}{2}\left(\frac{\varepsilon'}{10(3)^{1/4}\sqrt{8En}}\right) \\
&\le \mathrm{Tr}[\rho\hat{E}_n] + \frac{n\varepsilon'}{2} + \frac{\varepsilon'}{10(3)^{1/4}} \\
&\le \mathrm{Tr}[\rho\hat{E}_n] + n\varepsilon' \\
&\le \mathrm{Tr}[\rho\hat{E}_n] + \frac{n}{2} \\
&\le 2\,\mathrm{Tr}[\rho\hat{E}_n]\,.
\end{aligned} \tag{S309}$$

Here, in (i), we have exploited Lemma S48. In (ii), we have used Hölder inequality, triangle inequality, and the fact that $\|\tilde{V}-V(\rho)\|_\infty \le \varepsilon'$. In (iii), we have exploited again Lemma S48 and we have used that $\|\tilde{\mathbf{m}}-\mathbf{m}(\rho)\|_2 \le \frac{\varepsilon'}{10(3)^{1/4}\sqrt{8En}}$. The

inequality in (iv) follows from (S289). Then, building on Theorem S42, we obtain that, with probability at least $1-\delta$, it holds that

$$
\begin{aligned}
\frac{1}{2}\left\|\rho-\tilde{\rho}\right\|_1 &\le 2\sqrt{\max\left(\mathrm{Tr}[\rho\hat{E}_n],\mathrm{Tr}[\tilde{\rho}\hat{E}_n]\right)}\left(\|\mathbf{m}(\rho)-\mathbf{m}(\tilde{\rho})\|_2+\sqrt{2}\sqrt{\|V(\rho)-V(\tilde{\rho})\|_1}\right)\\
&\le 2\sqrt{2nE}\left(\|\mathbf{m}(\rho)-\mathbf{m}(\tilde{\rho})\|_2+\sqrt{2}\sqrt{\|V(\rho)-V(\tilde{\rho})\|_1}\right)\\
&\le \sqrt{8nE}\left(\frac{\varepsilon'}{10(3)^{1/4}\sqrt{8En}}+2\sqrt{n\|V(\rho)-V(\tilde{\rho})\|_\infty}\right)\\
&\le \sqrt{8nE}\left(\frac{\varepsilon'}{10(3)^{1/4}\sqrt{8En}}+2\sqrt{n\varepsilon'}\right)\\
&\le \sqrt{8nE}\left(4\sqrt{n\varepsilon'}\right)\\
&= \sqrt{2^7En^2\varepsilon'}\,.
\end{aligned}
\tag{S310}
$$

Consequently, by setting $\varepsilon' := \frac{\varepsilon^2}{2^7En^2}\in(0,\frac{1}{2})$, we have that the choice

$$
N := \ (n+3)\left\lceil 68\log\left(\frac{2(2n^2+3n)}{\delta}\right)\frac{200(24n^2E^2+3n)}{\varepsilon^4}2^{14}E^2n^4\right\rceil \tag{S311}
$$

allows us to guarantee that

$$
\Pr\left(\frac{1}{2}\|\tilde{\rho}-\rho\|_1\le\varepsilon\right)\ge 1-\delta\,. \tag{S312}
$$

With this choice of ε', we have also that

$$
\Pr\left(\|\tilde{V}-V(\rho)\|_\infty\le\frac{\varepsilon^2}{2^7En^2}\quad\text{and}\quad\|\tilde{\mathbf{m}}-\mathbf{m}(\rho)\|_2\le\frac{\varepsilon^2}{10(3)^{1/4}2^7\sqrt{8}E^{3/2}n^{5/2}}\right)\ge 1-\delta\,, \tag{S313}
$$

which, in particular, implies that

$$
\Pr\left(\|\tilde{V}-V(\rho)\|_\infty\le\frac{\varepsilon^2}{2^7En^2}\quad\text{and}\quad\|\tilde{\mathbf{m}}-\mathbf{m}(\rho)\|_2\le\frac{\varepsilon^2}{2^7E^{3/2}n^{5/2}}\right)\ge 1-\delta\,. \tag{S314}
$$

□

3. *Noise robustness of the algorithm*

Above we have seen that learning an unknown Gaussian states is efficient, as the sample complexity scales polynomially in the number of modes. Here, we address the questions: *What happens if the state is not exactly Gaussian? Is our tomography procedure stable under little perturbations of the set of Gaussian states?* Answering these questions is conceptually relevant, as experimental imperfections may transform Gaussian states into slightly-perturbed Gaussian states.

Here, in order to quantify the non-Gaussian character of a quantum state, we use the *relative entropy of non-Gaussianity* [**?** **?**]. For any state ρ, the relative entropy of non-Gaussianity $d_{\mathscr{G}}(\rho)$ is defined as the minimum relative entropy between ρ and any Gaussian state

$$
d_{\mathscr{G}}(\rho) := \min_{\sigma\in\mathscr{G}} S(\rho\|\sigma)\,, \tag{S315}
$$

where $\mathscr{G}$ denotes the set of Gaussian states and

$$
S(\rho\|\sigma) := \mathrm{Tr}[\rho\log_2\rho]-\mathrm{Tr}[\rho\log_2\sigma] \tag{S316}
$$

denotes the quantum relative entropy between ρ and σ. The quantum relative entropy $S(\rho\|\sigma)$ has a strong operation meaning in terms of the optimal rate in asymmetric quantum hypothesis testing between ρ and σ, due to the quantum Stein's lemma [**?** **?**].

As proved by [**?**], the relative entropy of non-Gaussianity satisfies desirable properties that make it a meaningful measure of non-Gaussianity. In particular, note that $d_G(\rho)$ is faithful, in the sense that $d_G(\rho)\ge 0$ and it vanishes if and only if ρ is Gaussian. Notably, the minimum in the definition of $d_{\mathscr{G}}(\rho)$ is achieved by the *Gaussianification* of ρ [**?**]. The Gaussianification $G(\rho)$

of a state ρ is the Gaussian state with the same first moment and covariance matrix of ρ. In formula, the relative entropy of non-Gaussianity is given by [?]:

$$d_{\mathscr{G}}(\rho) = S(\rho \| G(\rho)) \ . \tag{S317}$$

Let us now analyse the stability of our tomography algorithm for Gaussian states. We make use the of the following simple observation.

Lemma S57. *If the unknown state ρ is not Gaussian, the algorithm designed for learning Gaussian states in Table S3 effectively learns the Gaussianification $G(\rho)$. Mathematically, $O\left(\log\left(\frac{n^2}{\delta}\right)\frac{n^7E^4}{\varepsilon^4}\right)$ copies of ρ suffices in order to build a classical description of a Gaussian state $\tilde{\rho}$ such that $\Pr\left(\frac{1}{2}\|\tilde{\rho} - G(\rho)\|_1 \le \varepsilon\right) \ge 1-\delta$.*

Proof. The claim follows by two main observations: (i) the algorithm in Table S3 only involves the first moment and the covariance matrix of the unknown state, and (ii) the mean photon number of a state ρ and the one of its Gaussianification $G(\rho)$ coincide (because of Lemma S48). □

Now we show that *quantum state tomography of slightly-perturbed Gaussian states is efficient*. Here, by 'slightly-perturbed Gaussian state' we mean that the relative entropy of non-Gaussianity is sufficiently small.

Theorem S58 (Quantum state tomography of slightly-perturbed Gaussian states is efficient)**.** *Let $\varepsilon, \delta \in (0,1)$ and $E \ge 0$. Let ρ be an unknown n-mode (possibly non-Gaussian) state such that its relative entropy of non-Gaussianity satisfies $d_{\mathscr{G}}(\rho) \le \varepsilon^2$. Assume that ρ satisfies the energy constraint $\mathrm{Tr}[\rho \hat{E}_n] \le nE$. Then, $O\left(\log\left(\frac{n^2}{\delta}\right)\frac{n^7E^4}{\varepsilon^4}\right)$ copies of ρ suffices to build a classical description of a Gaussian state $\tilde{\rho}$ such that $\Pr\left(\frac{1}{2}\|\tilde{\rho} - \rho\|_1 \le \varepsilon\right) \ge 1-\delta$.*

Proof. Thanks to Remark S57, $O\left(\log\left(\frac{n^2}{\delta}\right)\frac{n^7E^4}{\varepsilon^4}\right)$ copies of ρ suffices to build a classical description of a Gaussian state $\tilde{\rho}$ such that $\frac{1}{2}\|\tilde{\rho} - G(\rho)\|_1 \le \left(1 - \sqrt{\frac{\ln 2}{2}}\right)\varepsilon$ with probability at least $1-\delta$, where $G(\rho)$ denotes the Gaussianification of ρ. If this event happens, then

$$\begin{aligned}
\frac{1}{2}\|\rho - \tilde{\rho}\|_1 &\le \frac{1}{2}\|G(\rho) - \tilde{\rho}\|_1 + \frac{1}{2}\|G(\rho) - \rho\|_1 \\
&\le \left(1 - \sqrt{\frac{\ln 2}{2}}\right)\varepsilon + \frac{1}{2}\|G(\rho) - \rho\|_1 \\
&\overset{\text{(i)}}{\le} \left(1 - \sqrt{\frac{\ln 2}{2}}\right)\varepsilon + \sqrt{\frac{\ln 2}{2}}\sqrt{S(\rho\|G(\rho))} \\
&\overset{\text{(ii)}}{=} \left(1 - \sqrt{\frac{\ln 2}{2}}\right)\varepsilon + \sqrt{\frac{\ln 2}{2}}\sqrt{d_{\mathscr{G}(\rho)}} \\
&\le \varepsilon \, .
\end{aligned} \tag{S318}$$

Here, in (i), we employed the quantum Pinsker inequality [? , Theorem 11.9.1], which states that for any τ and σ the trace distance can be upper bounded in terms of the relative entropy as $\frac{1}{2}\|\tau - \sigma\|_1 \le \sqrt{\frac{\ln 2}{2} S(\tau\|\sigma)}$. Finally, in (ii), we just employed the characterisation of the relative entropy of non-Gaussianity in (S317). □

4. *Improved tomography algorithm for pure Gaussian states*

In this subsubsection we present an improved upper bound on the sample complexity of tomography of pure Gaussian states. We can show that $O\left(\log\left(\frac{n^2}{\delta}\right)\frac{n^5E^4}{\varepsilon^4}\right)$ state copies suffices to achieve tomography of pure Gaussian states, thus obtaining an improvement with respect the scaling $O\left(\log\left(\frac{n^2}{\delta}\right)\frac{n^7E^4}{\varepsilon^4}\right)$ of the mixed setting analysed in subsubsection S3 C 2. The main technical tool employed here is the improved upper bound on the trace distance between a pure Gaussian state and an arbitrary (possibly-mixed) state presented in Theorem S49.

Theorem S59 (Tomography of pure Gaussian states)**.** *Let $\varepsilon,\delta\in(0,1)$ and $E\geq 0$. Let ψ be an n-mode pure Gaussian state satisfying the energy constraint $\mathrm{Tr}[\psi\hat{E}_n]\leq nE$. A number of copies N of ψ, such that*

$$\begin{aligned} N \coloneqq\ & (n+3)\left\lceil 68\log\left(\frac{2(2n^2+3n)}{\delta}\right)\frac{200(24n^2E^2+3n)}{\varepsilon^4}16E^2n^2\right\rceil \\ &= O\left(\log\left(\frac{n^2}{\delta}\right)\frac{n^5E^4}{\varepsilon^4}\right), \end{aligned} \tag{S319}$$

are sufficient to build a classical description of a Gaussian state $\tilde{\rho}$ such that

$$\Pr\left(\frac{1}{2}\|\tilde{\rho}-\psi\|_1\leq\varepsilon\right)\geq 1-\delta\,. \tag{S320}$$

Proof. We proceed as in Lemma S56. First, let us observe that Lemma S55 establishes that the second moment of the energy of the Gaussian state ψ, which satisfies the energy constraint $\mathrm{Tr}[\psi\hat{E}_n]\leq nE$, can be upper bounded as

$$\frac{1}{n}\sqrt{\mathrm{Tr}[\psi\hat{E}_n^2]}\leq\sqrt{3}\frac{\mathrm{Tr}[\psi\hat{E}_n]}{n}\leq\sqrt{3}E\,. \tag{S321}$$

Consequently, for any $\varepsilon'\in(0,\frac{1}{2})$ Theorem S53 establishes that a number

$$N \coloneqq\ (n+3)\left\lceil 68\log\left(\frac{2(2n^2+3n)}{\delta}\right)\frac{200(24n^2E^2+3n)}{\varepsilon'^2}\right\rceil \tag{S322}$$

of copies of ψ suffices to construct a vector $\tilde{\mathbf{m}}\in\mathbb{R}^{2n}$ and a covariance matrix $\tilde{V}\in\mathbb{R}^{2n,2n}$ such that

$$\Pr\left(\|\tilde{V}-V(\psi)\|_\infty\leq\varepsilon'\quad\text{and}\quad\|\tilde{\mathbf{m}}-\mathbf{m}(\psi)\|_2\leq\frac{\varepsilon'}{10(3)^{1/4}\sqrt{8En}}\right)\geq 1-\delta\,. \tag{S323}$$

Let $\tilde{\rho}$ be the Gaussian state with first moment $\mathbf{m}(\tilde{\rho})=\tilde{\mathbf{m}}$ and covariance matrix $V(\tilde{\rho})=\tilde{V}$. By exploiting the same reasoning used in (S309), we have that the energy of the estimator $\tilde{\rho}$ is at most twice the energy of ψ, i.e.

$$\mathrm{Tr}[\tilde{\rho}\hat{E}_n]\leq 2\,\mathrm{Tr}[\psi\hat{E}_n]\,, \tag{S324}$$

with probability at least $1-\delta$. Then, by exploiting Theorem S49, we have that, with probability at least $1-\delta$, it holds that

$$\begin{aligned} \frac{1}{2}\|\,\tilde{\rho}-\psi\,\|_1 &\leq\sqrt{\max\left(\mathrm{Tr}[\tilde{\rho}\hat{E}_n],\mathrm{Tr}[\psi\hat{E}_n]\right)}\sqrt{\|V(\tilde{\rho})-V(\psi)\|_\infty+2\|\mathbf{m}(\tilde{\rho})-\mathbf{m}(\psi)\|_2^2} \\ &\leq\sqrt{2nE}\sqrt{\|V(\tilde{\rho})-V(\psi)\|_\infty+2\|\mathbf{m}(\tilde{\rho})-\mathbf{m}(\psi)\|_2^2} \\ &\leq\sqrt{2nE}\sqrt{\varepsilon'+\frac{\varepsilon'^2}{400\sqrt{3}\,En}} \\ &\leq\sqrt{4nE\varepsilon'}\,. \end{aligned} \tag{S325}$$

Consequently, by setting $\varepsilon'\coloneqq\frac{\varepsilon^2}{4nE}\in(0,\frac{1}{2})$, we have that the choice

$$N \coloneqq\ (n+3)\left\lceil 68\log\left(\frac{2(2n^2+3n)}{\delta}\right)\frac{200(24n^2E^2+3n)}{\varepsilon^4}16E^2n^2\right\rceil \tag{S326}$$

allows us to guarantee that

$$\Pr\left(\frac{1}{2}\|\tilde{\rho}-\psi\|_1\leq\varepsilon\right)\geq 1-\delta\,. \tag{S327}$$

□

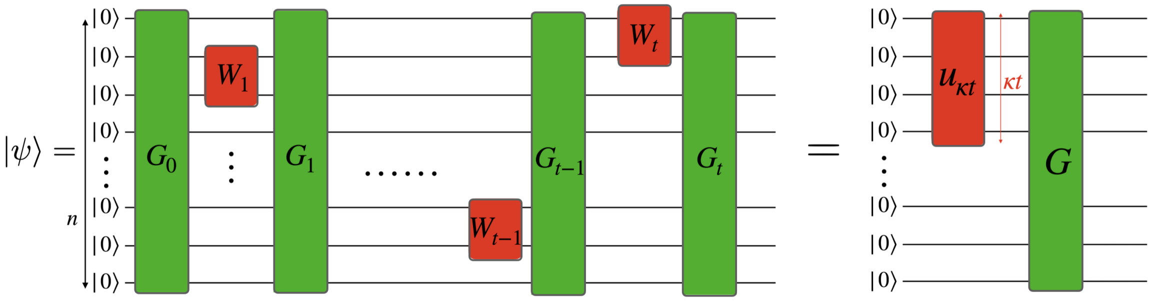

Figure S4. Depiction of the decomposition of a t-doped Gaussian state vector $|\psi\rangle$ proved in Theorem S60. The t-doped Gaussian state vector $|\psi\rangle$ is prepared by applying Gaussian unitaries (green gates) and at most t local non-Gaussian unitaries (red gates). In the specific example depicted in the figure, the locality is $\kappa = 4$ as each of the non-Gaussian unitaries acts non-trivially on two modes (thus 4 quadratures). The compression statement amounts to the insight that all the 'non-Gaussianity' of $|\psi\rangle$ can be compressed on the first κt modes by applying to $|\psi\rangle$ a suitable Gaussian unitary $G^\dagger$.

S4. TOMOGRAPHY OF t-DOPED BOSONIC GAUSSIAN STATES

In the preceding section, we rigorously proved that quantum state tomography of bosonic Gaussian states is efficient. In this section, we delve into the analysis of *t-doped bosonic Gaussian states*, which are states prepared by Gaussian unitaries and at most t non-Gaussian local unitaries. They encompass a much broader class of efficiently learnable states than exact bosonic Gaussian states.

The results we will show in this section can be seen as an extension to the bosonic setting of what was previously shown for t-doped stabiliser states [**? ? ?**] (states prepared by Clifford gates and at most t T-gates) and t-doped fermionic Gaussian states of Ref. [**?**] (states prepared by fermionic Gaussian unitaries and at most t fermionic non-Gaussian local unitaries). However, extending these results is far from trivial, as in the bosonic setting one must deal not only with different commutation relations than in the fermionic setting, but also with subtleties arising from the energy constraints and from the infinite-dimensional Hilbert space. Just as T-gates are considered *magic* gates for Clifford circuits, which are classically simulable [**?**], local non-Gaussian gates can similarly be viewed as *magic* gates for Gaussian circuits, which are also classically simulable [**?**]. Recent works have also been focusing on the classical simulability of states prepared by a Gaussian evolution applied to an input state that is a superposition of a bounded number of Gaussian states [**? ? ?**].

An n-mode unitary U is said to be a *(t,κ)-doped Gaussian unitary* if it is a composition of Gaussian unitaries and at most t non-Gaussian κ-local unitaries, where κ-local means that each non-Gaussian gate involves at most κ quadrature operators. In other words, U is of the form

$$U = G_t W_t \cdots G_1 W_1 G_0 \,, \tag{S328}$$

where each G_i is an n-mode Gaussian unitary and W_i is a κ-local non-Gaussian unitary. Strictly speaking, we assume each W_i to be a unitary generated by a Hamiltonian which is a (non-quadratic) polynomial in at most κ quadrature operators. An n-mode state vector $|\psi\rangle$ is said to be a *(t,κ)-doped Gaussian state* if it can be prepared by applying a (t,κ)-doped Gaussian unitary to the vacuum, that is,

$$|\psi\rangle = U\,|0\rangle^{\otimes n} \,. \tag{S329}$$

Informally, we sometimes say that a state is a t-doped Gaussian state (by omitting the dependence on κ) if it is a (t,κ)-doped Gaussian state for $\kappa = O(1)$ in the number of modes. The left hand side of Fig. S4 shows a depiction of a t-doped Gaussian state.

The forthcoming Theorem S60 provides a remarkable decomposition of (t,κ)-doped unitaries and states, which shows that, if $\kappa t \le n$, one can turn any t-doped state into a tensor product between a κt-mode non-Gaussian state and the $(n-\kappa t)$-mode vacuum state via a suitable Gaussian unitary. We provide a depiction of such a decomposition in Fig. S4

Theorem S60 (Non-Gaussianity compression in t-doped Gaussian unitaries and states)**.** *If $n \ge \kappa t$, any n-mode (t,κ)-doped Gaussian unitary U can be decomposed as*

$$U = G(u_{\kappa t} \otimes \mathbb{1}_{n-\kappa t}) G_{passive} \tag{S330}$$

for some suitable Gaussian unitary G, passive Gaussian unitary $G_{passive}$, and κt-mode (non-Gaussian) unitary $u_{\kappa t}$. In particular, any n-mode (t,κ)-doped Gaussian state can be decomposed as

$$|\psi\rangle = G\left(|\phi_{\kappa t}\rangle \otimes |0\rangle^{\otimes(n-\kappa t)}\right) \tag{S331}$$

for some suitable Gaussian unitary G and κt-mode (non-Gaussian) state vector $|\phi_{\kappa t}\rangle$.

The decomposition in (S331) shows that it is possible to compress all the non-Gaussianity of a t-doped Gaussian state via a suitable Gaussian unitary. By leveraging such a property, we devise a tomography algorithm for t-doped Gaussian states which has a sample and time complexity that scales polynomially in n as long as $\kappa t = O(1)$, *thereby establishing that tomography of (energy-constrained) t-doped states is efficient* in this regime. This establishes the robustness of tomography of Gaussian states, in the sense that, even if few non-Gaussian unitaries are applied to a Gaussian state, the resulting state remains efficiently learnable.

The rough idea behind our tomography algorithm for unknown t-doped Gaussian states $|\psi\rangle$ involves first estimating the Gaussian unitary G (see (S331)), then applying its inverse to the state in order to compress the non-Gaussianity, and finally performing tomography of the first κt modes. Remarkably, our tomography algorithm is experimentally feasible, as it uses only Gaussian unitaries and easily implementable Gaussian measurements, such as homodyne and heterodyne detection [**? ?**].

In the following theorem, we analyse the performance guarantees of our tomography algorithm.

Theorem S61 (Informal version)**.** *Let $|\psi\rangle$ be an unknown n-mode (t,κ)-doped Gaussian state vector, which satisfies the second-moment constraint $\mathrm{Tr}[\psi \hat{N}_n^2] \le n^2 N_{phot}^2$. Then, $O\left(\left(\frac{n^2 N_{phot}^2}{\varepsilon^2}\right)^{\kappa t}\right)$ state copies suffices to construct a succint classical description of an estimator $|\tilde{\psi}\rangle$ which is ε-close in trace distance to $|\psi\rangle$ with high probability. Such a classical description consists of a triplet $(\tilde{\boldsymbol{m}}, \tilde{S}, |\tilde{\phi}_1\rangle)$, which defines the estimator via the relation*

$$|\tilde{\psi}\rangle := \hat{D}_{\tilde{\boldsymbol{m}}} U_{\tilde{S}} |\tilde{\phi}_1\rangle \otimes |0\rangle^{\otimes(n-\kappa t)}\,, \tag{S332}$$

where $\hat{D}_{\tilde{\boldsymbol{m}}}$ is the displacement operator with $\tilde{\boldsymbol{m}} \in \mathbb{R}^{2n}$, $U_{\tilde{S}}$ is a Gaussian unitary associated with the $2n \times 2n$ symplectic matrix $\tilde{S}$, and $|\tilde{\phi}_1\rangle$ is a κt-mode state contained in the d_{eff}-dimensional subspace spanned by all the n-mode Fock states with total photon number less than $O\left(\left(\frac{n^2 N_{phot}^2}{\varepsilon^2}\right)\right)$, where

$$d_{eff} := O\left(\left(\frac{n^2 N_{phot}^2}{\varepsilon^2}\right)^{\kappa t}\right)\,. \tag{S333}$$

In particular, tomography of t-doped Gaussian states is efficient in the regime $\kappa t = O(1)$, as its sample, time, and memory complexity scales polynomially in n.

We conclude by showing that any tomography algorithm that aims to learn quantum states which can be written as $G(|\phi_t\rangle \otimes |0\rangle^{\otimes(n-t)})$, where G is a Gaussian unitary and $|\phi_t\rangle$ a t-mode quantum state vector, must be inefficient if t scales *slightly* more than a constant in the number of modes.

A. t-doped Gaussian states and unitaries

We now present the formal definitions crucial for our analysis.

Definition S62 (Non-Gaussian k-local unitary)**.** *Let $n \in \mathbb{N}$ and $\kappa \in [2n]$. A* κ-local non-Gaussian unitary *is an n-mode unitary generated by a Hamiltonian which is a polynomial in at most k quadrature operators $\{\hat{R}_{\mu(r)}\}_{r=1}^{\kappa}$, where $\mu(1), \ldots, \mu(\kappa) \in [2n]$.*

For instance, unitaries like $\exp\left[i\hat{x}_1^4\right]$, $\exp\left[i(\hat{x}_1^4 + \hat{p}_1^4)\right]$, $\exp\left[i(\hat{x}_1^4 + \hat{p}_1^4 + \hat{x}_3^6)\right]$, and $\exp\left[i0.7\hat{a}_1^\dagger \hat{a}_2^\dagger \hat{a}_3 \hat{a}_4\right]$ are examples of 1-, 2-, 3-local, and 4-local non-Gaussian unitaries, respectively.

Definition S63 (t-doped Gaussian unitary)**.** *A unitary U_t is a (t,κ)-doped Gaussian unitary if it can be prepared by Gaussian unitaries $\{G_i\}_{i=0}^{t}$ and t non-Gaussian κ-local unitaries $\{W_i\}_{i=1}^{t}$, specifically given by*

$$U_t = G_t W_t \cdots G_1 W_1 G_0\,. \tag{S334}$$

Definition S64 (t-doped Gaussian state)**.** *A (t,κ)-doped Gaussian state vector $|\psi\rangle$ is obtained by the action of a (t,κ)-doped Gaussian unitary U_t on the vacuum, i.e., $|\psi\rangle := U_t |0\rangle^{\otimes n}$.*

B. Compression of non-Gaussianity

In this section, we introduce a finding that sheds light on the structure of t-doped Gaussian unitaries: the 'non-Gaussianity' can be compressed into the first modes through a Gaussian operation. This result is an extension to the bosonic setting of a result

proved in Ref. [**?**], formulated for t-doped *fermionic* unitaries. The proof for bosons can be easily generalised from the one for fermions of Ref. [**?**], by making use of the following parallelism between the theory of bosonic Gaussian and fermionic Gaussian unitaries. Specifically, in the bosonic setting, Gaussian unitaries correspond to symplectic matrices, and Gaussian passive unitaries are associated with symplectic-orthogonal matrices. In the fermionic context, the role of symplectic and orthogonal matrices is exchanged: Gaussian unitaries correspond to orthogonal matrices, and Gaussian passive unitaries are associated with symplectic-orthogonal matrices. Consequently, when handling Gaussian passive transformations, the theory of Gaussian bosons aligns with the theory of Gaussian fermions. In the following proof, we follow the construction presented in Ref. [**?**].

Theorem S65 (Compression of non-Gaussianity in t-doped unitaries)**.** *Let $n \in \mathbb{N}$ be the number of modes. Let $\kappa, t \in N$ such that $\kappa t \leq n$. For any (t,κ)-doped bosonic Gaussian unitary U_t there exist a Gaussian unitary G, a passive Gaussian unitary $G_{passive}$, and a (possibly non-Gaussian) unitary $u_{\kappa t}$ supported exclusively on the first κt modes such that*

$$U_t = G(u_{\kappa t} \otimes \mathbb{1}_{n-\kappa t})G_{passive}, \tag{S335}$$

where $\mathbb{1}_{n-\kappa t}$ denotes the identity on the last $n - \kappa t$ modes.

Proof. By definition, the t-doped unitary can be expressed as $U_t = (\prod_{t'=1}^{t} G_{t'} W_{t'})G_0$. Rearranging it, we express it as

$$U_t = \tilde{G}_t \prod_{t'=1}^{t} \tilde{W}_{t'}, \tag{S336}$$

where $\tilde{W}_{t'} := \tilde{G}^{\dagger}_{t'-1} W_{t'} \tilde{G}_{t'-1}$ and $\tilde{G}_{t'} := G_{t'}..G_0$. Let G_{aux} be a passive Gaussian unitary, which we will fix later in order to compress all the non-Gaussianity to the first κt modes. We can rewrite U_t as

$$U_t = \tilde{G}_t G_{\text{aux}} \prod_{t'=1}^{t} (G^{\dagger}_{\text{aux}} \tilde{W}_{t'} G_{\text{aux}}) G^{\dagger}_{\text{aux}} \,. \tag{S337}$$

Moreover, by defining

$$\begin{aligned} G &:= \tilde{G}_t G_{\text{aux}} \,, \\ u_{\kappa t} &:= \prod_{t'=1}^{t} (G^{\dagger}_{\text{aux}} \tilde{W}_{t'} G_{\text{aux}}) \,, \\ G_{\text{passive}} &:= G^{\dagger}_{\text{aux}} \,, \end{aligned} \tag{S338}$$

we have

$$U_t = G u_{\kappa t} G_{\text{passive}} \,. \tag{S339}$$

Observe that G is a Gaussian unitary, as the product of Gaussian unitaries remains Gaussian. Similarly, G_{passive} is a passive Gaussian unitary, given that the adjoint of a passive Gaussian unitary remains passive and Gaussian. In order to conclude the proof, we have to show that we can choose G_{aux} such that $u_{\kappa t}$ is supported only on the first κt modes. It suffices to show that the Heisenberg evolution, under the Gaussian unitary $\tilde{G}_{t'-1} G_{\text{aux}}$, of the Hamiltonian generating $W_{t'}$ involves only the first κt quadratures $(\hat{R}_i)_{i \in [\kappa t]}$. In other words, we need to show that the Heisenberg evolution of each quadrature operator involved in the Hamiltonian generating $W_{t'}$ involves only the first κt quadratures. Let O_{aux} be the symplectic orthogonal matrix associated with G_{aux}. For each $t' \in [t]$ let $\mu(t',1), \mu(t',2), \ldots, \mu(t',\kappa)$, with $\mu(t',1) \leq \mu(t',2) \leq \ldots \leq \mu(t',\kappa)$, be the quadratures involved in the Hamiltonian generating $W_{t'}$. For each $t' \in [t]$ and $r \in [\kappa]$ it holds that

$$G^{\dagger}_{\text{aux}} \tilde{G}^{\dagger}_{t'-1} \hat{R}_{\mu(t',r)} \tilde{G}_{t'-1} G_{\text{aux}} = \sum_{m=1}^{2n} (S_{t'-1} O_{\text{aux}})_{\mu(t',r),m} \hat{R}_m \,, \tag{S340}$$

where $S_{t'-1}$ is the symplectic matrix associated with $\tilde{G}_{t'-1}$. Consequently, we have to choose O_{aux} so that

$$(S_{t'-1} O_{\text{aux}})_{\mu(t',r),m} = (O^{\intercal}_{\text{aux}} S^{\intercal}_{t'-1})_{m,\mu(t',r)} = 0 \tag{S341}$$

for all $t' \in [t]$, $r \in [\kappa]$, and all $m \in \{2\kappa t + 1, \ldots, 2n\}$. Let $(\mathbf{e}_i)_{i \in [2n]}$ be the canonical basis vectors of $\mathbb{R}^{2n}$. For simplicity, let us denote the vectors $(S^{\intercal}_{t'-1} \mathbf{e}_{\mu(t',r)})_{t' \in [t], r \in [\kappa]}$ with $(\mathbf{v}_j)_{j \in [\kappa t]}$. It suffices to show that there exists an orthogonal symplectic matrix O_{aux} such that for all $j \in [\kappa t]$ and all $m \in \{2\kappa t + 1, \ldots, 2n\}$ it holds that

$$\mathbf{e}^T_m O^{\intercal}_{\text{aux}} \mathbf{v}_j = 0 \,. \tag{S342}$$

For any arbitrary vectors $\{\mathbf{v}_j\}_{j \in [\kappa t]}$, there exist such an orthogonal matrix O_{aux}. This is a consequence of the forthcoming Lemma S66, pointed out in Ref. [**?**], which is a consequence of the well-known isomorphism between $2n \times 2n$ symplectic orthogonal real matrices and $n \times n$ unitaries [**?**]. □

Lemma S66. *Let $\{\mathbf{e}_i\}_{i=1}^{2n}$ be the canonical basis of $\mathbb{R}^{2n}$. Let $\mathbf{v}_1, \ldots, \mathbf{v}_M \in \mathbb{R}^{2n}$ be a set of real vectors, where $M \leq 2n$. There exists an orthogonal symplectic matrix $O \in \mathrm{O}(2n) \cap \mathrm{Sp}(2n, \mathbb{R})$ such that*

$$\mathbf{e}_i^T O \mathbf{v}_j = 0, \tag{S343}$$

for all $i \in \{2M+1, \ldots, 2n\}$ and $j \in [M]$, meaning that all $\{O\mathbf{v}_j\}_{j=1}^{M}$ are exclusively supported on the span of the first $2M$ canonical basis vectors.

The proof of Lemma S66 can be found in Ref. [**?**]. The decomposition for t-doped Gaussian unitaries proved in Theorem S65 implies a similar for t-doped states, as established by the following theorem.

Theorem S67. *Let $n \in \mathbb{N}$ be the number of modes. Let $\kappa, t \in \mathbb{N}$ such that $\kappa t \leq n$. For any (t,κ)-doped Gaussian state vector $|\psi\rangle$ there exists a Gaussian unitary G and a (possibly non-Gaussian) κt-mode state vector $|\phi\rangle$ such that*

$$|\psi\rangle = G\left(|\phi\rangle \otimes |0\rangle^{\otimes(n-\kappa t)}\right). \tag{S344}$$

Moreover, G can be expressed in terms of the first moment and the covariance matrix of $|\psi\rangle$ as follows. The symplectic diagonalisation of the covariance matrix is of the form

$$V(|\psi\rangle\langle\psi|) = S(D \oplus I_{2(n-\kappa t)})S^{\intercal}, \tag{S345}$$

with D being a $2\kappa t \times 2\kappa t$ diagonal matrix and S being a symplectic matrix. Then, $|\psi\rangle$ can be expressed as

$$|\psi\rangle = \hat{D}_{\mathbf{m}(\psi)} U_S |\varphi_{\kappa t}\rangle \otimes |0\rangle^{\otimes(n-\kappa t)}, \tag{S346}$$

for some κt-mode (possibly non-Gaussian) state vector $|\varphi_{\kappa t}\rangle$, where $\hat{D}_{\mathbf{m}(\psi)}$ is the displacement operator and U_S is the Gaussian unitary associated with the symplectic S.

Proof. By definition, any (t,κ)-doped Gaussian state vector $|\psi\rangle$ can be written in terms of a (t,κ)-doped Gaussian unitary U_t as $|\psi\rangle := U_t |0\rangle^{\otimes n}$. Consequently, Theorem S65 implies that

$$\begin{aligned} |\psi\rangle &= G(u_{\kappa t} \otimes \mathbb{1}_{n-\kappa t}) G_{\text{passive}} |0\rangle^{\otimes n} \\ &= G(u_{\kappa t} \otimes \mathbb{1}_{n-\kappa t}) |0\rangle^{\otimes n}, \end{aligned} \tag{S347}$$

where we have used that G_{passive} is passive and thus it preserves the vacuum state, i.e., $G_{\text{passive}} |0\rangle^{\otimes n} = |0\rangle^{\otimes n}$. Hence, (S344) follows by defining $|\phi\rangle := u_{\kappa t} |0\rangle^{\otimes \kappa t}$. Now, let us prove (S346). Note that the first moment and the covariance matrix of the state vector $U_S^\dagger \hat{D}_{\mathbf{m}(\psi)}^\dagger |\psi\rangle$ are given by

$$\begin{aligned} \mathbf{m}\Big(U_S^\dagger \hat{D}_{\mathbf{m}(\psi)}^\dagger |\psi\rangle\langle\psi| \hat{D}_{\mathbf{m}(\psi)} U_S\Big) &= \mathbf{0}_n, \\ V\Big(U_S^\dagger \hat{D}_{\mathbf{m}(\psi)}^\dagger |\psi\rangle\langle\psi| \hat{D}_{\mathbf{m}(\psi)} U_S\Big) &= D \otimes I_{n-\kappa t}, \end{aligned} \tag{S348}$$

and thus

$$\begin{aligned} \mathbf{m}\Big(\mathrm{Tr}_{[\kappa t]}\Big[U_S^\dagger \hat{D}_{\mathbf{m}(\psi)}^\dagger |\psi\rangle\langle\psi| \hat{D}_{\mathbf{m}(\psi)} U_S\Big]\Big) &= \mathbf{0}_{n-\kappa t}, \\ V\Big(\mathrm{Tr}_{[\kappa t]}\Big[U_S^\dagger \hat{D}_{\mathbf{m}(\psi)}^\dagger |\psi\rangle\langle\psi| \hat{D}_{\mathbf{m}(\psi)} U_S\Big]\Big) &= I_{n-\kappa t}. \end{aligned} \tag{S349}$$

Hence, the forthcoming Lemma S68 establishes that

$$\mathrm{Tr}_{[\kappa t]}\Big[U_S^\dagger \hat{D}_{\mathbf{m}(\psi)}^\dagger |\psi\rangle\langle\psi| \hat{D}_{\mathbf{m}(\psi)} U_S\Big] = |0\rangle\langle 0|^{\otimes(n-\kappa t)}. \tag{S350}$$

Consequently, there exists a κt-mode state vector $|\phi\rangle$ such that

$$U_S^\dagger \hat{D}_{\mathbf{m}(\psi)}^\dagger |\psi\rangle = |\phi\rangle \otimes |0\rangle^{\otimes(n-\kappa t)}, \tag{S351}$$

which concludes the proof. □

Lemma S68. *Let ρ be an n-mode state with covariance matrix equal to the identity and first moment equal to $\boldsymbol{r} \in \mathbb{R}^{2n}$,*

$$\begin{aligned} V(\rho) &= I_{2n}\,, \\ \boldsymbol{m}(\rho) &= \boldsymbol{r}\,. \end{aligned} \tag{S352}$$

Then, ρ is the coherent state $\rho = |\mathbf{r}\rangle\langle\mathbf{r}|$, where $|\mathbf{r}\rangle := \hat{D}_{\mathbf{r}}\,|0\rangle^{\otimes n}$.

Proof. It suffices to show that $\mathrm{Tr}[|\mathbf{r}\rangle\langle\mathbf{r}|\,\rho] = 1$, which is valid since it holds that

$$\begin{aligned} 1 &\geq \mathrm{Tr}[|\mathbf{r}\rangle\langle\mathbf{r}|\,\rho] \\ &= \mathrm{Tr}\left[|0\rangle\langle 0|^{\otimes n}\,\hat{D}_{\mathbf{r}}^{\dagger}\rho\hat{D}_{\mathbf{r}}\right] \\ &\geq \mathrm{Tr}\left[\left(\mathbb{1} - \sum_{i=1}^{n} a_i^{\dagger}a_i\right)\hat{D}_{\mathbf{r}}^{\dagger}\rho\hat{D}_{\mathbf{r}}\right] \\ &\overset{\text{(i)}}{=} 1 - \frac{\mathrm{Tr}\left[V\left(\hat{D}_{\mathbf{r}}^{\dagger}\rho\hat{D}_{\mathbf{r}}\right) - I_{2n}\right]}{4} - \frac{\|\mathbf{m}(\hat{D}_{\mathbf{r}}^{\dagger}\rho\hat{D}_{\mathbf{r}})\|_2^2}{2} \\ &= 1 - \frac{\mathrm{Tr}[V(\rho) - I_{2n}]}{4} - \frac{\|\mathbf{m}(\rho) - \mathbf{r}\|_2^2}{2} \\ &= 1\,, \end{aligned} \tag{S353}$$

where in (i) we have used Lemma S48. □

C. t-compressible Gaussian states

In this section, we present the notion of t-compressible states.

Definition S69 (t-compressible states)**.** *Let $t, n \in \mathbb{N}$ with $t \leq n$. An n-mode state vector $|\psi\rangle$ is said to be t-compressible when there exists a Gaussian unitary G and a t-mode state vector $|\phi\rangle$ such that*

$$|\psi\rangle = G\,|\phi\rangle \otimes |0\rangle^{\otimes(n-t)}\,. \tag{S354}$$

The above Gaussian unitary G can be chosen as follows.

Lemma S70 (Choice of states)**.** *Let $|\psi\rangle$ be an n-mode t-compressible state vector. Let*

$$V(|\psi\rangle\langle\psi|) = S\left(D_t \oplus I_{2(n-t)}\right)S^{\intercal}\,. \tag{S355}$$

be the symplectic diagonalisation of the covariance matrix of $V(|\psi\rangle\langle\psi|)$. Then, $|\psi\rangle$ can be written as

$$|\psi\rangle = \hat{D}_{\boldsymbol{m}(|\psi\rangle\langle\psi|)}U_S\,|\phi\rangle \otimes |0\rangle^{\otimes(n-t)}\,, \tag{S356}$$

where $|\phi\rangle$ is a t-mode state vector with first moment and covariance matrix given by

$$\begin{aligned} V(|\phi\rangle\langle\phi|) &= D_t\,, \\ \boldsymbol{m}(|\phi\rangle\langle\phi|) &= 0\,. \end{aligned} \tag{S357}$$

Proof. The proof is the same as the one of Theorem S67. □

As a consequence of Theorem S67, we have the following.

Corollary S71 (Compressibility)**.** *A (t,κ)-doped Gaussian state is κt-compressible.*

Note that the covariance matrix of a t-compressible state has at least $n-t$ symplectic eigenvalues equal to 1. This motivates the following definition, in analogy to the stabiliser dimension [**?**] and fermionic Gaussian dimension [**?**].

Definition S72 (Gaussian dimension of a state)**.** *The Gaussian dimension of a state vector $|\psi\rangle$ is the number of symplectic eigenvalues of the covariance matrix $V(|\psi\rangle\langle\psi|)$ which are equal to one.*

We now observe the following fact.

Lemma S73 (Characterization of t-compressible pure states)**.** *An n-mode pure state is t-compressible if and only if its Gaussian dimension is at least $n-t$.*

Proof. First, let us assume that the Gaussian dimension of the pure state vector $|\psi\rangle$ is $n-t$. Hence, the symplectic diagonalisation of its covariance matrix is of the form

$$V(|\psi\rangle\!\langle\psi|) = S\left(D_t \oplus I_{2(n-t)}\right) S^{\intercal} . \tag{S358}$$

Consequently, it holds that

$$V\Big(\mathrm{Tr}_{[t]}[U_S^\dagger\,|\psi\rangle\!\langle\psi|\,U_S]\Big) = I_{2(n-t)} . \tag{S359}$$

Hence, Lemma S68 establishes that $\mathrm{Tr}_{[t]}[U_S^\dagger\,|\psi\rangle\!\langle\psi|\,U_S] = |\mathbf{r}\rangle\!\langle\mathbf{r}|$ for some $\mathbf{r}\in\mathbb{R}^{2(n-t)}$. Consequently, there exists a t-mode state vector $|\phi\rangle\!\langle\phi|$ such that

$$U_S^\dagger\,|\psi\rangle = |\phi\rangle\otimes|\mathbf{r}\rangle\ . \tag{S360}$$

This implies that

$$|\psi\rangle = U_S(\mathbb{1}_t\otimes\hat{D}_{\mathbf{r}})\,|\phi\rangle\otimes|0\rangle^{\otimes(n-t)}\ , \tag{S361}$$

and in particular that $|\psi\rangle$ is t-compressible. On the other hand, the fact that the Gaussian dimension of a t-compressible state is at least $n-t$ is trivial. □

D. Tomography algorithm for t-compressible Gaussian states

In this subsection, we present a tomography algorithm for learning t-compressible n-mode bosonic states. Thus, because of Theorem S71, this algorithm can also be applied to doped quantum states. Our algorithm is outlined in Table S4, and its correctness is proved in the forthcoming Theorem S74. The algorithm turns out to be efficient for $t = O(1)$ (i.e., it runs in polynomial time in the number of modes), while super-poly (yet sub-exponential) for $t = O(\log n)$. Conversely, in Theorem S80 we show that, in the regime $t = O(\log n)$ *any* tomography algorithm designed to learn t-compressible states must be inefficient.

Our tomography algorithm is motivated by Theorem S67 and consists of two main parts, summarised as follows: In the first part, it estimates the covariance matrix of the unknown state, expresses it in its Williamson decomposition (S65), and uses this decomposition to find a Gaussian unitary $\tilde{G}^\dagger$ that can *approximately* transform the n-mode state into a tensor product between an arbitrary state on the first t modes and the vacuum state in the last $n-t$ modes. In the second part, the algorithm applies $\tilde{G}^\dagger$ to the unknown state. It then measures the occupation number of the last $n-t$ modes, and if the vacuum state is obtained, it performs full state tomography on only the first t modes, resulting in a state vector $|\tilde{\phi}\rangle$. The output of the algorithm will then be a classical representation of the state vector $\tilde{G}(|\tilde{\phi}\rangle\otimes|0\rangle^{\otimes(n-t)})$. Refer to Table S4 for more precise details of the algorithm.

Remarkably, our tomography algorithm is experimentally feasible, as it requires only Gaussian evolutions, avalanche photodiodes (devices capable of reliably distinguishing between zero and one or more photons, commonly referred to as 'on/off detectors' [**?**]), and easily implementable Gaussian measurements, such as homodyne and heterodyne detection [**?**]. Specifically, the estimation of the first moment and covariance matrix in Line 1 relies solely on homodyne measurements [**? ?**]. Line 4 involves only Gaussian unitaries, while Line 5 uses a photon counting that post-selects on the vacuum of the last $n-t$ modes when no photons are detected. This latter measurement can be practically achieved using photodetectors, like avalanche photodiodes, capable of discriminating between zero and one or more photons. Lastly, in Line 8, quantum state tomography of the first t modes is performed. To accomplish this in an experimentally feasible manner, we may utilise the continuous-variable classical shadow algorithm proposed in Ref. [**?**], which relies solely on randomised Gaussian unitaries and homodyne and heterodyne measurements. (Alternatively, in order to improve the performance, we may also consider utilising our tomography algorithm for moment-constrained pure states detailed in Section S2 B. However, despite its better performance, the latter algorithm is not feasible for experimental implementation with our current technology.)

Before delving into the correctness proof of the algorithm, let us establish the notation. Let $N_{\mathrm{cov}}(n,\varepsilon,\delta,E_2)$ be the sample complexity of the algorithm outlined in Table S2 and Theorem S53 to estimate the first moment and the covariance matrix of an unknown n-mode state ρ, subject to the second-moment energy constraint $\mathrm{Tr}[\rho\hat{E}_n^2]\le n^2E_2^2$, with precision ε and failure probability δ. Specifically, thanks to Theorem S53, a number

$$N_{\mathrm{cov}}(n,\varepsilon,\delta,E_2) = O\left(\log\left(\frac{n^2}{\delta}\right)\frac{n^3E_2^2}{\varepsilon^2}\right) \tag{S362}$$

of copies of ρ are sufficient to build a covariance matrix $\tilde{V}$ and a vector $\tilde{m}$ such that

$$\Pr\left(\|\tilde{V}' - V(\rho)\|_\infty \le \varepsilon \quad \text{and} \quad \|\tilde{\mathbf{m}} - \mathbf{m}(\rho)\|_2 \le \varepsilon\right) \ge 1 - \delta\,. \tag{S363}$$

Furthermore, we denote as $N_{\text{tom,CV}}(n, \varepsilon, \delta, E_1)$ the sample complexity of the full state tomography algorithm outlined in Table S1 and Theorem S36 to learn an unknown n-mode pure state ψ, subject to the energy constraint $\text{Tr}[\psi \hat{E}_n] \le nE_1$, with accuracy ε in trace distance and failure probability δ. Specifically, Theorem S36 implies that

$$N_{\text{tom,CV}}(n, \varepsilon, \delta, E_1) = \left\lceil 2^{21} \frac{d_{\text{eff}}(n, \varepsilon, E_1)}{\varepsilon^2} \log\left(\frac{4}{\delta}\right) \right\rceil = O\left(\frac{E_1}{\varepsilon^2}\right)^n \tag{S364}$$

copies of ψ are sufficient to generate a classical representation (i.e., $d_{\text{eff}}(n, \varepsilon, E_1)$-sized vector) of a pure state $\tilde{\psi}$ such that

$$\Pr\left[\frac{1}{2}\|\psi - \tilde{\psi}\|_1 \le \varepsilon\right] \ge 1 - \delta, \tag{S365}$$

where $d_{\text{eff}}(n, \varepsilon, E_1) \le \left(\frac{eE_1}{\varepsilon^2} + 2e\right)^n$.

Theorem S74 (Number of copies required)**.** *Let $n, t \in \mathbb{N}$ with $t \le n$, and $E \ge 0$. Let $|\psi\rangle$ be an n-mode t-compressible state satisfying the second-moment constraint $\text{Tr}[|\psi\rangle\!\langle\psi|\, \hat{E}_n^2] \le n^2 E^2$. Let $\varepsilon, \delta \in (0,1)$, and N such that*

$$N := \; N_1 + \left\lceil 2N_2 + 24 \log\left(\frac{3}{\delta}\right) \right\rceil = O\left(\frac{n^9 E^6}{\varepsilon^4}\right) + O\left(\left(\frac{nE^2}{\varepsilon^2}\right)^t\right)\,, \tag{S369}$$

where

$$N_1 := N_{\text{cov}}\left(n, \frac{\varepsilon^2}{2(n+1)(1+4nE)^2}, \frac{\delta}{3}, E\right) \tag{S370}$$

is reported in (S362) and $N_2 := N_{\text{tom,CV}}\left(t, \frac{\varepsilon}{2}, \frac{\delta}{3}, 80n^2E^2\right)$ is reported in (S364). Then, N copies of $|\psi\rangle$ are sufficient to build a classical description of a t-compressible pure state vector $|\hat{\psi}\rangle$ such that

$$\Pr\left(\frac{1}{2}\left\| |\hat{\psi}\rangle\!\langle\hat{\psi}| - |\psi\rangle\!\langle\psi| \right\|_1 \le \varepsilon\right) \ge 1 - \delta\,. \tag{S371}$$

Such a classical description consists of the triplet $\left(\tilde{\boldsymbol{m}}, \tilde{S}, |\tilde{\phi}_1\rangle\right)$, which defines $|\hat{\psi}\rangle$ as

$$|\hat{\psi}\rangle := \hat{D}_{\tilde{\boldsymbol{m}}} U_{\tilde{S}} |\tilde{\phi}_1\rangle \otimes |0\rangle^{\otimes(n-t)}\,, \tag{S372}$$

where $\tilde{\boldsymbol{m}} \in \mathbb{R}^{2n}$, $\tilde{S} \in \text{Sp}(\mathbb{R}^{2n})$, and $|\tilde{\phi}_1\rangle$ is a t-mode pure state contained in a $\left\lceil \left(\frac{e(80n^2E^2 - \frac{1}{2})}{\varepsilon^2}\right)^t \right\rceil$-dimensional subspace.

Before proving Theorem S74 let us state some useful lemmas.

Lemma S75 (Bounds to symplectic matrices)**.** *Let $V(\rho)$ be a covariance matrix associated with an n-mode state ρ and let $V(\rho) = SDS^\intercal$ its symplectic diagonalisation. It holds that*

$$\|S\|_\infty \le \sqrt{\|V(\rho)\|_\infty}. \tag{S373}$$

In particular, it holds that

$$\|V(\rho)\|_\infty \le 4\,\text{Tr}[\rho \hat{E}_n] \tag{S374}$$

and consequently $\|S\|_\infty \le \sqrt{4\,\text{Tr}[\rho \hat{E}_n]}$.

Proof. Since $D \ge \mathbb{1}$, we have that $V(\rho) \ge SS^\intercal$. Consequently, it holds that

$$\begin{aligned} \|S\|_\infty^2 &= \|SS^\intercal\|_\infty \\ &\le \|V(\rho)\|_\infty \\ &\le \text{Tr}\, V(\rho) \\ &= 4\,\text{Tr}[\rho \hat{E}_n] - 2\|\mathbf{m}(\rho)\|_2^2 \\ &\le 4\,\text{Tr}[\rho \hat{E}_n]\,, \end{aligned} \tag{S375}$$

where we have exploited Lemma S48. This implies $\|S\|_\infty \le \sqrt{4\,\text{Tr}[\rho \hat{E}_n]}$, where $\hat{E}_n := \frac{1}{2}\hat{\mathbf{R}}^\intercal \hat{\mathbf{R}}$ is the energy operator. □

Table S4. Tomography algorithm for bosonic t-compressible pure states

Input: Accuracy ε, failure probability δ, second moment upper bound E, N copies of the unknown n-mode t-compressible state vector $|\psi\rangle$ satisfying the 2-moment constraint $\mathrm{Tr}\left[\hat{E}_n^2 |\psi\rangle\langle\psi|\right] \leq n^2E^2$, where

$$\begin{aligned} N :=\ & N_1 + \left\lceil 2N_2 + 24\log\left(\frac{3}{\delta}\right)\right\rceil \\ =\ & O\left(\frac{n^9E^6}{\varepsilon^4}\right) + O\left(\left(\frac{n^2E^2}{\varepsilon^2}\right)^t\right), \end{aligned} \tag{S366}$$

where $N_1 := N_{\mathrm{cov}}\left(n, \frac{\varepsilon^2}{2(n+1)(1+4nE)^2}, \frac{\delta}{3}, E\right)$ is reported in (S362) and $N_2 := N_{\mathrm{tom,CV}}\left(t, \frac{\varepsilon}{2}, \frac{\delta}{3}, 80n^2E^2\right)$ is reported in (S364).
Output: A classical description of a pure state vector $|\hat{\psi}\rangle$ such that

$$\Pr\left(\frac{1}{2}\left\||\hat{\psi}\rangle\langle\hat{\psi}| - |\psi\rangle\langle\psi|\right\|_1 \leq \varepsilon\right) \geq 1-\delta\,. \tag{S367}$$

Such a classical description consists of the triplet $(\tilde{\mathbf{m}}, \tilde{S}, |\tilde{\phi}_1\rangle)$ which defines $|\hat{\psi}\rangle$ via the relation

$$|\hat{\psi}\rangle := \hat{D}_{\tilde{\mathbf{m}}} U_{\tilde{S}} |\tilde{\phi}_1\rangle \otimes |0\rangle^{\otimes(n-t)}\,, \tag{S368}$$

where $\tilde{\mathbf{m}} \in \mathbb{R}^{2n}$, $\tilde{S} \in \mathrm{Sp}(\mathbb{R}^{2n})$, and $|\tilde{\phi}_1\rangle$ is a t-mode pure state contained in a $\left\lceil\left(\frac{e(80n^2E^2-\frac{1}{2})}{\varepsilon^2}\right)^t\right\rceil$-dimensional subspace.

1: Query N_1 copies of $|\psi\rangle$ and apply the algorithm in Table S2 to construct a vector $\tilde{\mathbf{m}}$ and a covariance matrix $\tilde{V}$ which are estimates of the first moment $\mathbf{m}(|\psi\rangle\langle\psi|)$ and of the covariance matrix $V(|\psi\rangle\langle\psi|)$, respectively.
2: Compute the symplectic diagonalisation $\tilde{V} = \tilde{S}\tilde{D}\tilde{S}^\intercal$, where $\tilde{S} \in \mathrm{Sp}(2n)$ and $\tilde{D} := \mathrm{diag}(\tilde{d}_1, \tilde{d}_1, \tilde{d}_2, \tilde{d}_2, \ldots, \tilde{d}_n, \tilde{d}_n)$, with $\tilde{d}_1 \geq \tilde{d}_2 \geq \ldots \geq \tilde{d}_n \geq 1$.
3: **for** $k \leftarrow 1$ **to** $\lceil 2N_2 + 24\log(\frac{3}{\delta})\rceil$ **do**
4: Query one copy of $|\psi\rangle$ and apply $\hat{D}^\dagger_{\tilde{\mathbf{m}}} U^\dagger_{\tilde{S}}$, obtaining the state vector $|\tilde{\psi}_t\rangle := \hat{D}^\dagger_{\tilde{\mathbf{m}}} U^\dagger_{\tilde{S}} |\psi\rangle$.
5: Measure $|\tilde{\psi}_t\rangle$ with respect the POVM $\{M_0 := \mathbb{1}_t \otimes |0\rangle\langle 0|^{\otimes(n-t)}, M_1 := \mathbb{1} - M_0\}$, and discard if the outcome corresponds to the POVM element M_1. The post-measurement state is thus of the form $|\tilde{\phi}\rangle \otimes |0\rangle^{\otimes(n-t)}$, where $|\tilde{\phi}\rangle$ is the pure state proportional to $\langle 0|^{\otimes(n-t)} |\tilde{\psi}_t\rangle$.
6: Do a step of the algorithm in Table S1 to perform pure-state tomography of the t-mode state vector $|\tilde{\phi}\rangle$, which has mean energy upper bounded by $80n^2E^2$.
7: **end for**
8: The tomography algorithm of Line 6 returns a classical description of a t-mode pure state vector $|\tilde{\phi}_1\rangle$, which is supported on a $\left\lceil\left(\frac{e(80n^2E^2-\frac{1}{2})}{\varepsilon^2}\right)^t\right\rceil$-dimensional subspace.
9: **return** the triplet $\left(\tilde{\mathbf{m}}, \tilde{S}, |\tilde{\phi}_1\rangle\right)$.

Lemma S76 (Perturbation on symplectic diagonalisation [?]). *Let $V_1, V_2 \in \mathbb{R}^{2n\times 2n}$ be two covariance matrices with symplectic diagonalisations $V_1 = S_1D_1S_1^\intercal$ and $V_2 = S_2D_2S_2^\intercal$, where the elements on the diagonal of D_1 and D_2 are arranged in descending order. Then*

$$\|D_1 - D_2\|_\infty \leq \sqrt{K(V_1)K(V_2)}\|V_1 - V_2\|_\infty\,, \tag{S376}$$

where $K(V)$ is the condition number of the covariance matrix V, defined as $K(V) := \|V\|_\infty \|V^{-1}\|_\infty$.

Lemma S77 Inverse of a covariance matrix. *Let V be a covariance matrix. The inverse of the covariance matrix V^{-1} satisfies*

$$\|V^{-1}\| \leq \|V\| \tag{S377}$$

where $\|\cdot\|$ is an orthogonal invariant matrix norm. As a consequence, the condition number of V, which is defined as $K(V) := \|V\|_\infty\|V^{-1}\|_\infty$, can be upper bounded as

$$K(V) \leq \|V\|^2_\infty\,. \tag{S378}$$

Proof. Let $V = SDS^\intercal$ be the symplectic diagonalisation of V with S symplectic and D diagonal of the form $D = \mathrm{diag}(d_1, d_1, d_2, d_2, \dots, d_n, d_n)$. In particular, we recall that $S\Omega_n S^\intercal = \Omega_n$ and that D commutes with Ω_n. Then it holds that

$$\begin{aligned} V^{-1} &= (S^{-1})^\intercal D^{-1} S^{-1} \\ &= \Omega_n\Omega_n (S^{-1})^\intercal D^{-1} S^{-1}\Omega_n\Omega_n \\ &= \Omega_n S\Omega_n D^{-1}\Omega_n S^\intercal \Omega_n \\ &= -\Omega_n S D^{-1} S^\intercal \Omega_n \\ &= \Omega_n S D^{-1} (\Omega_n S)^\intercal . \end{aligned} \tag{S379}$$

Since V is a covariance matrix, then $D \geq \mathbb{1}$ and thus $D^{-1} \leq D$. Consequently, the matrix inequality

$$\begin{aligned} V^{-1} &\leq \Omega_n S D (\Omega_n S)^\intercal \\ &= \Omega_n V \Omega_n^\intercal \end{aligned} \tag{S380}$$

holds. Since Ω_n is orthogonal, then for any orthogonal invariant norm $\|\cdot\|$ it holds that $\|V^{-1}\| \leq \|V\|$[**?**], that concludes the proof. □

Lemma S78 (Upper bound to mean energy)**.** *Let ρ be an n-mode state. Let $S \in \mathrm{Sp}(2n)$ be a symplectic matrix. Let U_S be the Gaussian symplectic unitary associated with S. Then, the mean energy of $U_S\rho U_S^\dagger$ can be upper bounded as*

$$E\Big(U_S\rho U_S^\dagger\Big) \leq \|S\|_\infty^2 E(\rho)\,, \tag{S381}$$

where $E(\rho) \coloneqq \mathrm{Tr}\Big[\frac{\hat{\boldsymbol{R}}^\intercal\hat{\boldsymbol{R}}}{2}\,\rho\Big]$.

Proof. It holds that

$$\begin{aligned} E\Big(U_S\rho U_S^\dagger\Big) &= \frac{1}{2}\,\mathrm{Tr}\Big[U_S\rho U_S^\dagger \hat{\mathbf{R}}^\intercal\hat{\mathbf{R}}\Big] \\ &\overset{\text{(i)}}{=} \frac{1}{2}\,\mathrm{Tr}\Big[\rho\hat{\mathbf{R}}^\intercal S^\intercal S\hat{\mathbf{R}}\Big] \\ &\overset{\text{(ii)}}{=} \frac{1}{2}\,\mathrm{Tr}\Big[\rho\hat{\mathbf{R}}^\intercal O_1^\intercal Z^2 O_1\hat{\mathbf{R}}\Big] \\ &\overset{\text{(iii)}}{=} \frac{1}{2}\,\mathrm{Tr}\Big[U_{O_1}\rho U_{O_1}^\dagger \hat{\mathbf{R}}^\intercal Z^2\hat{\mathbf{R}}\Big] \\ &\overset{\text{(iv)}}{\leq} \|S\|_\infty^2\,\mathrm{Tr}\Big[U_{O_1}\rho U_{O_1}^\dagger \hat{E}\Big] \\ &\overset{\text{(v)}}{=} \|S\|_\infty^2\,\mathrm{Tr}\Big[\rho\hat{E}\Big] . \end{aligned} \tag{S382}$$

Here, in (i), we have used that $U_S^\dagger\hat{\mathbf{R}}U_S = S\mathbf{R}$. In (ii), we have exploited the Euler decomposition $S = O_1 Z O_2$ given in (S69). In (iii), we have used $U_{O_1}^\dagger\hat{\mathbf{R}}U_{O_1} = O_1\mathbf{R}$. In (iv), we first used that $\hat{\mathbf{R}}^\intercal Z^2\hat{\mathbf{R}} = \sum_{i=1}^{2n} z_i^2\hat{R}_i^2 \leq \|Z\|_\infty^2\hat{\mathbf{R}}^\intercal\hat{\mathbf{R}}$, and, second, we have exploited that $\|S\|_\infty^2 = \|S^\intercal S\|_\infty = \|O_2^\intercal Z^2 O_2\|_\infty = \|Z\|_\infty^2$ to obtain that $\hat{\mathbf{R}}^\intercal Z^2\hat{\mathbf{R}} \leq 2\|S\|_\infty^2\hat{E}$. Finally, in (v) we have used again $U_{O_1}^\dagger\hat{\mathbf{R}}U_{O_1} = O_1\mathbf{R}$ and the fact that O_1 is orthogonal. □

Lemma S79 (Refined mean energy bound)**.** *Let ρ be an n-mode state. Let $\mathbf{r} \in \mathbb{R}^{2n}$ and let $\hat{D}_\mathbf{r}$ be the associated displacement operator. Then, the mean energy of $\hat{D}_\mathbf{r}\rho\hat{D}_\mathbf{r}^\dagger$ reads*

$$E\Big(\hat{D}_\mathbf{r}\rho\hat{D}_\mathbf{r}^\dagger\Big) = E(\rho) + \mathbf{r}^\intercal\mathbf{m}(\rho) + \frac{1}{2}\|\mathbf{r}\|_2^2\,, \tag{S383}$$

where $E(\rho) \coloneqq \mathrm{Tr}\Big[\frac{\hat{\boldsymbol{R}}^\intercal\hat{\boldsymbol{R}}}{2}\,\rho\Big]$. *In particular, it can be upper bounded as*

$$E\Big(\hat{D}_\mathbf{r}\rho\hat{D}_\mathbf{r}^\dagger\Big) \leq E(\rho) + \sqrt{2E(\rho)}\|\boldsymbol{r}\|_2 + \frac{1}{2}\|\boldsymbol{r}\|_2^2\,. \tag{S384}$$

Proof. It holds that

$$\begin{aligned} E\left(\hat{D}_{\mathbf{r}}\rho\hat{D}_{\mathbf{r}}^{\dagger}\right) &= \frac{1}{2}\operatorname{Tr}[\rho(\hat{\mathbf{R}}+\mathbf{r}\mathbb{1})^{\intercal}(\hat{\mathbf{R}}+\mathbf{r}\mathbb{1})] \\ &= E(\rho)+\mathbf{r}^{\intercal}\mathbf{m}(\rho)+\frac{1}{2}\|\mathbf{r}\|_2^2\,. \end{aligned} \tag{S385}$$

Moreover, note that

$$m_i(\rho)=\operatorname{Tr}[\rho R_i]\le\sqrt{\operatorname{Tr}[\rho R_i^2]} \tag{S386}$$

for each $i\in[2n]$, where we have exploited (S7). Consequently, it holds that

$$\|\mathbf{m}(\rho)\|_2\le\sqrt{\sum_{i=1}^{n}\operatorname{Tr}[\rho R_i^2]}=\sqrt{2E(\rho)}\,. \tag{S387}$$

Thus, it follows that

$$\begin{aligned} E\left(\hat{D}_{\mathbf{r}}\rho\hat{D}_{\mathbf{r}}^{\dagger}\right) &\le E(\rho)+\|\mathbf{r}\|_2\|\mathbf{m}(\rho)\|_2+\frac{1}{2}\|\mathbf{r}\|_2^2 \\ &\le E(\rho)+\sqrt{2E(\rho)}\|\mathbf{r}\|_2+\frac{1}{2}\|\mathbf{r}\|_2^2\,. \end{aligned} \tag{S388}$$

□

Now, we are ready to prove Theorem S74.

Proof of Theorem S74. We aim to establish the correctness of the algorithm presented in Table S4.

In Line 1, we use $N_{\text{cov}}\left(n,\varepsilon_{\text{cov}},\frac{\delta}{3},E\right)$ copies of $|\psi\rangle$ in order to build a vector $\tilde{\mathbf{m}}\in\mathbb{R}^{2n}$ and a covariance matrix $\tilde{V}\in\mathbb{R}^{2n,2n}$ such that

$$\Pr\left(\|\tilde{V}-V(|\psi\rangle\!\langle\psi|)\|_\infty\le\varepsilon_{\text{cov}}\quad\text{and}\quad\|\tilde{\mathbf{m}}-\mathbf{m}(|\psi\rangle\!\langle\psi|)\|_2\le\varepsilon_{\text{cov}}\right)\ge1-\frac{\delta}{3}\,, \tag{S389}$$

where $N_{\text{cov}}(\cdot)$ is defined in (S362) and the accuracy ε_{cov} will be fixed later. From now on, let us assume that we are in the probability event in which

$$\|\tilde{V}-V(|\psi\rangle\!\langle\psi|)\|_\infty\le\varepsilon_{\text{cov}}\quad\text{and}\quad\|\tilde{\mathbf{m}}-\mathbf{m}(|\psi\rangle\!\langle\psi|)\|_2\le\varepsilon_{\text{cov}}\,. \tag{S390}$$

In Line 2, we compute the symplectic diagonalisation $\tilde{V}=\tilde{S}\tilde{D}\tilde{S}^{\intercal}$, where $\tilde{S}\in\operatorname{Sp}(2n)$ and

$$\tilde{D}\coloneqq\operatorname{diag}\left(\tilde{d}_1,\tilde{d}_1,\tilde{d}_2,\tilde{d}_2,\dots,\tilde{d}_n,\tilde{d}_n\right) \tag{S391}$$

with $\tilde{d}_1\ge\tilde{d}_2\ge\ldots\ge\tilde{d}_n\ge1$.

The sequence of steps in Lines 4, 5, and 6 have to be repeated a number

$$\left\lceil 2N_{\text{tom,CV}}\left(t,\varepsilon_{\text{tom}},\frac{\delta}{3},E\right)+24\log\left(\frac{3}{\delta}\right)\right\rceil \tag{S392}$$

of times, where the quantity $N_{\text{tom,CV}}\left(t,\varepsilon_{\text{tom}},\frac{\delta}{3},E\right)$ is the number of copies sufficient for tomography of a t-mode pure state with mean energy per mode at most E with accuracy ε_{tom} and failure probability $\frac{\delta}{3}$ (see (S364)). The accuracy ε_{tom} and the energy E will be fixed later.

In Line 4, we query one copy of $|\psi\rangle$ and apply $\hat{D}^{\dagger}_{\tilde{\mathbf{m}}}U^{\dagger}_{\tilde{S}}$, obtaining the state vector $|\tilde{\psi}_t\rangle\coloneqq\hat{D}^{\dagger}_{\tilde{\mathbf{m}}}U^{\dagger}_{\tilde{S}}|\psi\rangle$. Since $|\psi\rangle$ is t-compressible and since it holds that $\tilde{\mathbf{m}}\simeq\mathbf{m}(|\psi\rangle\!\langle\psi|)$ and $\tilde{V}=\tilde{S}\tilde{D}\tilde{S}^{\intercal}\simeq V(|\psi\rangle\!\langle\psi|)$, we intuitively expect that the reduced state of $|\tilde{\psi}_t\rangle$ onto the last $n-t$ modes is very close to the vacuum $|0\rangle^{\otimes(n-t)}$ (as we are going to rigorously show).

In Line 5, we measure $|\tilde{\psi}_t\rangle$ with respect the POVM $\{\mathbb{1}_t\otimes|0\rangle\!\langle0|^{\otimes(n-t)},\mathbb{1}-\mathbb{1}_t\otimes|0\rangle\!\langle0|^{\otimes(n-t)}\}$, and discard the copies associated with the outcome corresponding to the POVM element $\mathbb{1}-\mathbb{1}_t\otimes|0\rangle\!\langle0|^{\otimes(n-t)}$. The post-measurement state is not discarded with probability

$$P_{\text{succ}}\coloneqq\operatorname{Tr}\left[\mathbb{1}_t\otimes|0\rangle\!\langle0|^{\otimes(n-t)}\;|\tilde{\psi}_t\rangle\!\langle\tilde{\psi}_t|\right]\,, \tag{S393}$$

and the remaining copies are thus in a state vector of the form $|\tilde{\phi}\rangle \otimes |0\rangle^{\otimes(n-t)}$, where $|\tilde{\phi}\rangle$ is given by

$$|\tilde{\phi}\rangle := \frac{\langle 0|^{\otimes(n-t)}\,|\tilde{\psi}_t\rangle}{\sqrt{P_{\text{succ}}}}\,. \tag{S394}$$

Let us now find a lower bound on the probability of success P_{succ}. For simplicity in the following we will use the notation $\psi := |\psi\rangle\!\langle\psi|$, $\tilde{\psi}_t := |\psi\rangle\!\langle\psi|$ and $\tilde{\phi} := |\tilde{\phi}\rangle\!\langle\tilde{\phi}|$. Note that

$$\begin{aligned}
P_{\text{succ}} &= \mathrm{Tr}[\mathbb{1}_t \otimes |0\rangle\!\langle 0|^{\otimes(n-t)}\ \tilde{\psi}_t] \\
&= \mathrm{Tr}[|0\rangle\!\langle 0|^{\otimes(n-t)}\ \mathrm{Tr}_{[t]}\,\tilde{\psi}_t] \\
&\overset{\text{(i)}}{\ge} \mathrm{Tr}\left[\left(\mathbb{1} - \sum_{i=t+1}^{n} a_i^\dagger a_i\right)\ \mathrm{Tr}_{[t]}\,\tilde{\psi}_t\right] \\
&\overset{\text{(ii)}}{=} 1 - \frac{\mathrm{Tr}[V(\mathrm{Tr}_{[t]}\,\tilde{\psi}_t) - I_{n-t}]}{4} - \frac{\|\mathbf{m}(\mathrm{Tr}_{[t]}\,\tilde{\psi}_t)\|_2^2}{2} \\
&\ge 1 - \frac{2(n-t)\|V(\mathrm{Tr}_{[t]}\,\tilde{\psi}_t) - I_{n-t}\|_\infty}{4} - \frac{\|\mathbf{m}(\tilde{\psi}_t)\|_2^2}{2}\,.
\end{aligned} \tag{S395}$$

Here, in (i), we have used the following operator inequality

$$\mathbb{1}_t \otimes |0\rangle\!\langle 0|^{\otimes(n-t)} \ge \mathbb{1} - \sum_{i=t+1}^{n} a_i^\dagger a_i\,, \tag{S396}$$

which can be readily verified. In (ii), we have exploited Lemma S48. Moreover, note that

$$\begin{aligned}
V(\tilde{\psi}_t) &= V\!\left(U_{\tilde{S}}^\dagger \hat{D}_{\tilde{\mathbf{m}}}^\dagger \psi \hat{D}_{\tilde{\mathbf{m}}} U_{\tilde{S}}\right) \\
&= \tilde{S}^{-1} V(\psi) (\tilde{S}^{-1})^\intercal \\
&= \tilde{S}^{-1}\left(V(\psi) - \tilde{V}\right)(\tilde{S}^{-1})^\intercal + \tilde{D}\,.
\end{aligned} \tag{S397}$$

Since ψ is t-compressible, Lemma S70 establishes that $|\psi\rangle$ can be written as

$$|\psi\rangle = \hat{D}_{\mathbf{m}(\psi)} U_S\, |\phi_t\rangle \otimes |0\rangle^{\otimes(n-t)}\,, \tag{S398}$$

where $|\phi_t\rangle$ is some t-mode state vector, S is a symplectic matrix such that $V(\psi) = S\,(D_t \oplus \mathbb{1}_{n-t})\,S^\intercal$, and D_t is $2t \times 2t$ diagonal matrix of the form

$$D_t := \mathrm{diag}\,(d_1, d_1, d_2, d_2, \ldots, d_n, d_n) \tag{S399}$$

with $d_1 \ge d_2 \ge \ldots \ge d_n \ge 1$. Let us analyse the first term in the r.h.s. of (S395). It holds that

$$\begin{aligned}
\|V(\mathrm{Tr}_{[t]}\,\tilde{\psi}_t) - I_{n-t}\|_\infty &\overset{\text{(iii)}}{=} \left\|\left[\tilde{S}^{-1}\left(V(\psi) - \tilde{V}\right)(\tilde{S}^{-1})^\intercal\right]_{n-t} + [\tilde{D}]_{n-t} - I_{n-t}\right\|_\infty \\
&\le \left\|\left[\tilde{S}^{-1}\left(V(\psi) - \tilde{V}\right)(\tilde{S}^{-1})^\intercal\right]_{n-t}\right\|_\infty + \left\|[\tilde{D}]_{n-t} - I_{n-t}\right\|_\infty \\
&\overset{\text{(iv)}}{\le} \left\|\tilde{S}^{-1}\left(V(\psi) - \tilde{V}\right)(\tilde{S}^{-1})^\intercal\right\|_\infty + \left\|\tilde{D} - D\right\|_\infty \\
&\overset{\text{(v)}}{\le} \|\tilde{S}\|_\infty^2 \|V(\psi) - \tilde{V}\|_\infty + \left\|\tilde{D} - D\right\|_\infty \\
&\overset{\text{(vi)}}{\le} \|\tilde{V}\|_\infty \varepsilon_{\text{cov}} + \left\|\tilde{D} - D\right\|_\infty \\
&\overset{\text{(vii)}}{\le} (\varepsilon_{\text{cov}} + \|V(\psi)\|_\infty)\varepsilon_{\text{cov}} + \sqrt{K(V(\psi))K(\tilde{V})}\|\tilde{V} - V(\psi)\|_\infty \\
&\overset{\text{(viii)}}{\le} \left(\varepsilon_{\text{cov}} + 4\,\mathrm{Tr}[\psi \hat{E}_n]\right)\varepsilon_{\text{cov}} + \|V(\psi)\|_\infty \|\tilde{V}\|_\infty \varepsilon_{\text{cov}} \\
&\overset{\text{(ix)}}{\le} \left(\varepsilon_{\text{cov}} + 4\,\mathrm{Tr}[\psi \hat{E}_n]\right)\varepsilon_{\text{cov}} + 4\,\mathrm{Tr}[\psi \hat{E}_n]\left(4\,\mathrm{Tr}[\psi \hat{E}_n] + \varepsilon_{\text{cov}}\right)\varepsilon_{\text{cov}} \\
&\overset{\text{(x)}}{\le} (\varepsilon_{\text{cov}} + 4nE)\,(1 + 4nE)\,\varepsilon_{\text{cov}} \\
&\le (1 + 4nE)^2\,\varepsilon_{\text{cov}}\,.
\end{aligned} \tag{S400}$$

Here, in (iii), we have exploited (S400) and we have introduced the notation $[A]_k$ to denote the $k \times k$ lower-right block of the matrix A. In (iv), first, we have used that for any $n \times n$ matrix A and any $k \le n$ it holds that $\|[A]_k\|_\infty \le \|A\|_\infty$, and, second, we have exploited that $[D]_{n-t} = I_{n-t}$. In (v), we first used the submultiplicativity of the operator norm, and second, we applied Lemma S51, which implies that $\|\tilde{S}^{-1}\|_\infty = \|\tilde{S}\|_\infty$. In (vi), we have used that $\|\tilde{S}\|_\infty \le \sqrt{\|\tilde{V}\|_\infty}$, as established by Lemma S75, and that $\|V(\psi) - \tilde{V}\|_\infty \le \varepsilon_V$. In (vii), first we have used that $\|\tilde{V}\|_\infty \le \|V(\psi)\|_\infty + \varepsilon_V$, and second, we have exploited Lemma S76 that establishes that the maximum difference between the ordered symplectic eigenvalues $\|\tilde{D} - D\|_\infty$ can be upper bounded in terms of the *condition numbers* $K(V(\psi)), K(\tilde{V})$ (see Lemma S76) and in terms of $\|V(\psi) - \tilde{V}\|_\infty$. In (viii), we have used that $K(V) \le \|V\|_\infty^2$, as stated by Lemma S77. In (ix), we have exploited that $\|V(\psi)\|_\infty \le 4\,\mathrm{Tr}[\psi \hat{E}_n]$, as established by Lemma S75. Finally, in (x), we have used Cauchy Schwarz inequality to deduce that $\mathrm{Tr}[\psi \hat{E}_n] \le \sqrt{\mathrm{Tr}[\psi \hat{E}_n^2]}$ and then we have used the assumption that $\sqrt{\mathrm{Tr}[\psi \hat{E}_n^2]} \le nE$.

Now, let us analyse the second term in the r.h.s. of (S395). With similar techniques of (S400), we can upper bound $\|\mathbf{m}(\tilde{\psi}_t)\|_2$ as

$$
\begin{aligned}
\|\mathbf{m}(\tilde{\psi}_t)\|_2 &= \left\|\mathbf{m}\left(U_{\tilde{S}}^\dagger \hat{D}_{\tilde{\mathbf{m}}}^\dagger \psi \hat{D}_{\tilde{\mathbf{m}}} U_{\tilde{S}}\right)\right\|_2 \\
&= \left\|\tilde{S}^{-1}\left(\mathbf{m}(\psi) - \tilde{\mathbf{m}}\right)\right\|_2 \\
&\le \|\tilde{S}^{-1}\|_\infty \|\mathbf{m}(\psi) - \tilde{\mathbf{m}}\|_2 \\
&\le \|\tilde{S}\|_\infty \, \varepsilon_{\text{cov}} \\
&\le \sqrt{\|\tilde{V}\|_\infty} \varepsilon_{\text{cov}} \\
&\le \sqrt{4nE + \varepsilon_{\text{cov}}}\, \varepsilon_{\text{cov}} \\
&\le \sqrt{4nE + 1}\, \varepsilon_{\text{cov}} \,.
\end{aligned}
\tag{S401}
$$

By putting (S395), (S400), and (S401) together, we deduce that P_{succ} is lower bounded by

$$
\begin{aligned}
P_{\text{succ}} &\ge 1 - \frac{2(n-t)\|V(\mathrm{Tr}_{[t]}\,\tilde{\psi}_t) - \mathbb{1}\|_\infty}{4} - \frac{\|\mathbf{m}(\tilde{\psi}_t)\|_2^2}{2} \\
&\ge 1 - \frac{n-t}{2}(1+4nE)^2 \varepsilon_{\text{cov}} - \frac{1}{2}(1+4nE)\varepsilon_{\text{cov}}^2 \\
&\ge 1 - \frac{(1+4nE)^2}{2}(n\,\varepsilon_{\text{cov}} + \varepsilon_{\text{cov}}^2) \\
&\ge 1 - \frac{(1+4nE)^2(n+1)}{2}\varepsilon_{\text{cov}} \,.
\end{aligned}
\tag{S402}
$$

The choice of ε_{cov}, which we will make later in (S410), implies that $P_{\text{succ}} \ge \frac{3}{4}$. Consequently, thanks Lemma S5, since we conduct in total $\left\lceil 2N_{\text{tom,CV}}\left(t, \varepsilon_{\text{tom}}, \frac{\delta}{3}, E\right) + 24\log\left(\frac{3}{\delta}\right)\right\rceil$ measurements, each of which with a success probability of $P_{\text{succ}} \ge \frac{3}{4}$, the probability of obtaining at least $N_{\text{tom,CV}}\left(t, \varepsilon_{\text{tom}}, \frac{\delta}{3}, E\right)$ successful outcomes is $\ge 1 - \frac{\delta}{3}$. In other words, the probability of getting at least $N_{\text{tom,CV}}\left(t, \varepsilon_{\text{tom}}, \frac{\delta}{3}, E\right)$ post-measurement state vectors $|\tilde{\phi}\rangle \otimes |0\rangle^{\otimes(n-t)}$ is $\ge 1 - \frac{\delta}{3}$. From now on, let us assume that we are in such a probability event. In Line 6, we do a step of the algorithm in Table S1 to perform pure-state tomography of the energy-constrained t-mode state vector $|\tilde{\phi}\rangle$, by considering the first t modes of the post-measurement state vector $|\tilde{\phi}\rangle \otimes |0\rangle^{\otimes(n-t)}$. In order to apply such an algorithm we need to find an upper bound on the mean energy of $|\tilde{\phi}\rangle$. By denoting the mean energy of a

state ψ as $E(\psi)$, let us note that

$$
\begin{aligned}
E(\tilde{\phi}) &\overset{\text{(i)}}{\le} \frac{1}{P_{\text{succ}}} E(\tilde{\psi}_t) \\
&\overset{\text{(ii)}}{\le} \frac{\|\tilde{S}\|_\infty^2}{P_{\text{succ}}} E\left(\hat{D}_{\tilde{\mathbf{m}}}^\dagger \left|\psi\right\rangle\!\left\langle\psi\right| \hat{D}_{\tilde{\mathbf{m}}}\right) \\
&\overset{\text{(iii)}}{\le} \frac{\|\tilde{S}\|_\infty^2}{P_{\text{succ}}} \left[E(\psi) + \sqrt{2E(\psi)}\|\tilde{\mathbf{m}}\|_2 + \frac{1}{2}\|\tilde{\mathbf{m}}\|_2^2\right] \\
&\overset{\text{(iv)}}{\le} \frac{\|\tilde{S}\|_\infty^2}{P_{\text{succ}}} \left[E(\psi) + \sqrt{2E(\psi)}(\|\mathbf{m}(\psi)\|_2 + \varepsilon_m) + \frac{1}{2}(\|\mathbf{m}(\psi)\|_2 + \varepsilon_m)^2\right] \\
&\overset{\text{(v)}}{\le} \frac{4E(\psi) + \varepsilon_V}{P_{\text{succ}}} \left[E(\psi) + \sqrt{2E(\psi)}(\sqrt{2E(\psi)} + \varepsilon_m) + \frac{1}{2}(\sqrt{2E(\psi)} + \varepsilon_m)^2\right] \\
&\overset{\text{(vi)}}{\le} \frac{4}{3}[4E(\psi) + \varepsilon_V]\left[4E(\psi) + 2\sqrt{2E(\psi)}\varepsilon_m + \frac{\varepsilon_m^2}{2}\right] \\
&\overset{\text{(vii)}}{\le} 80\,[E(\psi)]^2 \\
&\le 80\ \mathrm{Tr}[\psi \hat{E}_n^2] \\
&\le 80\, E^2 n^2 .
\end{aligned}
\tag{S403}
$$

Here, in (i), we have used that

$$
\begin{aligned}
E(\tilde{\phi}) &= \frac{1}{P_{\text{succ}}} E\left(\langle 0|^{\otimes(n-t)}\, \tilde{\psi}_t\, |0\rangle^{\otimes(n-t)}\right) \\
&= \frac{1}{2P_{\text{succ}}}\ \mathrm{Tr}\left[\sum_{i=1}^t R_i^2\, \langle 0|^{\otimes(n-t)}\, \tilde{\psi}_t\, |0\rangle^{\otimes(n-t)}\right] \\
&= \frac{1}{2P_{\text{succ}}}\ \mathrm{Tr}\left[\left(\sum_{i=1}^t R_i^2\right) \otimes |0\rangle\!\langle 0|^{\otimes(n-t)}\, \tilde{\psi}_t\right] \\
&\le \frac{1}{2P_{\text{succ}}}\ \mathrm{Tr}\left[\left(\sum_{i=1}^t R_i^2\right) \otimes \mathbb{1}_{n-t}\, \tilde{\psi}_t\right] \\
&= \frac{1}{2P_{\text{succ}}}\ \mathrm{Tr}\left[\left(\sum_{i=1}^t R_i^2\right) \tilde{\psi}_t\right] \\
&\le \frac{1}{2P_{\text{succ}}}\ \mathrm{Tr}\left[\left(\sum_{i=1}^n R_i^2\right) \tilde{\psi}_t\right] \\
&= \frac{E(\tilde{\psi}_t)}{P_{\text{succ}}} .
\end{aligned}
\tag{S404}
$$

In (ii), first we have used that $\tilde{\psi} = U_{\tilde{S}}^\dagger \hat{D}_{\tilde{\mathbf{m}}}^\dagger \psi \hat{D}_{\tilde{\mathbf{m}}} U_{\tilde{S}}$, and then we have used Lemma S78. In (iii), we have used Lemma S79. Then, in (iv), we have used triangular inequality and the bound $\|\mathbf{m}(\psi) - \tilde{\mathbf{m}}\|_2 \le \varepsilon_{\text{cov}}$. In (v), we have used Lemma S75, together with $\|\tilde{V} - V\|_\infty \le \varepsilon_{\text{cov}}$ and (S387). In (vi), we have exploited that $P_{\text{succ}} \ge \frac{3}{4}$. In (vii), we have exploited that $\varepsilon_{\text{cov}} \le 1$ and $E(\psi) \ge \frac{1}{2}$. Consequently, the mean energy per mode of the state $\tilde{\phi}$ is upper bounded by

$$
\frac{E(\tilde{\phi})}{t} \le \frac{80\, E^2 n^2}{t} \le 80\, E^2 n^2 , \tag{S405}
$$

and hence we can set $E \coloneqq 80\, E^2 n^2$.

In Line 8, the tomography algorithm called in the iterations of Line 6 outputs a t-mode state vector $|\tilde{\phi}_1\rangle$, which is supported on a $\left\lceil \left(\frac{e(80n^2E^2 - \frac{1}{2})}{\varepsilon^2}\right)^t \right\rceil$-dimensional subspace of $L^2(\mathbb{R}^t)$. Since the total number of copies of $|\tilde{\phi}\rangle$ employed in such a tomography algorithm is $\ge N_{\text{tom,CV}}\left(t, \varepsilon_{\text{tom}}, \frac{\delta}{3}, E\right)$, the state vector $|\tilde{\phi}_1\rangle$ satisfies

$$
\Pr\left(\frac{1}{2}\left\| |\tilde{\phi}\rangle\!\langle\tilde{\phi}| - |\tilde{\phi}_1\rangle\!\langle\tilde{\phi}_1| \right\|_1 < \varepsilon_{\text{tom}}\right) \ge 1 - \frac{\delta}{3} . \tag{S406}
$$

From now on, let us assume that we are in the probability event in which

$$\frac{1}{2}\left\||\tilde{\phi}\rangle\langle\tilde{\phi}| - |\tilde{\phi}_1\rangle\langle\tilde{\phi}_1|\right\|_1 < \varepsilon_{\text{tom}}\,. \tag{S407}$$

In Line 9, we output a classical description of the n-mode state vector $|\hat{\psi}\rangle$, which is defined as

$$|\hat{\psi}\rangle := \hat{D}_{\tilde{\mathbf{m}}} U_{\tilde{S}}\left(|\tilde{\phi}_1\rangle \otimes |0\rangle^{\otimes(n-t)}\right)\,. \tag{S408}$$

Note that

$$\begin{aligned}\frac{1}{2}\left\||\hat{\psi}\rangle\langle\hat{\psi}| - |\psi\rangle\langle\psi|\right\|_1 &\overset{\text{(i)}}{=} \frac{1}{2}\left\||\tilde{\phi}_1\rangle\langle\tilde{\phi}_1| \otimes |0\rangle\langle 0|^{\otimes(n-t)} - |\tilde{\psi}_t\rangle\langle\tilde{\psi}_t|\right\|_1 \\ &\overset{\text{(ii)}}{\le} \frac{1}{2}\left\||\tilde{\phi}_1\rangle\langle\tilde{\phi}_1| - |\tilde{\phi}\rangle\langle\tilde{\phi}|\right\|_1 + \frac{1}{2}\left\||\tilde{\phi}\rangle\langle\tilde{\phi}| \otimes |0\rangle\langle 0|^{\otimes(n-t)} - |\tilde{\psi}_t\rangle\langle\tilde{\psi}_t|\right\|_1 \\ &\overset{\text{(iii)}}{\le} \varepsilon_{\text{tom}} + \sqrt{1 - P_{\text{succ}}} \\ &\overset{\text{(iv)}}{\le} \varepsilon_{\text{tom}} + (1 + 4nE)\sqrt{\frac{n+1}{2}\varepsilon_{\text{cov}}}\,.\end{aligned} \tag{S409}$$

Here, in (i), we have used unitarily invariance of the trace norm. In (ii), we have used the triangular inequality of the trace norm. In (iii), we have used (S407) and the *gentle measurement lemma* [**?**]. Finally, in (iv) we have used (S402). Consequently, by choosing

$$\begin{aligned}\varepsilon_{\text{cov}} &:= \frac{\varepsilon^2}{2(n+1)(1+4nE)^2}\,, \\ \varepsilon_{\text{tom}} &:= \frac{\varepsilon}{2}\,,\end{aligned} \tag{S410}$$

we have that

$$\frac{1}{2}\left\||\hat{\psi}\rangle\langle\hat{\psi}| - |\psi\rangle\langle\psi|\right\|_1 \le \varepsilon\,. \tag{S411}$$

By using an union bound, we can conclude that a total number of

$$N_{\text{cov}}\left(n, \varepsilon_{\text{cov}}, \frac{\delta}{3}, E\right) + \left\lceil 2N_{\text{tom,CV}}\left(t, \varepsilon_{\text{tom}}, \frac{\delta}{3}, E\right) + 24\log\left(\frac{3}{\delta}\right)\right\rceil \tag{S412}$$

copies of $|\psi\rangle$ suffices to construct a classical representation of a state vector $|\hat{\psi}\rangle$ such that

$$\Pr\left(\frac{1}{2}\left\||\hat{\psi}\rangle\langle\hat{\psi}| - |\psi\rangle\langle\psi|\right\|_1 \le \varepsilon\right) \ge 1 - \delta\,. \tag{S413}$$

□

We now show that any algorithm to learn t-compressible states should be inefficient if t scales faster than a constant in the number of modes.

Theorem S80 (Lower bound on sample complexity of tomography of t-compressible states)**.** *Let us consider a tomography algorithm that learns, within a trace distance $\le \varepsilon$ and failure probability $\le \delta$, an unknwon n-mode t-compressible state ψ satisfying the second-moment constraint $\sqrt{\mathrm{Tr}[\psi \hat{E}_n^2]} \le nE$. Then, such a tomography algorithm must use a number of state copies N satisfying*

$$\begin{aligned}N &\ge \frac{1}{t\, g\left(\frac{n}{t}\left(E - \frac{1}{2}\right)\right)}\left[2(1-\delta)\left(\frac{\frac{n}{t}\left(E - \frac{1}{2}\right)}{12\varepsilon} - \frac{1}{t}\right)^t - (1-\delta)\log_2(32\pi) - H_2(\delta)\right] \\ &= \Theta\left(\frac{nE}{t\varepsilon}\right)^t\,.\end{aligned} \tag{S414}$$

Here, $g(x) := (x+1)\log_2(x+1) - x\log_2 x$ is the bosonic entropy, and $H_2(x) := -x\log_2 x - (1-x)\log_2(1-x)$ is the binary entropy.

Proof. Since the tomography algorithm can learn arbitrary n-mode t-compressible states ψ satisfying the second-moment constraint $\sqrt{\mathrm{Tr}[\psi \hat{E}_n^2]} \leq nE$, then it can also learn *arbitrary* t-mode state vectors $|\phi_t\rangle$ satisfying

$$\sqrt{\mathrm{Tr}[|\phi_t\rangle\langle\phi_t|\, \hat{N}_t^2]} \leq n\left(E - \frac{1}{2}\right) . \tag{S415}$$

Indeed, $|\psi\rangle \coloneqq |\phi_t\rangle \otimes |0\rangle^{\otimes(n-t)}$ is a t-compressible state with second-moment upper bounded by

$$\begin{aligned}
\sqrt{\mathrm{Tr}\left[|\psi\rangle\langle\psi|\, \hat{E}_n^2\right]} &= \sqrt{\mathrm{Tr}\left[|\phi_t\rangle\langle\phi_t| \otimes |0\rangle\langle 0|^{\otimes(n-t)}\, \hat{E}_n^2\right]} \\
&= \sqrt{\mathrm{Tr}\left[|\phi_t\rangle\langle\phi_t| \otimes |0\rangle\langle 0|^{\otimes(n-t)} \left(\hat{N}_n + \frac{n}{2}\mathbb{1}\right)^2\right]} \\
&= \sqrt{\mathrm{Tr}[|\phi_t\rangle\langle\phi_t|\, \hat{N}_t^2] + n\,\mathrm{Tr}[|\phi_t\rangle\langle\phi_t|\, \hat{N}_t] + \frac{n^2}{4}} \\
&\leq \sqrt{\mathrm{Tr}[|\phi_t\rangle\langle\phi_t|\, \hat{N}_t^2] + n\sqrt{\mathrm{Tr}[|\phi_t\rangle\langle\phi_t|\, \hat{N}_t^2]} + \frac{n^2}{4}} \\
&= \sqrt{\mathrm{Tr}[|\phi_t\rangle\langle\phi_t|\, \hat{N}_t^2]} + \frac{n}{2} \\
&\leq nE\,.
\end{aligned} \tag{S416}$$

Finally, Theorem S26 ensures that any algorithm that learns unknown t-mode states $|\phi_t\rangle$, satisfying the second-moment constraint

$$\frac{1}{t}\sqrt{\mathrm{Tr}[|\phi_t\rangle\langle\phi_t|\, \hat{N}_t^2]} \leq \frac{n}{t}\left(E - \frac{1}{2}\right), \tag{S417}$$

must use a number of state copies N that satisfies the condition in (S414). □
